# Processes of consent in research for adults with impaired mental capacity nearing the end of life: systematic review and transparent expert consultation (MORECare_Capacity statement)

**DOI:** 10.1186/s12916-020-01654-2

**Published:** 2020-07-22

**Authors:** C. J. Evans, E. Yorganci, P. Lewis, J. Koffman, K. Stone, I. Tunnard, B. Wee, W. Bernal, M. Hotopf, I. J. Higginson, on behalf of MORECare_Capacity, Deborah Tanner, Claire Henry, Gunn Grande, Steve Dewar, Gareth Owen, Rachel Burman, Dimitrios Adamis, Michael Dunn, Scott Kim, Simon Woods, Rowena Vohora

**Affiliations:** 1 Cicely Saunders Institute of Palliative Care, Policy & Rehabilitation, Florence Nightingale Faculty of Nursing, Midwifery & Palliative Care, King’s College London, Bessemer Road, London, SE5 9PJ UK; 2 Sussex Community NHS Foundation Trust, Brighton General Hospital, Brighton, UK; 3 Centre of Medical Law and Ethics, The Dickson Poon School of Law, King’s College London, London, UK; 4 Oxford University Hospitals NHS Foundation Trust and Harris Manchester College, University of Oxford, Oxford, UK; 5 King’s College Hospital, London, UK; 6 Psychological Medicine, Institute of Psychiatry, Psychology & Neuroscience, King’s College London, London, UK

**Keywords:** Palliative care, Terminal care, Decision-making, Consent, Methods, Ethics, Systematic review, Consensus

## Abstract

**Background:**

Involving adults lacking capacity (ALC) in research on end of life care (EoLC) or serious illness is important, but often omitted. We aimed to develop evidence-based guidance on how best to include individuals with impaired capacity nearing the end of life in research, by identifying the challenges and solutions for processes of consent across the capacity spectrum.

**Methods:**

Methods Of Researching End of Life Care_Capacity (MORECare_C) furthers the MORECare statement on research evaluating EoLC. We used simultaneous methods of systematic review and transparent expert consultation (TEC). The systematic review involved four electronic databases searches. The eligibility criteria identified studies involving adults with serious illness and impaired capacity, and methods for recruitment in research, implementing the research methods, and exploring public attitudes. The TEC involved stakeholder consultation to discuss and generate recommendations, and a Delphi survey and an expert ‘think-tank’ to explore consensus. We narratively synthesised the literature mapping processes of consent with recruitment outcomes, solutions, and challenges. We explored recommendation consensus using descriptive statistics. Synthesis of all the findings informed the guidance statement.

**Results:**

Of the 5539 articles identified, 91 met eligibility. The studies encompassed people with dementia (27%) and in palliative care (18%). Seventy-five percent used observational designs. Studies on research methods (37 studies) focused on processes of proxy decision-making, advance consent, and deferred consent. Studies implementing research methods (30 studies) demonstrated the role of family members as both proxy decision-makers and supporting decision-making for the person with impaired capacity. The TEC involved 43 participants who generated 29 recommendations, with consensus that indicated. Key areas were the timeliness of the consent process and maximising an individual’s decisional capacity. The think-tank (*n* = 19) refined equivocal recommendations including supporting proxy decision-makers, training practitioners, and incorporating legislative frameworks.

**Conclusions:**

The MORECare_C statement details 20 solutions to recruit ALC nearing the EoL in research. The statement provides much needed guidance to enrol individuals with serious illness in research. Key is involving family members early and designing study procedures to accommodate variable and changeable levels of capacity. The statement demonstrates the ethical imperative and processes of recruiting adults across the capacity spectrum in varying populations and settings.

## Background

There is an urgent need for evidence on best practice in palliative care. The projected increases in global serious health-related suffering demand immediate action. By 2060, an estimated 48 million people will die globally with serious related-suffering, representing an 87% increase from the 26 million in 2016 [1]. Failing to respond will see 80% of people globally with little or no access to palliative care services and treatment [2]. A major barrier in research on palliative care is ethical concerns about the perceived vulnerability of adults with serious illness and including them in research, particularly if the person also has impaired mental capacity [3]. Exclusion of adults with impaired capacity to consent for themselves impedes evidence-based care and treatment that is applicable across the illness trajectory and end of life (EoL) [4]. New interventions require robust evaluation to examine benefit and potential of harm for the population intending to benefit [5, 6]. Studies, especially clinical trials in palliative care, are often compromised by insufficient sample size to detect change [7–13], and impaired understanding of legislation governing research involving adults with impaired capacity [14]. The ethical challenges of recruiting individuals with impaired capacity are examined across fields involving adults with serious illness including palliative care [15], dementia [16–18], mental health [19], and intensive care [20]. Systematic reviews have considered consent processes in specific conditions (e.g. dementia [21], schizophrenia [22]) and aspects of involving adults lacking capacity in research (e.g. capacity assessment [22], enhancing informed consent with older people [23, 24], and strategies for designing research studies [25] and increasing the recruitment rate in palliative care [26]). But, in palliative care, intervention studies are few and often exclude adults lacking capacity, for example in the dying phase [27]. There is literature from both within and outside the field of palliative care that could inform much needed guidance on best practice on processes of consent across the capacity spectrum in serious illness. This study aimed to determine how best to include individuals with impaired capacity in research on EoLC by identifying challenges for and solutions to processes of consent across the capacity spectrum. This paper reports the integrated results from a systematic review and transparent expert consultation to form the MORECare_Capacity statement on processes of consent in research on EoLC. This furthers the Methods Of Researching End of Life Care (MORECare) statement on evaluating complex circumstances in EoLC [28] by giving detailed consideration on processes of consent for adults with serious illness across the capacity trajectory. The MORECare statement omitted this area, focusing on outcome measurement, response shift and attrition, integrating mixed methods and economic evaluation.

In this paper, ‘capacity’ refers to mental capacity to make an informed decision regarding research participation. ‘The spectrum of capacity’ of individuals ranges from potentially impaired, and anticipated to have impaired capacity, to lacking capacity. The legislation governing involvement of adults lacking capacity in research and terminology is jurisdiction specific. In this paper, the term ‘consultee’ (someone who has capacity) is used to encompass the different terms used in respective jurisdictions including but not limited to proxy-decision maker, personal consultee and nominated consultee. A distinction is made between a personal consultee (e.g. family member) and a nominated consultee (e.g. health professional) [29]. ‘The process of consent’ refers to the steps taken to ensure that an eligible research participant is sufficiently informed about the purposes, content, affiliations of the study, and their right to withdraw from the study at any point, enabling them or their consultee to decide freely about research participation [30].

## Methods

### Study design

**Fig. 1:**
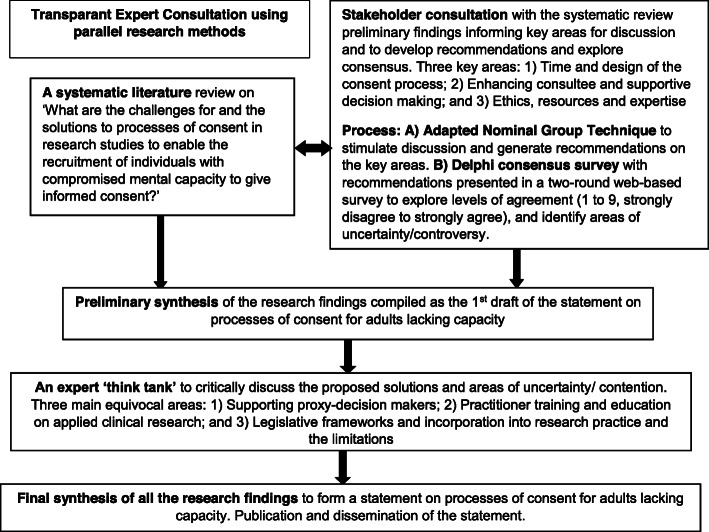
Overview of the study design

We used a parallel iterative research design detailed in Fig. 1. We used methods of systematic literature review to identify and map the challenges and solutions for processes of consent for adults with impaired capacity, and the MORECare transparent expert consultation (TEC) to debate key areas [28] of uncertainty/contention. The TEC involved expert stakeholder consultations using consensus methods of modified nominal group technique to generate recommendations [31], and then presenting the recommendations in an online Delphi survey to explore levels of agreement [32]. We held a final expert think-tank to explore areas of contention/uncertainty and synthesise the findings to develop the statement. King’s College London Research Ethics Committee approved the TEC component (ref no. BDM/10/11-90).

### Systematic review

#### Design

We used systematic review method of narrative synthesis to systematically identify, appraise, and synthesise quantitative and qualitative literature [33]. Methods of analysis and inclusion criteria were pre-defined in the study protocol. Reporting followed the PRISMA guidance [34] (see Additional file 1: Table S1).

#### Eligibility criteria

##### Population

Adults (≥ 18 years old) with impaired capacity encompassing declining capacity (e.g. mild to moderate dementia), fluctuating capacity (e.g. delirium), and lack of capacity (e.g. dying, advanced dementia) are included.

##### Context

The scoping of the literature identified areas recruiting adults lacking capacity with serious illness in research. We included studies from palliative care, mental health (delirium, dementia, learning disabilities), or emergency medicine/critical care.

##### Interest

Studies discussing consent in its various forms (e.g. informed, advanced, proxy) and impaired mental capacity are included. We did not restrict by health or behaviour outcome. We focused on research studies investigating either of the following: (i) methods for involving adults with impaired capacity in research, (ii) implementing research methods to enable recruitment, or (iii) exploring public attitudes and ethical issues on involvement in research.

##### Design

Randomised controlled trials (RCTs) and quasi-experimental, mixed method, or observational qualitative or quantitative designs are included. We included published study protocols that reported the methods of consent to enhance understanding for the main study. We excluded systematic reviews but used relevant systematic reviews for reference chaining by screening the cited publications for eligibility. We excluded opinion pieces and commentaries and non-English language papers. Studies concerning treatment/clinical decision-making or bioethics were out of scope.

### Search strategy

We developed search strategies for each of the population groups—palliative care, mental health (including dementia), and emergency medicine/critical care. MeSH terms for the palliative care group included ‘Terminally ill’ or ‘Palliative Care’ AND ‘Decision making’ or ‘Mental Competency’ AND ‘Informed consent’ or ‘Third-party consent’. Key search terms were used as free-text, and with use of truncation symbol to retrieve variations in the terminology. Search terms were piloted pre-study and mapped to assess their relevance and specificity, and refined working with a specialist librarian (see Additional file 2: Table S2 - electronic search terms). We searched four electronic databases: MEDLINE (1966–Present), EMBASE (1947–Present), PsychINFO (1887–Present), and CINAHL (1937–Present), and supplemented with referencing chain, grey literature electronic survey, and expert recommendations. The last search was run on 30 October 2018.

#### Quality appraisal

Study risk of bias was assessed using the validated QualSyst review tool suitable for quantitative and qualitative studies [35] by one reviewer (EY, IT, CJE), and a random 10% sample checked by a second author (CJE). Scores that diverged by > 10% were discussed within the research team. A single quality appraisal was undertaken for studies reported in multiple publications (e.g. protocol paper and a main trial results paper). The QualSyst assessment criteria include 14 items for quantitative studies and 10 items for qualitative studies. Each item is scored from 0 to 2 (0, not present; 1, partial; 2, yes; or not applicable). The percentage of the total possible score indicates the quality grade: < 50%, low; > 50 and < 70%, medium; or > 70%, high. Study designs were categorised using the Cochrane Effective Practice and Organisation of Care (EPOC) grade system [36] (see Additional file 2: Table S3).

#### Data screening, extraction, and analysis

Referencing software (Endnote version x8) [37] was used to manage a database of search findings and remove duplicates. Title and abstract screen by one reviewer (EY/KS) and second independent review of 20% to test the application of the eligibility criteria (CJE). Titles/abstracts that met the review criteria, or if insufficient information to determine eligibility, were subject to full-text screening. Full-text articles were single-screened by three reviewers (EY/KS/CJE). Full-text papers with uncertain eligibility were reviewed by two reviewers and eligibility agreed (EY/KS with CJE). A standardised data extraction form was developed and piloted based on the Cochrane Consumers and Communication Revie Group’s data extraction template (see Additional file 2: Table S4). Data included the study design and aim, the population and context, method(s) of consent, recruitment rate, and challenges and solutions. Study data were extracted by one reviewer (CJE, EY, KS) and checked by a second reviewer (CJE, EY, KS). We contacted two authors to check availability of the publications in English. Using narrative synthesis [33], textual descriptions from extracted data for all studies were mapped to form matrices for studies innovating research method, studies using innovative methods, or considered ethics, legislation, or public attitudes. Each matrix was analysed and coded in Microsoft Excel using thematic analysis to explore prominent themes. Higher quality studies were valued with a greater strength in the final synthesis.

### Transparent expert consultation

The TEC aimed to enhance the systematic review findings by exploring the application of the findings in research studies and areas little considered or uncertain in the evidence. The TEC explored researchers’ and service users’ perspectives on recruiting individuals with impaired capacity in research on EoLC. The TEC sought to generate recommendations on processes of consent to enable recruitment and explore the level of consensus.

#### Setting and participants

Participants were purposively sampled based on their expertise in conducting research involving adults with impaired capacity (including ethicists), caring for patients with advanced disease, or a service user/carer (e.g. palliative care services), or a voluntary sector representative (e.g. Alzheimer’s Society). Participants were invited to the workshops held in the Cicely Saunders Institute, King’s College London. Eligible participants included members of the project’s expert panel (project applicants), Project Advisory Group (invited experts in, for example, ethics, and PPI and voluntary sector representatives) [see the “Acknowledgements” section], respondents in the systematic review grey literature survey and invited ethicists, clinicians, commissioners, researchers, members of ethical committees, policymakers, and service user and lay voluntary sector representatives. Identified professional participants received email invitations for the workshop. Service user and lay voluntary sector representatives were recruited via voluntary sector groups including, for example, Alzheimer’s Society and Independent Cancer Patients’ Voice. The respective organisations circulated the invitation letter to their members targeting those known to have an interest/experience of either a carer for an adult with impaired capacity, being a patient with progressive illness, or supporting research involving adults with impaired capacity.

The TEC used four stages:

*Stage I: Identifying critical issues*. The initial workshop focused on critical issues identified from the systematic review preliminary findings and expert opinion (e.g. areas with limited empirical evidence and relevance in the processes of consent for adults across the capacity spectrum).

*Stage II: Stakeholder workshop*. Participants received a pre-workshop briefing pack detailing the aim, critical issues, and workshop format. The workshop comprised presentations on the critical areas overviewing findings from the systematic review followed by structured group discussion involving 10–14 participants focusing on one of the critical areas. Group discussions were digitally recorded. We used a structured nominal group process facilitated by a member of the research team. The facilitator guided participants through a structured process of (1) a brief discussion, (2) individual writing of recommendations and ranking, and (3) participants in turn stating their highest ranked recommendations until individual lists were exhausted (or time exceeded) [31]. Scribes wrote the recommendations and ranking on a flipchart, and each small group discussed and agreed on the final priority order, then presented and discussed with the whole group. Participants individually listed and ranked recommendations from one to five (highest to lowest) on structured A4 sheets detailing the respective group question, ranking scale and boxes to list recommendations, rank, and detail rationale.

*Stage III: Delphi online consensus exercise*. This is a two-round online consensus exercise [32]. Recommendations generated in the workshop were posted online to the workshop participants, members of the expert panel and Project Advisory Group, and respondents to the grey literature survey. Participants received a personalised email invitation and reminder after 2 weeks. The online participants anonymously ranked, from one to nine (strongly disagree to strongly agree), the extent they agreed with a recommendation and used free-text spaces to comment on each recommendation. Findings from round 1 informed requirements to revise recommendations where comments suggested ambiguity. Round 2 re-presented the revised recommendations and the median score for each recommendation from round 1. Participants again indicated their level of agreement ranked from one to nine and provided free-text commentary on, for example, rationale for ranking score.

*Stage IV: Expert ‘think-tank’*. The expert ‘think tank’ workshop aimed to aid data synthesis and inform the solutions and recommendations in the statement by critically considering areas of contention/uncertainty identified in the consensus exercise and systematic review findings. The think-tank aimed to understand the debates surrounding these areas, the strengths and limitations of the evidence, and the solutions for practice. Participants were purposively selected from the workshop participants based on expertise, e.g. ethicists, lay voluntary sector representative, researcher, and clinician. Think-tank participations received a briefing report that summarised for the respective area the systematic review findings on the challenges and solutions identified in the evidence base, and the recommendations and level of agreement from the consensus exercise. The think-tank used a format of presentations and debate, drawing on structured nominal group process to facilitate participant agreement on the top two or three key solutions for each area, and commentary on their thinking. Participants discussed and debated these areas in groups of 6–7. Discussions were digitally recorded, and scribes recorded on flipchart the key debates.

#### Data analysis

Individual recommendations from the workshop and their ranking were entered in Excel spreadsheets with assigned participant identification numbers. Two researchers (CJE, KS) coded and arranged recommendations by themes, duplicates were combined, and recommendations arranged by priority ranking (1 highest to 5 lowest). Free-text comments were collated. Digital recordings were reviewed to inform understanding on the recommendations and debates presented, with key points noted on the Excel spreadsheet for the respective recommendation. The recommendations retained participants’ original language where possible with amendments guided by the expert panel to enhance clarity and avoid repetition. The final recommendations were those ranked the highest (≤ 3) and reviewed and agreed by the expert panel and piloted (e.g. for clarity), and then posted on the online consensus survey. Analysis of the consensus survey-scaled data used descriptive statistics (frequencies and medians) and plots (box and whisker plots) of interquartile ranges to analyse and interpret levels of agreement. We used a conventional categorisation to interpret agreement (indicated, equivocal, or not indicated) and strength of agreement (strict or broad) used in previous consensus studies [28]. Table 1 details the categories by the respective median region and IQR [38]. Narrative comments were collated by recommendation, and themes identified to understand the issues raised and provide illustrative examples [32].

**Table 1:** Levels of consensus and agreement by median regions and IQR [38]

Median regions and IQR	Interpretation
**7–9**	Recommendation indicated
**4–6**	Recommendation equivocal
**1–3**	Recommendation not indicated
**IQR in*one*region**	Strict agreement for recommendation
**IQR in*any*three-point region**	Broad agreement for recommendation

*IQR* interquartile range

## Results

### Systematic review search results

**Fig. 2:**
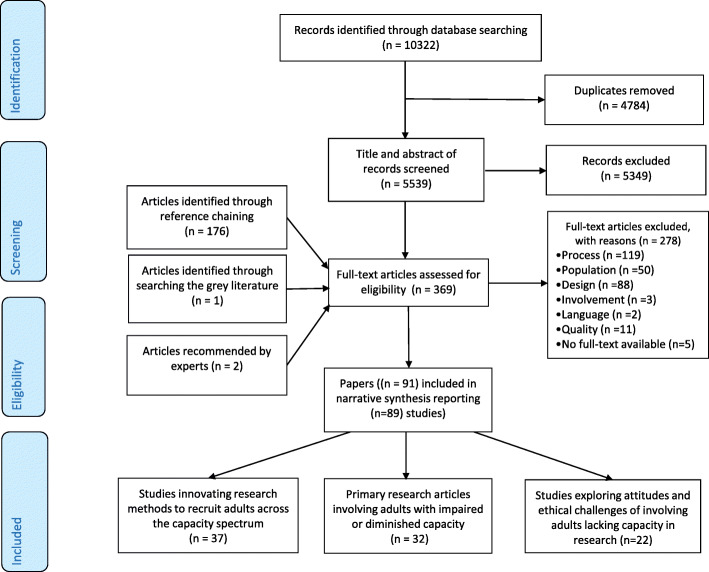
PRISMA flow diagram

The electronic database searches identified 5539 abstracts after removal of duplicates with a further 179 publications identified from other sources (see Fig. 2). Ninety-one publications met the eligibility criteria, reporting 89 studies. Two studies included a protocol and main results papers [39–42]. Studies were conducted mainly in dementia (*n* = 23), palliative care (*n* = 16), and intensive care (*n* = 15) (Table 2). Publications increased over time with the majority published after 2010 (*n* = 54). Studies were conducted mainly in the USA (*n* = 35), UK (*n* = 29), or Canada (*n* = 9) (see Table 2 and Table 3, and Additional file 3: Table S8). The studies formed three main areas of (1) innovating research methods to recruit adults across the capacity spectrum, (2) applying consent processes across the capacity spectrum in studies on serious illness, and (3) public attitudes on involving adults lacking capacity in research.

**Table 2:** Population of interest categorised by the study focus

Patient population of interest	Study focus on processes of consent			
	*Innovating research methods* (*n* = 37)	*Applying research methods* (*n* = 30)	*Attitudes and ethical considerations* (*n* = 22)	*Total studies* (*n* = 89)*
*Palliative care/cancer*	6 [43–48]	8 [49–56]	2 [57, 58]	16
*Dementia*	13 [59–71]	10 [40, 42, 72–79]	1 [80]	24
*Geriatric care*	2 [81, 82]	3 [83–85]	0	5
*Parkinson’s disease*	1 [86]	0	0	1
*Cerebral ischaemic stroke*	2 [87, 88]	0	1 [89]	3
*Mental health*	6 [90–95]	2 [96, 97]	1 [98]	9
*Delirium*	2 [99, 100]	2 [101, 102]	0	4
*Intensive care*	5 [103–107]	5 [108–112]	4 [113–116]	14
*General population*	0	0	13 [117–129]	13

*89 studies are reported from 91 publications (includes two study protocols [40, 42] reported with the main study papers [39, 41]

**Table 3:** Studies innovating research methods to recruit adults across the capacity trajectory (grouped by solutions) (*n* = 37 studies)

Authors, country, EPOC grade	Year	Study design and aim	Setting	Sample description	Consent process for adults across the capacity spectrum	No. patients/eligible (%)	Key findings, challenges, and solutions
Enhanced informed consent processes							
Dobratz et al. [43], USA, B3	2003	Retrospective research study with a vulnerable population to describe issues and dilemmas related to non-participation, attrition, and need for assistance in research with vulnerable home hospice participants.	Home or preferred setting of home hospice agency recipients from two metropolitan settings	**Palliative care:** home hospice agency recipients	**Informed consent:** study was explained to all participants over the phone. Participants provided ‘telephone consent’. During the study visit, participants provided informed consent**.** **Adults with declining capacity**	97/113 (86%)	**Key findings:** five people who agreed to take part unable to provide informed consent due to distress; five other participants with cognitive impairment precluded informed consent. **Solutions:** participants require regular monitoring of their physical and psychological symptoms, oversampling to anticipate and plan for withdrawals, and cognitive assessment tool for all potential participants regardless of diagnosis (e.g. brain metastases), and careful screening of psychological behaviours to reduce distress. Participant positive feedback about the study.
Siminoff et al. [71], the Netherlands, C1	2004	Qualitative study examining the factors that were important in making research participation decisions among patients with Alzheimer’s disease (AD), cancer, critically ill children.	Unclear	**Dementia:** patients with Alzheimer’s disease with cognitive impairment	**Informed consent:** potential subjects were given information about the study by phone. During the conversation, if patients met initial eligibility criteria and expressed continued interest in participation, attended the clinic for formal informed consent procedure. The consent form was read to the subjects and questions were addressed throughout and at the end of reviewing the written consent form. **Adults with declining capacity**	46 AD patients, mean age 72, 91.3% white, 61% female, 78.3% married, 63.6% more than a high school education, 65.6% income > $25,000	**Key findings:** key elements of informed consent. Information—the AD (mean = 1.78) group received more information about voluntary participation than the paediatric (mean = 1.27) group (*F* = 4.1; *p* < .05). Confidentiality—were most likely discussed with the AD group (87%) versus the cancer (22%) and paediatric (9.2%) groups (*χ*^2^ = 85.24; *p* < .001). Discussion of ‘no treatment’ as a viable option occurred most often in the AD group (58.7%) versus the adult cancer (41.6%) and paediatric (3.9%) subjects (*χ*^2^ = 46.38; *p* < .001). AD subjects received the most information about voluntary participation and confidentiality, and no treatment option. **Challenge:** lack of discussions about confidentiality and no treatment as an option.
Buckles et al. [59], USA, B3	2003	Longitudinal study of healthy ageing and dementia to evaluate understanding of informed consent by older participants across a range of dementia severity using a brief test on the elements of informed consent for a low-risk study.	Not stated	**Dementia:** 415 participants, 165 without dementia, 250 with dementia	**Informed consent and assessment dementia severity** with Clinical Dementia Rating (CDR), MMSE **Adults with declining capacity**	415	**Key findings:** after adjusting for education, performance on the test varied with dementia severity in mean differences and by correlation. All non-demented and very mildly demented participants and 92% of mildly demented participants provided correct answer for at least 8 of 10 true-false items, whereas only 67% of the moderately demented participants achieved this level of accuracy. **Solutions:** by moderate dementia stage, involvement of a responsible caregiver in consent process should be mandatory.
Mangset et al. [103], Norway, C1	2008	Qualitative study to explore critically ill patients’ experience with the principle of informed consent in a clinical trial and their ability to give valid informed consent.	2 Norwegian hospitals	**Stroke:** stroke patients who were invited to take part in an international stroke trial	**Informed consent** **Adults with declining capacity**	11	**Key findings:** the results challenge the validity of informed consent for an experimental trial obtained from critically ill patients, and the concept of the consent as a contract obtained on a rational basis between equal and autonomous parties. **Challenges:** most patients did not understand the purpose of randomisation or the concept of clinical equipoise. The main reasons for consent were patients’ dependency on the doctor, their need for a trustful relationship, and seeing study information as a recommendation.
Chouliara et al. [44], UK, C1	2004	Individual interviews on some ethical and methodological challenges involved in conducting research with older people with cancer by referring to researchers’ experiences in an on-going research project.	Care of the elderly wards and a cancer centre	**Cancer:** people with cancer > 65 years old	**Informed consent:** obtained after explaining the project verbally and written. Family members involved to assist patient decision-making. In instances where they felt like the patient was not understanding, they also involved. **Adults with declining capacity**	37/50 (74%)	**Key findings:** mean age 74.3 (SD = 7.5), MMSE 24.0 (4.1)—mild dementia. Involvement of vulnerable, elderly individuals was considered ethical in a low-risk qualitative study. **Challenges:** Fluctuating capacity, fatigue, frailty, physical and cognitive limitations. **Solutions:** a semi-structured interview schedule allowed patients to talk freely. A rigorous procedure to obtain valid consent, including the viewpoints of all the parties involved.
Mittal et al. [61], USA, B3	2007	RCT comparing two processes of consent to evaluate the feasibility, acceptability, and preliminary efficacy of two enhanced consent procedures for patients with Alzheimer’s disease or mild cognitive impairment that used either a PowerPoint presentation or printed consent form.	2 medical centres	**Dementia:** referred patients with possible or probable Alzheimer’s disease (MMSE ≥ 19), mild cognitive impairment	**Enhanced informed consent. PowerPoint slideshow presentation (no written consent form) (SSP)** or **enhanced written consent procedure** (researcher reads the information aloud, while the potential participant can follow from their own copy with large fonts). Followed with MacCAT-CR assessment of capacity, then repeat of either of the consent processes. MMSE ≥ 19 **Adults with impaired capacity**	35	**Key findings:** participants improved their understanding scores after verbal re-explanation of consent information. There were no significant differences in level of understanding among those in the SSP versus the EWCP conditions at either trial, but we found the SSP took significantly less time to administer.
Rubright et al. [63], USA, A3	2010	RCT testing whether a memory and organisational aid improves AD patient performance on measures of capacity and competency to give informed consent.	Alzheimer’s disease centre	**Dementia:** patients with Alzheimer’s disease	**Enhanced informed consent with memory and organisational aid or standardised informed consent:** intervention group received the additional aid which summarised the key elements in the drug Z-298 informed consent form. It presented information in the same sequence and header titles as presented in the informed consent form. The text simplified important points from the consent form using language at a sixth grade reading level. All participants went through a capacity assessment.	80/112 (71%) of potential AD patients, 30/33 (91%) of cognitively normal older adults	**Key findings:** the intervention group was more likely to be judged competent than the control group (*χ*^2^ = 8.2, df = 1, *p* = 0.004) and had higher scores on MacCAT-CR measure of understanding (*z* = 2.86, *p* = 0.004). This RCT shows that a memory and organisational aid tailored to the distinctive cognitive patterns of AD patients can improve the ability of patients with very mild to early moderate AD to provide their own informed consent to enrol in an early-phase clinical trial. **Challenge:** this tool was designed specifically for AD patients and may not generalisable to other populations. Use of formal capacity assessments should not be treated with strict cut-off measurements. More challenging for high-risk trials. **Solutions:** for patients at an early phase of AD, capacity can be enhanced.
Ford et al. [86], USA, B2	2008	RCT to evaluate the effects of social support on comprehension and recall of consent form information in a study of Parkinson’s disease patients and their caregivers.	Medical centre	**Parkinson’s disease:** Parkinson’s disease patients (mean age 71 (SD 8.6) years) and their caregivers	**Enhanced informed consent:** in the social support group, patient-caregiver pair was asked to complete the consent form in the same room compared to the control group who completed the forms in separate rooms. **Adults with impaired capacity**	136/143 (95%)	**Key findings:** 1-week follow-up, no significant differences in Quality of Informed Consent (QuIC) scores between participants receiving the social support intervention and the control group. Regardless of the group allocation, participants scored approximately 50% of the QuIC questions. But the findings showed that comprehension of consent form information was increased through the social support intervention in a ‘real-world’ clinical setting. **Challenge:** initial comprehension was low and remained relatively consistent within the 1-month period. **Solutions:** informational support provided by family caregivers. Caregivers who scored high on correct QuIC were associated with patient participants who with high QuIC scores, e.g. understanding information.
Campbell et al. [94], South Africa, B3	2017	Case-control study exploring if using iterative learning improves participants’ understanding of the research study and predictors of better understanding of the study at the initial screening.	Psychiatric hospitals and clinics	**Mental health:** patients with psychosis/schizophrenia	**Enhanced informed consent** (with iterative learning): following explanation, University of California, San Diego Brief Assessment of Capacity to Consent Questionnaire (UBACC) is administrated, if the person achieves > 14.5 demonstrating capacity to consent; informed consent is provided. **Adults with impaired capacity**	1056 participants—528 matched cases and controls. (target was 181 pairs)	**Key findings:** before iterative learning, 55% of cases and 33% of controls were scoring lower than the cut-off point for study participation. After iterative learning, only 7% of cases and 3% of the controls were unable to consent to participate. Iterative learning process improved decisional capacity and understanding of the study in both cases and controls. This process is repeated after iterative learning. **Solutions:** the study recruiters play a significant role in managing the quality of the informed consent process.
Palmer et al. [62], USA, A3	2018	RCT to evaluate the efficacy of a multimedia-aided enhanced consent process incorporating corrective feedback, compared with routine consent, among individuals with mild-to-moderate Alzheimer’s disease and non-neuropsychiatric comparison (NC) subjects.	Unclear	**Dementia:** individuals with Alzheimer’s disease (mild to moderate)	**Enhanced informed consent:** the enhanced consent procedure expanded on routine consent by adding a more structured, iterative process and by incorporating multimedia tools into the consent presentation. Participants were randomised to routine consent and enhanced consent procedures for participating in a hypothetical RCT lower risk (FDA-approved medication) or a high risk (phase 2 immunotherapy). Assessment of capacity using MacCAT-CR. **Adults with impaired capacity**	248—134 control, 114 Alzheimer’s disease	**Key findings:** regardless of whether randomised to the lower or higher risk protocol type, participants who received the enhanced consent procedure did not demonstrate significantly better decisional capacity scores compared with those who received the routine consent procedure. Findings could be due to rapid forgetting.
Moser et al. [93], USA, B2	2006	Quasi-experimental, pre-post study to determine whether a brief intervention could improve decisional capacity in people with schizophrenia.	Unclear	**Mental health:** individuals with schizophrenia	**Enhanced informed consent:** a very brief (less than 30 min), semi-individualised intervention consisting of a computerised presentation of the hypothetical study information in a bulleted, simplified format, with one key point per slide. Participants viewed the presentation and read along as the examiner read aloud each slide. Following the educational remediation, the examiner reviewed with the participant all MacCAT- CR ‘understanding’ items for which the participant did not receive maximum credit. Participants received both a standardised intervention, individualised discussion, and corrective feedback regarding the aspects of the research protocol that they found confusing. The MacCAT-CR interview was then repeated to assess participants’ decisional capacity following the educational remediation. Finally, the examiner a briefer structured interview designed to assess the adequacy of participants’ understanding of the hypothetical study. **Adults with fluctuating capacity**	30 individuals with schizophrenia and 30 healthy comparison participants	**Key findings:** at follow-up, the schizophrenia group had improved significantly on understanding (*t* [27] = 2.85, *p* = .008) and was no longer significantly different from the comparison group on any of the four dimensions of decisional capacity on the MacCART-CR scale (*p* = .13–.33). Follow-up analyses also showed a significant effect of the intervention on a sub-set of the schizophrenia group who had performed most poorly at baseline, from a baseline mean of 18.0 (SD = 4.7) to 20.6 (SD = 4.9; *t* [7] = 2.59, *p* = .029). The effect size for this change is moderate in size (Cohen’s *d* = 0.6). Participants with schizophrenia earned significantly lower scores than those in the comparison group across multiple neuropsychological domains. **Challenge:** unable to determine what aspect of the intervention used was most helpful (active ingredients), as all participants in the schizophrenia group received both the standardised computer presentation and the individualised corrective feedback components. Had healthy comparisons, not other schizophrenia patients. **Solutions:** those individuals who initially lacked decisional capacity may benefit significantly from enhanced consent procedures. Further research is needed to unpick which components of the intervention are those causing the improvements.
Jeste et al. [92], USA, A3	2009	RCT to evaluate the effectiveness of a multimedia versus routine consent procedure (augmented with a 10-min control video presentation) to enhance understanding among adults with schizophrenia and healthy comparison subjects.	Outpatient clinics of an older mental health service	**Mental health:** older patients with schizophrenia	**Enhanced informed consent:** multimedia consent; the research assistant (RA) provided participants with the printed consent form. Subjects watched a DVD that explained the protocol. Multiple representation and contiguity principles were present throughout the DVD by presenting consent-relevant information through a narrator explaining key points, with simultaneous visual presentation using graphics, pictures, animations, and summary text (bullet-pointed). Subjects were encouraged to ask the RA to stop the DVD and repeat any segments that were unclear. Participants were encouraged to discuss and clarify issues with the RAs. Such discussion is important for multimedia consent aids to aid person-to-person interaction not to substitute. **Adults with impaired capacity**	128 middle-aged and older persons with schizophrenia and 60 healthy comparison subjects	**Key findings:** outpatients with schizophrenia provided with a multimedia-aided consent procedure demonstrated better comprehension of a research protocol and were more likely to be categorised as being capable of consent under three different standards examined, compared with those presented with an enhanced routine consent procedure. MacCAT-CR understanding subscale for outpatients with schizophrenia trial 1 (*d* = 0.6384, *p* = 0.0055, 95% CI 0.54, 0.74), trial 2 (*d* = 0.6108, *p* = 0.0237, 95% CI 0.52, 0.71), trial 3 (*d* = 0.6117, *p* = 0.0169, 95% CI 0.52, 0.70), UBACC total (*d* = 0.6795, *p* = 0.0003, 95% CI 0.59, 0.77). There were few differences between the two (routine and multimedia) consent conditions among the healthy controls. **Challenge:** comprehension can be improved with simple procedures such as corrective feedback/iterative learning. Hence, considering the additional resources, whether a full-multimedia presentation is needed is questionable. **Solutions:** multimedia consent procedures may be a valuable consent aid that should be considered for use when enrolling participants at risk for impaired decisional capacity, particularly for complex and/or high-risk research protocols.
Harmell et al. [91], USA, A3	2012	RCT to evaluate the preliminary feasibility and potential effectiveness of a web-media approach to consent, i.e. to determine whether development of such web-media based tools warrants further pursuit.	Unclear	**Mental health:** patients with schizophrenia	**Enhanced informed consent:** participants allocated to web-media consent reviewed the study information on a web-media prototype, which involved video clips, static images/graphics, and bullet-pointed text to explain the consent form. The printed consent form was presented on the screen in sections covering: e.g. study introduction, a timeline with study visits, description of procedures, risks/discomforts, possible benefits, action if injured, and voluntary participation. The tool included questions with corrective feedback after each section to check understanding. Participants could ask questions at any point and replay the presentation. **Adults with impaired capacity**	19 patients with schizophrenia and 16 normal comparison	**Key findings:** relative to those receiving the routine consent procedure, those receiving the web-media consent evidenced better UCSD Brief Assessment of Capacity to Consent (UBACC) scores, *U* = 19, *z* = − 2.15, *p* = 0.03 (*d* = 0.94; ‘large’ effect size). Participants rated the quality of the enhanced consent procedure as ‘better’, and no participant reported worse experience. **Challenge:** increased length of administration and that computer-based approaches may intimidate people with less computer literacy. **Solutions:** incorporating audio-visual materials on a computer/web platform to enable a more interactive and flexible presentation is feasible and more acceptable than presentation on a DVD. Such presentation may enable researchers to capitalise on the benefits of audio-visual learning, while circumventing the limitations of a DVD presentation.
Sudore et al. [82], USA, B3	2006	Observational nested in a trial of two advance directives to describe a modified research consent process, and determine whether literacy and demographic characteristics are associated with understanding consent information.	Hospital	**Geriatric care/older patients:** ethnically diverse subjects, aged ≥ 50, consenting for a trial to improve the forms used for advance directives	**Enhanced informed consent:** a modified consent process—consent form (written at a sixth grade level) read to participants, combined with 7 comprehension questions and targeted education, repeated until comprehension achieved (teach-to-goal).	329 potential participants. Twenty participants refused to participate, 39 excluded due to scheduling issues, 61 did not meet the eligibility criteria, and data were missing for 1 participant, leaving 208 participants. 208/329 (63.2%)	**Key findings:** despite significant consent modifications (improving readability of the consent form, having bilingual research assistants read the consent form to participants, and allowing time for discussion), few participants (28%) had complete comprehension and required only 1 pass through the consent process. However, further use of a teach-to-goal strategy was successful in achieving complete comprehension in 98% of all participants who engaged in the consent process, including those with literacy or language barriers. **Challenge:** the comprehension statements could have addressed all of the required elements of informed consent, making our results more generalisable. **Solutions:** for the majority of these participants, little additional education was required.
Rikkert et al. [81], Netherlands, C1	1997	Pre- and post-test study of a step-wise consent process to determine the effects of research experience on the capacity to consent.	Hospital	**Geriatric care:** geriatric patients	**Step-wise consent: (**1) **Eligibility screening**. (2) **Research experience** was given by a try-out period of a week. Verbal and written information about the try-out was given to all eligible subjects (*n* = 78). For 40% of potential subjects, the family members were willing to accompany them when they received information. 70 subjects (90%) provided **verbal consent** to participate in the try-out. (3) **After the try-out, written informed consent was requested.** The verbal information was repeated. Assessment of the capacity to consent was conducted before and after experiencing research by testing comprehension and ability to weigh risks and inconveniences. **Adults with impaired capacity**	53/78 (68%)	**Key findings:** the try-out effect on the comprehension scores could be tested in 53 subjects who provided written informed consent. Initially, the subjects answered only 5.0 of the ten questions correctly, this number increased to 7.0 after the try-out. This step-wise consent procedure resulted in a participation rate of 68% (53/78) of all eligible subjects. During the try-out, seven subjects withdrew consent. Ten subjects refused informed consent to continue research following the try-out. **Solutions:** research experience seems to improve the capacity to consent in demented and depressed subjects as well as in subjects without psychogeriatric illnesses.
Processes to enable adults lacking capacity to participate in research							
Advance and process consent							
Olazaran et al. [64], Spain, C1	2012	Research protocol to provide an overview of the clinical research protocol of the ACRSF (Alzheimer Center Reina Sofia Foundation), to analyse the adequacy of the assessment instruments, and to report on changes to the protocol.	ACRSF research centre	**Dementia:** patients with Alzheimer’s or other dementia and their relatives who agreed to receive treatment from the ACRSF (MMSE 6.7 (6.1)—nursing home to 9.1 (7.6)—day-care centre)	**Advance consent upon arrival to the facility; legal representative to provide informed consent**: upon admission, one or two ACRSF research physicians introduced the patient and family caregivers to the ACRSF research programme and invited the legal representative to sign consent to participate. The consent form included separate boxes for agreement to specifically participate in the clinical, biochemical, genetic, MRI, and neuropathological programmes. **Adults with impaired or diminished capacity**	180 (80% of the total)	Informed consent was obtained from 180 patients. Those patients represented approximately 80% of all patients admitted at the ACRSF during that period. Demographics: the two groups of patients studied were old (outpatients) or very old (inpatients), had very low educational achievement, and were predominantly women. **Solutions:** multidisciplinary action.
Gysels et al. [45], UK, C1	2013	Consultation workshop and TEC to present the processes and outcomes of a workshop and consensus exercise on agreed best practice to accommodate ethical issues in research on palliative care.	N/A	**Palliative care**	**Advance consent, process consent** **Adults with impaired or diminished capacity**	28	**Key findings:** 16 recommendations generated. The recommendations on obtaining and maintaining consent from patients and families were the most contentious. **Challenge:** fluctuating capacity, time, risks involved in participating. **Solutions:** informing all the patients/relatives on admission that the facility conducts research, minimises gatekeeping, and identifies people interested in research participation. The level of detail on the information sheets should be proportional to burden and risks. Advance consent (early informed consent when the patient still has capacity) for all research, not just CTIMPs. Contemporaneous assent should also be obtained for all trials. Consent should be a continuous process. Consent process < 24 h after approach with clear justification to avoid coercion.
Cowdell et al. [70], UK, B3	2008	Ethnographic study exploring strategies that were used to enable older people with dementia to become actively engaged in research with them.	Hospital	**Dementia:** inpatients ≥ 65, with a dementia diagnosis at an advanced stage of the illness	**Process consent:** verbal or behavioural consent was taken from participants at the beginning of every period of observation to ensure they were willing to continue. This consent was negotiated between the person with dementia, the researcher, the staff on duty, and on occasion the next of kin. Participants were observed for any signs that they might wish to withdraw. For the interview part of the study, participants were asked to sign a consent form.	125 h of observations and interviews (*n* unclear) in an inpatient setting	**Key findings:** actively engaging older people with dementia even at advanced stages (e.g. instead of having a pre-defined MMSE cut-off) in research is possible. Researchers need to apply ethical principles and rules sensitively and flexibly. **Challenge:** ensuring the informed nature of the consent, without having a formal capacity assessment.
Dunning et al. [46], Australia, B3	2012	Individual semi-structured interviews, field notes, philosophical framework to discuss the ethical and methodological issues encountered when undertaking research to develop guidelines for managing diabetes at the EoL.	Participants’ homes	**Palliative care:** semi-structured interviews with 14 men and women with diabetes and 10 spouses of the 14 participants	**Process consent:** involved asking participants whether they wanted to continue the conversation during the interview. Process consent was used when a participant became physically or emotionally distressed (in addition to **informed consent**). **Adults with impaired capacity**	Not known	**Key findings:** qualitative research deemed to be the most effective data collection method. **Solutions:** attention to protecting participants’ privacy, ensuring they can give informed consent, being aware of their physical and mental state, and periodically checking their willingness to continue participating during interviews and focus groups, is essential.
Hughes et al. [65], UK, C1	2015	Qualitative consultation aiming to develop an approach within the guidance of the Mental Capacity Act (2005) to meaningfully include people diagnosed with dementia in research endeavours.	Integrated dementia day care services	**Dementia:** people with a dementia diagnosis residing at residential care homes	**Process consent** **Adults with impaired capacity**	8/9 (one declined to participate due to unexpected housing issues)	**Consent process:** first consent of participating service leads to allow identification of eligible patients. Then, the tested consent process was implemented, and patients’ consent was assessed and recorded. Consent reliability—not having a one-off consent and renewing consent at every encounter. **Challenge:** participants lost track of the purpose of the research. Important to attend to non-verbal cues. Gatekeeping from relatives and switch of decision-maker from patient to relative based on the context of the decision. **Solutions:** researchers should be trained in reflexive assessment of consent using verbal and behavioural cues. Initial approach to participate could be by a trained service user consultant to balance the power dynamics. Researchers should involve all parties not limit to, for example, family caregiver.
Carey et al. [95], Ireland, B3	2017	Qualitative study to explore theoretical underpinnings of intellectual disability research, and to discuss the ethical and methodological considerations in recruiting and obtaining informed consent from adults with intellectual disabilities.	Intellectual disability service/participants’ preferred place and time	**Mental health:** adults with intellectual disability	**Process consent** **Adults with impaired capacity**	12/14	**Consent process:** ongoing informed consent—detail information session, option to have a support person present. Written consent. Six participants also wanted their consent to be recorded. Non-verbal cues were considered while assessing ongoing consent. **Solution:** the structure of these meetings facilitated discussions about the nature of the study. **Key findings:** making reasonable accommodations to support decision-making, making space for the development of empathic relationships with both the potential participants and with the structures and service supports.
Deferred consent							
Adamis et al. [100], UK, B3	2010	Prospective cohort to assess serum IGF-I in patients with delirium and the way in which results altered when including patients with delirium who lacked capacity.	Elderly care unit	**Delirium:** patients 70 years or more with the presence of delirium using Confusion Assessment Method–Fluctuating capacity	**Deferred consent:** those who lacked capacity were entered **(deferred)** to study and their capacity were **re-assessed to see if they gained capacity** or **proxy assent** was obtained. **Adults with diminished capacity**	164/233 (70%). 13/23 recruited lacked capacity and 151/210 recruited with capacity.	**Key findings:** the inclusion of the more incapacitated subjects allowed a significant finding (lower serum IGF-I in prevalent delirium cases). Strengthened the evidence that IGF-I has a role to play in the pathophysiology of delirium. **Solution:** informal approach to capacity may allow for more representative results of the study population.
Honarmand et al. [104], Canada, B3	2018	Prospective, pilot study to describe the feasibility of the deferred consent model in a low-risk, observational study of critically ill patients (Prognostic Value of Elevated Troponins in Critical Illness Study [PRO-TROPICS]) and to determine the factors associated with consent procurement.	Intensive care units at three study sites across Canada	**Intensive care:** critically ill patients	**Deferred consent**: patients are enrolled to the study and then themselves or their surrogate decision-maker is approached for consent. Consent can be provided to ongoing study participation, use of data collected so far, or no consent for data to be used. **Adults with diminished capacity**	214/267 (80%)	**Key findings:** deferred consent model was feasible with 80% consent rate. Of 53 persons declining consent, 37 (70%) agreed to the use of the data collected to that point. One patient withdrew consent after it was provided by a proxy decision-maker. But, patients unlikely to recover were excluded. Consent rate did not differ based on who (patient/surrogate) was consenting. **Challenge:** exclusion of patients who might not recover/die and exclusion of patients who die early or cannot provide consent within the study timeframe lead to selection bias, reduced statistical power, and decreased external validity.
Consultee advice							
Black et al. [60], USA, C1	2007	Methodological paper focusing on three aspects of the consent process for dementia research: (1) providing information, (2) assessing understanding and capacity to consent, and (3) obtaining assent and informed consent. For each aspect, the differences between drug and non-drug studies in CDRS examined.	Six parent dementia studies	**Dementia**	**Informed consent and/or personal consultee advice (dual consent)**	Researchers (*n* = 11), patients, and their personal consultees from six dementia studies—46 consent process observations	**Key findings:** study revealed wide variability in how informed consent was obtained. (1) Consents forms were provided to the patients and personal consultees prior to enrolment visits and often served as a guide for consent discussions; (2) consent discussions were more consistent and comprehensive for drug studies than non-drug studies; (3) study procedure explanations dominated the discussions, whereas the rights of research subjects were mentioned less frequently; (4) assessments of affected individuals’ understanding and capacity to consent occurred in a minority of cases but were more likely to occur on drug studies; (5) personal consultee advice was sought more often using an implicit rather than an explicit approach; (6) dual consent by both the affected individual and surrogate decision-maker was most common on both drug and non-drug studies; (7) personal consultees often played a major role in facilitating the consent process. **Solutions:** describing the purpose of the study; discuss the individual’s rights in detail; involving the personal consultees; explicitly ask the potential participants for their involvement; use a standardised way of assessing capacity; explain why a personal consultee advice is needed.
Agarwal et al. [69], UK, C1	1996	Observational study examining the relevance of the Law Commission recommendations in accessing informed consent from early dementia patients and their carers subjected to a double-blind, placebo-controlled trial of a potentially therapeutic agent.	Unclear	**Dementia:** patients and carers	**Personal consultee advice:** two questionnaires (for patients and their carers) were designed to examine whether subjects fulfilled the criteria for a ‘cognitive’ or ‘function’ test of capacity to *consent to participate in a research study.* This was an attempt to establish whether consent was a ‘true choice’. **Adults with impaired capacity**	15 patients and carers	**Key findings:** a single legal ‘test’, with stringent criteria, applied across the board for all treatment and research conditions, may impede future research activity as none of the subjects fulfilled the criteria for determining whether participation was a true choice. **Challenge:** implied consent (opt-out) could lead to exploitation of vulnerable patients. **Solutions:** the role and involvement of carers in the decision-making process need to be considered. Provided that they are acting in the patient’s best interests, that the patient has not actively expressed a desire not to participate, and that the research is potentially *therapeutic*, with the research drug having negligible side effects, this is unlikely to violate his fundamental rights.
Gainotti et al. [68], Italy, A3 (methods paper reporting RCT [79])	2010	Methodological paper hypothesising that the requirement that informed consent for an incapacitated subject’s participation to research be given by a legal representative appointed by the courts slows down the recruitment process in research thus complicating the conduction of dementia research in Italy.	Outpatient clinic	**Dementia:** outpatients seeking medical advice for cognitive complaints	**Legally appointed consultee advice:** the procedure to obtain informed consent in the study was quite elaborated. First, subject’s competence was evaluated by means of the MMSE. If the subject’s score was ≥ 20, then he/she underwent four additional neuropsychological tests. If the subject’s score to the four tests was higher than the established cut-offs, the subject was deemed able to give informed consent. If the subject’s MMSE score was < 20, adjusted for age and education, or if the subject’s score to the other four tests was lower than the established cut-offs, the subject was deemed unable to give informed consent and consent had to be given by a legally authorised representative. **Adults with impaired capacity**	78/172 (46.2%) required legal consultee appointment, 55/78 (70.5%) received appointment	**Key findings:** the requirement that the legal representative be appointed by the courts may impede a subject’s participation in research. It may cause embarrassment and conflicts among family members, it may have been received as a bureaucratic and burdensome task, and relatives may be reluctant to go to court due to stigma. This results in only a privileged selection of patients being involved in the studies. **Solutions:** Removal of legal procedure for the involvement of consultees or fastening the legal processes and reducing burden.
Adamis et al. [99], UK, A3	2005	RCT to investigate whether different methods of obtaining informed consent affected recruitment to a study of delirium in older, medically ill hospital inpatients.	Acute medical service for older people at a hospital	**Delirium:** patients 70 years or older admitted to the unit within 3 days of hospital admission	**Informed consent or proxy assent:** both groups of patients were given routine same information (verbally and written). After a formal capacity assessment, assent was sought from a proxy (if available) if patient lacked capacity in group A, whereas in group B, an informal capacity assessment took place and those individuals who deemed to lack capacity were excluded.	57 assessed in group A (43.8%), 25/57 (43.9%) entered the study. 73 assessed in group B (56.2%), 54/73 (74%) entered the study. 20 patients in each group were recorded as ‘case note delirium’.	**Key findings:** implementing best ethical practice by a formal assessment of capacity to consent to a research project in an acute medical ward will lead to a considerable reduction in the proportion entering the study. A stringent assessment of capacity may lead to reduced generalisability of the study findings. In turn, this undermines the ethical justification of the study. Of the 20 patients in each of the initial randomised groups (A and B) with case note delirium, 7 (35%) in group A and 16 (80%) in group B (*p* = 0.004) entered the study (*χ*^2^ = 8.29, df = 1; *p* = 0.004). **Challenge:** researcher assessing the capacity was not blinded to group allocation. Many potential participants with delirium do not have formal capacity to consent. In this study, including almost all prospective patients admitted to an elderly care unit, 40% lacked capacity to give consent to this research when judged formally. The process of formal testing of capacity might have resulted in bias by inducing higher rates of declining to give consent. **Solutions:** the consent rate may be greater if a step-wise approach to consent during participation is used. In this approach, called ‘experienced consent’, verbal consent is accepted initially, and after the subject has experienced the project, written consent is sought.
Morán-Sánchez et al. [90], Spain, C1	2016	Cross-sectional survey to evaluate the association between capacity to consent to research and the more prevalent psychiatric disorders, and to characterise factors associated with impairments in capacity across diagnostic groups.	Mental health care	**Mental health:** psychiatric patients	**Informed consent and legal guardian consent:** capacity was assessed using MMSE and MacCAT-CR. **Adults with impaired capacity**	139/235 (59%)	**Consent process:** informed consent patients with capacity or from legal guardian if lacked capacity. **Capacity:** the level of understanding needed to provide meaningful consent to participate in this minimal-risk protocol was much lower than that required for a complex or higher-risk clinical trial, such as that described in the hypothetical protocol used to evaluate capacity in the study. MacCAT-CR used to assess capacity. **Key findings:** no subject was excluded because of a lack of capacity. 31% of the participants lacked decisional capacity to provide informed consent. Those lacking capacity were more likely to be older, with severe illness over a longer time. The number of psychiatric admissions was higher in the incapacitated group. They were more likely to have a psychotic or mood disorder and to score lower on the MMSE. **Solutions:** cognition must be considered in capacity assessment. Understanding can be improved through enhanced consent procedures.
Thomalla et al. [87], Germany, A3	2017	RCT (baseline data only). Aim to determine if the manner of consent, i.e. informed consent by the participant or by proxy decision-maker, affected clinical characteristics of samples of acute stroke patients enrolled in clinical trials.	Hospital	**Stroke:** stroke patients	**Informed consent (written or oral) by patient, personal or legal proxy, consensus between the investigator and an independent clinician:** six options give including written or oral consent by the patient, legal guardian consent, NoK consent, investigator’s decision (followed with consent from NoK as soon as possible). **Adults with diminished capacity**	1005/1039 (ongoing trial)	**Key findings:** in 646 (64%) patients, informed consent was given by the patients; in 359 (36%), consent was by a proxy. The relative frequency of the informed consent type used varied among countries (*p* < 0.001). In this analysis of baseline data of the first 1005 patients enrolled in the WAKE-UP trial, about 1 in 3 patients were enrolled by proxy consent. In these cases, consent was provided by the legal guardian, by next of kin, by an independent consultant, or by the investigator based on an emergency clause. **Challenge:** limited guidance around regulation of clinical research in patients lacking capacity to give informed consent, and the consequence of different approaches for informed consent used in different stroke trials, among countries or trial sites.
Kim et al. [66], USA, C1	2011a	To assess the extent to which persons with Alzheimer’s disease (AD) retain their capacity to appoint a research proxy.	Interview study	**Dementia:** people with Alzheimer’s disease (MMSE 18–23)	**Proxy consent** **Adults with diminished or impaired capacity**	700	**Key findings:** successful recruitment had the highest proportion (46.3%) of participants with MMSE score range of 18–23. Reliability of the judges’ determination of capacity was high. 61.7% of participants had the capacity to appoint a research proxy, 41.4% had capacity to consent to the drug RCT, and 15.6% had the capacity to consent to the neurosurgical RCT. A substantial proportion of AD subjects thought incapable of consenting to lower or to higher risk studies had capacity to appoint a research proxy. **Solutions:** providing for an appointed proxy even after the onset of AD may help address key ethical challenges to AD research. Appointing a proxy is advocated early in the disease trajectory.
Warren et al. [47], USA, C1	1986	Qualitative interviews to examine the bases on which the proxies made their decision, to identify characteristics that distinguished proxies who refused consent from those who gave consent, and to determine reasons for refusal.	Nursing home	**Palliative care:** nursing home residents’ (those who were > 65 years old) proxies	**Proxy/surrogate consent** **Adults with diminished capacity**	90/168 (54%)	**Key findings:** 54% (*n* = 90) of proxies approached consented to patients’ participation. 60% of proxies consulted other people about their decision; 27% consulted a clinician about advising participation (or not). No significant difference in the frequency of consent among those who decided alone, those who consulted others, and those who consulted a clinician. Most proxies were not opposed to research in general, but as beliefs and perceptions about research became more relevant to their own family members, their support for research, even in the abstract, declined. 96% thought research in general was important for medical care; 87% agreed for research to be undertaken in hospital; 83% thought that elderly people should participate in research; only 66% thought that research should be conducted in nursing homes. **Solutions:** broad educational effort to increase awareness of the relevance of research to the health of older people across care settings. To discuss participation in research with patients while they are competent and to include potential proxies in these early discussions.
Karlawish et al. [67], USA, C1	2008	Companion study to an RCT placebo controlled of a potential Alzheimer’s disease treatment (drug) study to examine the views of Alzheimer’s disease patients and their study partners on the ethics of proxy consent for clinical research.	Universities	**Dementia:** patients with mild-to-moderate AD (MMSE 12 to 16), and their study partners (spouse or adult child)	**Proxy consent** **Adults with diminished capacity**	59/73 (81%) patients, 60/75 (80%) study partners	**Key findings:** study partners of patients judged incapable of giving informed consent reported the same degree of patient involvement in the decision to enrol as the study partners of patients capable of giving informed consent. Most study partners and patients supported proxy consent for the clinical trial, and nearly all patients chose their study partner and their proxy. Study partners generally made research enrolment aligned with maximising the patient’s well-being. **Solution:** pursue a process of shared decision-making between patient and study partner to recruit patients with impaired capacity.
Smith et al. [107], Canada, C1	2013	Qualitative study with interactive focus groups to present strategies that may optimise the process of obtaining informed consent from substitute decision-makers for participation of critically ill patients in trials.	Intensive care unit	**Intensive care:** research coordinators working with critically ill patients	**Surrogate consent: informed consent from substitute decision-makers** of critically ill patients **Adults with diminished capacity**	71	**Key findings/solutions:** (1) brand the trial with key messages, (2) train the local personnel, (3) promote a culture of research, (4) be familiar with patient family dynamics, (5) involve bedside staff—make them aware that you are interested in recruiting their patient, (6) introduce the idea of research in a professional manner—explain why you are approaching the surrogate decision-maker, (7) present the facts about the research problem and outcomes, (8) convey risks and benefits transparently, (9) describe alternatives to participation and support the consent decision, (10) explain all research-related activities, (11) Document the consent process, (12) provide thanks and ongoing study updates to all stakeholders, and (13) follow-up with the patient to ensure ongoing consent. Strategies reinforce requirements outlined in existing legislations and additional process to enhance the integrity of the consent process.
Bolcic-Jankovic et al. [105], USA, C1	2014	Cross-sectional quantitative survey on (a) the determinants of confidence in a surrogate’s ability to make a decision for the patient, (b) the difference between surrogates’ and patients’ confidence, (c) if greater confidence increases agreement between the surrogate’s and patient’s response.	Intensive care unit	**Intensive care:** patients who required ICU and who had potential to regain capacity after recovery, and surrogate decision-makers.	**Surrogate consent** **Adults with diminished capacity**	445 surrogates, 214 patients	**Consent process:** surrogate consent obtained during the patient’s ICU admission. **Key findings:** the research funder, knowledge about the study, and discussing with a trusted person were associated with surrogates’ confidence in advocating participation and attitudes towards research. Patients’ confidence in their surrogates’ decision was associated with a previous discussion about research participation (*p* < .001). Confident surrogates responded in agreement with patients’ wishes (80%). Most surrogates wanted to represent the person’s wishes. **Solution:** Early discussions between the proxy and patient about research participation.
Fowell et al. [48], UK, A3	2006	Randomised crossover trial to explore the feasibility of two designs of consent for dying patients: randomised consent (aka Zelen’s design) and cluster consent to see which design is more effective for trials in palliative care.	One oncology and one palliative care unit	**Palliative care/cancer:** patients with a terminal cancer diagnosis who were on an integrated care pathway for the dying	**Cluster consent**: consent at unit level for a group from a ‘cluster guardian’, and ‘cluster gatekeeper’ responsible for individual patient approach. Both guardian and gatekeeper must give written agreement for their cluster to participate in the trial. **Randomised consent (Zelen’s design)**: seeks informed consent after randomisation but only if the patient is to receive the experimental treatment. **Adults with declining, impaired, or diminished capacity**	20/50 (60%)	**Key findings:** the initial request to abstract data was identical in both designs and significantly fewer Zelen patients in the larger unit consented to this. Zelen’s design reduces the burden of seeking consent for treatment allocation but did not improve recruitment. **Solutions:** cluster randomisation runs in the background, reducing burden on the patient, carer, and clinician. Consent to treatment allocation is at the unit level with individual patient consent for access to confidential medical data. This study illustrates how cluster randomisation exploits these natural advantages, particularly with dying patients.
Levine et al. [106], USA, C1	2017	Expert consultation—electronic survey followed with Delphi rounds aiming to establish a broader consensus on the barriers to emergency care research globally and proposes a comprehensive array of new recommendations to overcome these barriers.	Global emergency medicine covering	**Intensive care/emergency medicine:** experts in global emergency medicine	**Community consent** **Adults with diminished capacity**	80	**Solutions/suggestions:** streamline data collection, identifying alternatives to local IRB approval and the use of community consent when appropriate where the individuals can choose to opt-out of the study later on. Key findings were divided into four categories. (1) Limited availability of research training. (2) Logistical issues and lack of data collection standardisation. (3) Ethical barriers regarding conducting research in low-income countries. (4) Dearth of funding for global emergency research. **Key findings:** need for ethical curriculums including important topics related to the ethics of acute care research internationally such as consent, loss to follow-up, coercion, and undue influence for enrolment was highlighted.
Boxall et al. [88], UK, B3	2016	Qualitative focus groups and interviews exploring the barriers to recruiting stroke patients to clinical trials from the viewpoint of experienced nurse researchers. Secondary aims included exploring the factors affecting the recruitment of stroke patients, explore the main themes that influence recruitment, and determine if stroke research faces unique recruitment issues.	Hospitals	**Stroke:** stroke research nurses	**Surrogate consent, paramedic/early-on-scene consent, exception/delayed consent** **Adults with diminished capacity**	12	**Challenges:** restrictive inclusion/exclusion criteria, physician endorsement, and lack of clinical equipoise. Impairments affecting capacity to consent—lack of validated tools to help assess capacity. Acute time frame to recruit, paternalism of (especially less-experienced) nurse researchers. **Solutions:** engaging caregivers and, if possible, using surrogate consent. Finding a balance between giving patients the opportunity yet not coercing them. **Consent process (suggestions):** ■ Traditional, in-hospital consent with a clinician and written information. ■ Paramedics/early on-scene consent. ■ Exception or delayed consent. ■ Surrogate (relative, legal representative, or independent physician) consent. ■ Short or abbreviated written information. ■ Use of pictorial information sheets or videos to explain a study ■ Telephone or video consent.

### Quality appraisal

Overall, the quality of the included articles was medium to high. Most quantitative (95.8%, *n* = 71) and qualitative studies (88.9%, *n* = 17) were assessed as medium or high quality (see Additional file 2: Table S5 quantitative studies and Table S6 qualitative studies). The proportion of high-quality studies included was consistent across the three main areas (56.8% ‘innovating research methods’, 56.6% ‘applying consent processes’, and 59.1% ‘public attitudes’). However, in the area of ‘public attitudes on involving adults lacking capacity’, 9% were assessed as low quality, compared with 0% in ‘innovating research methods’ and 2% in ‘applying consent processes’. This reflected in part the methodological nature of the studies and poorer fit with the Qualsyst item criteria. The included studies were mainly descriptive (*n* = 36) categorised as ‘non-experimental, longitudinal, cohort, matched pairs, or cross-sectional, sound qualitative, or analytical studies’, with few experimental (*n* = 20) or quasi-experimental designs (*n* = 3) (see Additional file 3: Table S7).

### Innovating research methods to recruit adults across the capacity spectrum

Thirty-seven studies were categorised as innovating research methods (Table 3). Studies focused on participation in research involving individuals with cancer/receiving palliative care (*n* = 6), dementia (*n* = 13), geriatric care (all settings) (*n* = 2), delirium and mental health services (*n* = 7), or intensive care (*n* = 5). While numerous studies used standardised capacity assessment tools, existing tools were often regarded as time-consuming, and administration of a formal capacity assessment reduced recruitment, for example in an observational study involving patients with delirium [99]. Formal capacity assessment was considered of little value unless aligned to the decisional requirements for study participation, notably the risks and the potential direct or indirect benefits of participation. Overall, studies incorporated multiple components of the processes of consent. These were tailored to individuals’ level of capacity from mild to moderate impairment with a focus on enhancing informed consent, through to lacking capacity requiring involvement of a consultee. For example, in populations such as psychiatric or stroke patients, where participants experienced varying levels of impaired capacity, studies incorporated processes of enhanced informed consent and consultee involvement [87, 90]. In both studies, consultee advice was sought for a third of participants (30.6% and 35.7%, respectively). The innovations broadly mapped onto two sub-categories of ‘maximise individuals’ autonomy and decisional capacity in the consent process’ and ‘processes of consent to enable adults across the capacity spectrum to participate in research’.

#### Maximise individuals’ autonomy and decisional capacity in the consent process

Fifteen studies aimed to examine ways to enhance the informed consent process to maximise decisional capacity for adults with mild/moderate capacity or fluctuating capacity (see Table 3). Clinical trials required applicable methods to facilitate understanding of complex procedures, e.g. randomisation and clinical equipoise. For populations with mild/moderate dementia, and other neuropsychological disorders such as Parkinson’s disease, studies aimed to enhance various facets of cognition. This involved in the informed consent, for example, enhancing decisional capacity, understanding [90], reasoning, comprehension, and recall of information. Across patient groups, key challenges to enabling participation in the informed consent process were addressing concerns about causing distress for the person from receiving and considering study information [43], and enabling understanding and recall of the information provided [59, 103]. Moreover, in ensuring an informed consent rather than participation based on trust in the clinician and the study proposal considered a recommendation to take part [103]. A study observing consent process for people with dementia (*n* = 46) and their surrogate decision-maker (e.g. spouse) across six trials (drug and non-drug) showed approaches to facilitating the consent process and wide variation [60]. Importantly, the consent process involved the person with dementia and their surrogate, with the surrogate playing a major role in facilitating the consent process. Use of a dual consent was common, but assent from the person was often implicitly implied not explicitly asked. The study revealed wide variability in the conduct of the consent process with higher risk drug trials generally more comprehensive in giving study information and assessing understanding compared to the non-drug trials. The authors make recommendations on using a dual consent process and standardising the information provided to encompass both study procedures and participants’ rights to, for example, withdrawal.

Successful enhanced informed consent processes often included providing information in more than one format (generally verbal and written) [61], improving understanding by using novel techniques (e.g. simplified storybook, video) [91–93], and tailoring the process of study approach to a person’s psychological and physical health status [43]. In studies involving patients with schizophrenia [91, 93, 94] or dementia [62, 63], using a combination of multimedia techniques and flexibility to repeat aspects of the study information significantly improved individuals’ understanding about the study, and to give an informed consent. Studies using enhanced informed consent processes reported generally high consent rates for eligible participants ranging from 68.0% (*n* = 53/78) in an elderly patient population [81] (mean age 80.1 years) to 95.1% (*n* = 136/143) in patients with Parkinson’s disease (mean age 71 years) [86]. The addition of social support from family members enhanced older individuals’ decision-making capacity. For example, a study with hospitalised older cancer patients (MMSE mean 24.0, SD 4.1) achieved 74% (37/50) recruitment rate by formal involvement of family members including presence during the consent process, asking their views, encouraging patients to discuss with the family members, and taking their concerns into account [44]. In an RCT (*n* = 136) on the effect of social support on the consent process for patients with Parkinson’s disease (mean age 71 years, SD 8.6), the presence of a family member compared to the patient alone showed effect on enhancing comprehension and recall of the study information at 1 week (*p* = 0.012) and 1 month (*p* = 0.040) [86].

However, the evidence for using multiple or iterative techniques to enhance individuals’ understanding and reasoning was mixed. Variation was related to the context and the processes used. Using techniques of iterative learning by, for example, quizzing potential participants’ understanding of the study and enhancing how information was provided [86, 92] enabled study engagement for individuals with lower levels of health literacy from, for example, lower socioeconomic backgrounds [82] or low- to middle-income countries (LMICs) [94]. However, findings were mixed for studies recruiting adults with dementia. RCTs showed enhanced understanding (*F* [1, 29] = 7.17, *p* = 0.012) using a PowerPoint presentation, combined with verbal consent and verbal re-explanation for participants with mild Alzheimer’s disease (MMSE ≥ 19) (*n* = 53) [61], and using a simplified memory and organisational aid to improve understanding (*χ*^2^ = 8.2, df = 1, *p* = 0.004) (*n* = 110) [63]. Conversely, a RCT (*n* = 114) using multimedia and iterative learning for participants with mild to moderate Alzheimer’s disease (MMSE 20.9 (SD 3.9) and 22.5 (SD 3.4), by treatment arm) did not detect a significant effect on decisional capacity (*χ*^2^(3) = 2.63, *p* = 0.453) [62].

#### Processes of consent to enable recruitment of adults across the capacity spectrum

Twenty-one studies explored research methods in the consent process for adults across the capacity spectrum. Advance consent was advocated in two studies for participants with the anticipated loss of capacity associated with a progressive condition, e.g. dementia [45, 64]. Advance consent involved early informed consent when the person had capacity, for example, on admission to a clinical facility, with consent upheld at the point of loss of capacity [64]. An area of contention was the requirement (or not) in all research studies (Clinical Trial of an Investigational Medicinal Product (CTIMP) and non-CTIMP) for the person to nominate a consultee for contemporaneous advice on continued participation in the study [45]. Best practice was conducting the advance consent process with the person and the consultee (e.g. a family member), to discuss the person’s preference for continued participation should they lose capacity, and the role of the consultee on contemporaneous advice aligned to the person’s wishes and the context [45, 64]. This strategy may minimise potential gatekeeping about continued participation with disease progression [45]. Process consent was proposed for studies with multiple time points that involved individuals able to understand and appraise information in the moment, but with difficulty retaining and recalling study-related information in the future, for example, individuals with mild/moderate dementia, palliative care, and mental health populations [46, 65, 95]. Process consent involved continuous monitoring of the validity of the informed consent provided at the beginning of the study, for example, verbal confirmation of consent at each time point with the formality of the process consent proportionate to the risks involved in participation.

Two studies reported the use of deferred consent in circumstances of fluctuating capacity with participants anticipated to regain capacity with reversibility of the underlying cause, for example, an infection [100, 104]. Patients who were unlikely to recover or die were typically excluded. Patients entered the study without their prior consent, with consent deferred until they regained capacity and/or a consultee was approached. These types of studies typically involved low-risk observational procedures conducted with, for example, older patients with delirium [100] or in the ICU [104]. Using deferred consent enabled the inclusion of participants at the acute point of their illness spectrum. Deferred consent appeared acceptable in the ICU study with an 80.1% (*n* = 214/267) consent rate [104], and sensitivity analysis of the delirium study indicated that excluding ALC would have compromised the detection of statistically significant findings [100].

The main process of enabling adults lacking capacity to participate in research was seeking advice from a consultee about study participation. Most studies (*n* = 11) explored the involvement of consultees across research studies in geriatric/stroke care (all settings) (*n* = 7), ICU (*n* = 2), or mental health services (*n* = 1) (Table 3). Studies conferred that the role of a consultee was not to provide substituted judgement, but rather to give advice aligned with the patient’s wishes and well-being. This required researchers to consult patients as much as possible about participation and encourage them to identify a consultee to advise on their behalf if they lost capacity [66]. Key to this process was discussion and shared decision-making between the consultee and the patient when they had capacity [67], and including potential consultees in early discussions with the person, while capacitous about future participation in research [47]. An observational study in ICUs involving patients (*n* = 214) considered likely to regain capacity, and family members (*n* = 445) as surrogate decision-makers, reported that the only factor significantly associated with patients’ confidence in their consultee’s consent decision was having a previous conversation about research participation [105]. Consultees also reported reduced stress from their role when they could align their advice with understanding of the person’s priorities [67]. However, a study involving nursing home residents reported no associations with patients’ characteristics and consultee decision to advise enrolment (54%, *n* = 90) (or not) [47].

While consultees were generally family members (including close friends), consent processes also allowed professional consultees or legal representatives to advise on behalf of an individual lacking capacity. However, guidance and regulation around the participation of incapacitated adults in research and involvement of consultees varied by jurisdiction. An international study on stroke (*n* = 1005, across six European countries) [87] reported that one in three patients lacked capacity to consent. The study found considerable variation in the jurisdictions’ respective enrolment requirements, detailing four different processes for adults lacking capacity as to who to approach as the proxy decision-maker (e.g. a legal guardian, next of kin, or independent physician consulting with the next of kin on the patient’s presumed will). In the USA, institutional review boards (IRBs) were reported to differ in their rates of allowing research involving incapacitated adults regardless of the risks and benefits, and in who could act as a consultee within the studies [117]. For instance, 15% of the IRBs disallowed participation in research without direct benefit regardless of risks, while 22% of IRBs accepted only an authorised proxy, spouse, or parent as surrogates, excluding adult children and other family members. Studies conducted with patients with dementia [68, 69] showed that requiring a legally appointed consultee led to declining participation from family members due to the bureaucracy and the time involved in the legal appointment. Such legal requirements could halt accumulation of evidence required for enhancing care of patients with impaired capacity.

Cluster consent was advocated for trials involving adults with impaired capacity in palliative care [48] and emergency medicine [106]. Cluster consent was undertaken by a ‘cluster guardian’ giving written agreement for treatment allocation of the defined cluster at the level of a unit, e.g. a hospital ward. This enabled the allocation of treatment at the cluster level. However, individual consent from the person, or consultee advice, was required for access to personal medical data for research purposes. Recommendations in emergency medicine research included enhancing recruitment using community consent as a similar process with ‘group consent’ for the study and individual ‘opt-out’ of the study at a later stage, for example, once capacity was regained using a similar process to deferred consent [106].

### Applying consent processes across the capacity spectrum in studies on serious illness

Thirty-two publications reported 30 original studies involving adults with serious illness and impaired mental capacity (*n* = 9046) (see Table 4). These studies were conducted across clinical settings (e.g. hospices, hospitals) and populations (e.g. elderly, dementia, delirium). Fourteen (46.6%) studies were RCTs (including feasibility), and the remaining observational including prospective and cross-sectional designs. The studies enabled analysis on the use of different consent processes and the outcome of the recruitment rate for adults across the capacity spectrum in clinical studies.

**Table 4:** Studies applying consent processes across the capacity trajectory in serious illness (*n* = 30 studies reported in 32 publications)

Authors, country, EPOC	Year	Study design and aim	Setting	Sample description	Consent process for adults with declining, impaired, or lacking capacity	No. patients/eligible (%recruited)	Key findings, challenges, and solutions
Palliative care							
Abernethy et al. [51], Australia, A3	2006	Cluster RCT 2 × 2 × 2 to test GP educational outreach visiting and case conferencing to improve patient outcomes, e.g. pain management.	Community settings	Palliative care	**Assent personal consultee, or nominated consultee**, e.g. GP capacity assessment MMSE ≤ 24 indicate require assent.	*n* = 461/607 (76%) Sample not differentiated by capacity/lack capacity	**Key findings**: strategies used enabled recruitment of largest community study in palliative care. **Solutions**: (1) study design pragmatic 2 × 2 × 2 cluster RCT and methods, broad inclusion criteria, defined recruitment plan, detailed intervention; (2) minimise patient burden, and clinician burden, e.g. research nurses collect data. **Challenges:** (1) time and resource complex trial design. (2) 7 patients ineligible no caregiver available/no pain.
Gardiner et al. [49], UK, B3	2013	Cross-sectional survey to explore palliative care need in hospital and agreement between informants.	All hospital inpatient adult ward	Palliative care	**Assent personal consultee ALC (*n*= 38). Capacity assessment MCA criteria:** HCP, family if available.	*n* = 654/1359 (48%) (mean age 78 years)	**Key findings:** 36% patients’ palliative care needs. Low identification medical (16%) and nursing (17%) staff. In 23 cases, consultees completed questionnaires on behalf of patients who lacked capacity to consent, and responses given via consultee may not be accurate. **Challenges:** non-consenting patients (*n* = 582)—(1) patient/consultee declined (e.g. too ill) (*n* = 407); (2) consultee not contactable (*n* = 109).
Rees et al. [54], UK, A3	2003	Feasibility RCT on anti-muscarinic medication (hyoscine versus glycopyrronium) for ‘death rattle’ to develop a process of advance consent enabling research to be undertaken in the terminal phase.	Palliative care ward	Palliative care	**Advance and process consent:** patients identified on admission, asked if prepared to enter study if develop secretions, informed consent documented medical notes, consent reconfirmed at readmissions.	*n* = 58/107 (54%) (*n* = 15 developed death rattle randomised)	**Key finding:** Advance consent is a viable and acceptable method to consent for trials in dying phase. Patient accrual rates to date are lower than needed to recruit adequate numbers in the time allotted to answer the research question. **Challenges**: (1) complexities of recruiting patients, e.g. too unwell (*n* = 15), died elsewhere (*n* = 16), died pre-randomisation (*n* = 15); (2) resource intense—estimate takes 3 years to meet sample size; (3) consent process time-consuming and emotionally draining. **Solutions:** (1) study across care settings, e.g. hospices; (2) involve all HCP disciplines; (3) patient acceptability trials dying phase (16 patients declined).
Whelan et al. [52], UK, A3	2013	RCT exploring the impact of the requirement for ‘proxy assent’ on recruitment in a trial of antibody response to influenza vaccination and use of a booster dose when indicated versus usual care in care homes (FEVER Trial).	All care homes in three London boroughs	Older people in care homes	**Personal consultee advice** (*n* = 82, 14%) if unavailable **professional consultee** care home staff (*n* = 40, 13%). **Capacity assessment** informal (study pre-dates MCA 2005).	Lack capacity *n* = 122/557 (22%) Capacity *n* = 155/411 (38%) Overall = 277/968 (29%)	**Key findings:** difficulties attaining consultee advise cause of recruitment bias with lower recruitment, e.g. older participants. Care home staff as consultee rarely used, e.g. reluctance of responsibility. Further research required independent risk/benefit expert panels, e.g. Independent Mental Capacity Advocates (MCA 2005). **Challenges**: (1) high lack of capacity to consent (62%, *n* = 602); (2) no contactable consultee (*n* = 304, 55%); (3) relative declined permission enrolment (*n* = 27, 5%); (4) researcher considered patient likely to resist procedures (*n* = 146, 26%).
Henwood et al. [56], Australia, B3	2014	Cross-sectional study with random sampling. Aim to establish the prevalence and risk factors to sarcopenia among older adults with compromised well-being residing in residential aged care (RAC).	Residential aged care	Very old (mean age 84.5 years) residents of the care facility	All participants were required to give **informed consent directly or by the substitute decision-maker, or by the service manager or director of nursing** following discussion with the substitute decision-maker.	102/273 (37%)—91 provided consent and 11 were consented by proxy	**Key findings/challenges:** highest reason of non-recruitment was not wanting to participate (79%), followed by ‘My GP does not want me to’ (7%), changes in health status, and death. **Solutions:** To facilitate recruitment, requires involvement of key staff members in planning and execution of the research study to support the study and understanding of the study protocol.
Myers et al. [50], USA, B3	2018	Prospective cohort study to determine whether unplanned hospital transfer can be avoided.	One large county with a single system of emergency medical services	Assisted living residents who fall	Each patient’s primary care physician informed the patient or his or her healthcare Power of Attorney of the study during usual care. **Patients** choosing to participate or their **powers of attorney** signed a **written informed consent** document.	953/1473 (65%)	**Key findings:** of the 953 residents in the study, 359 had 840 falls during 43 months. The protocol recommended non-transfer after 553 falls. Eleven of these patients had a time-sensitive condition. 549 of the 553 patients (99% [CI, 98 to 100%]) with a protocol recommendation for non-transfer received appropriate care. **Challenge:** participant accrual was slower than anticipated and time-sensitive conditions were less prevalent.
Irwin et al. [55], USA, C1	2008	Pilot study using a convenience sample to assess cognition and evaluate the presence of cognitive impairment in alert and awake hospice patients who did not have a current or past diagnosis of a cognitive disorder or cognitive impairments.	Hospice	Hospice inpatients/palliative care patients approaching the end of life (prognosis of < 6 months)	Written (*n* = 14) or oral **informed consent** (*n* = 16)	30	**Key findings:** cognitive impairments are common among inpatient hospice patients (12/30 patients were diagnosed with dementia—DSM V). Findings highlight the under-recognition of cognitive impairment in this population. **Solutions:** (1) psychoeducation for family members and caregivers, (2) psychopharmacological treatment for the patient, and (3) more timely final preparations to maximise function, adequate understanding and coping mechanisms, complete personal affairs, e.g. wills, advanced directives, legacy work and saying ‘good-byes’.
Davies et al. [53], UK, A3	2018	A feasibility cluster RCT. Aim ‘can a definitive (adequately powered) study be done?’ Hypothesis was that adequate clinically assisted hydration during the last few days of life would maintain renal perfusion and prevent hyperactive delirium (‘terminal agitation’).	Cancer centres and hospices	Cancer patients at the end of life with delirium	**Informed consent** from the patient where possible, if not **personal consultee assent** from a relative or a friend or **nominated consultee assent** from the site Study Guardian (an independent senior clinician). **Process consent**—if patient lost capacity during the study, personal/nominated consultee was required to confirm continued involvement in the study.	200/219 (91%)	**Key findings:** the study recruited 91.3% of eligible patients by using multiple consent processes. Only 13/219 (6%) declined to take part and no withdrawals. Data collection burden from the patient and family members was minimised. Informed consent was received from patients (16, 8%), advice from personal consultees (161, 81%), and nominated consultees (23, 12%). **Challenge:** Unbalanced trial arms due to differences in eligible participants and ‘competitive’ recruitment strategy. **Solutions:** Multiple consent processes and minimise data collection burden.
Dementia and geriatric care							
Baskin et al. [76], USA, A3	1998	RCT to identify challenges to informed consent in research involving subjects with advanced dementia to check purpose of bid.	Inpatient hospital	Advanced dementia	**Personal consultee advice**	*n* = 75/146 (51%)	**Key findings:** this study is the first to examine barriers to research in patients with advanced dementia. 49% (*n* = 71) eligible participants could not be enrolled in a study on palliative approaches to care. 68 because of an inability to engage the proxy in the consent process (22 because of the absence of a suitable proxy), and 4 because proxy declined consent. The findings indicate barriers in clinical research in end-stage dementia, and implications for medical decision-making in this vulnerable population. **Challenges**: (1) relative declined permission enrolment (*n* = 4); (2) proxy uncontactable (*n* = 41/63); no suitable proxy identified (*n* = 22).
Sampson et al. [40]*, UK, B3	2018	A 9-month prospective cohort study aiming to describe (1) physical and psychological symptoms, (2) health and social care service utilisation, and (3) care at end of life in people with advanced dementia.	14 nursing homes in the UK or participant’s own homes	People with advanced dementia (Functional Assessment Staging Scale 6e and above), aged 65 or over	**Personal consultee** advice, consultee uncontactable approach **professional consultee**	85/159 (54%)	**Key findings:** recruitment target not reached with 30% of patients/carers declining. Of 157 eligible care home residents, 80 people recruited by carer consultee (62, 1 patient died), or professional consultee (18). 32 declined, 28 uncontactable consultee, and 17 died. 42 people residing at home, 6 recruited by carer consultee. 16 consultees declined, 19 uncontactable, and one died.
Jones et al. [39]*, UK, B3	2012	Protocol for the above cohort study.	Care homes and own home	Dementia (early stage)	**Personal consultee advice**, consultee not contactable approach **professional consultee**	Study protocol (recruitment target *n* = 100)	**Personal consultee**, consultee not contactable approach **professional consultee**, e.g. staff not involved in study.
Black et al. [73], USA, B3	2006	Cohort study to describe health problems and comorbidities in nursing home residents, and correlations with staff identified pain.	Nursing homes	Advanced dementia	**Personal or legally appointed representative** Capacity assessment informal HCP	*n* = 126/289 (44%)	**Key findings:** physician high recognition symptoms at EoL, but detection decreases with increasing cognitive impairment indicate sample bias. **Challenges**: (1) consultee denied enrolment permission (18%); (2) no response consultee (5%); (3) patient died (23%); (4) physician denied recruitment permission (5%).
Goodman et al. [74], UK, B3	2011	Cohort mixed method design to understand EoLC needs and support for people with dementia in care homes.	Care homes (residential, 6 homes)	Advanced dementia	**Continuous consent** adults able consent in moment (*n* = 65); **personal consultee** advice adults lacking capacity (*n* = 68). **Capacity assessment MCA criteria** researcher and care staff.	*n* = 133/215 (62%)	**Key findings:** 65 (74%) people with dementia who had capacity to understand the study agreed to participate, and those lacking capacity personal consultees advised participation (68/126, 54%). **Solutions:** greatest impact on recruitment care home culture, e.g. working practices. **Challenges**: (1) consultees declined permission participant enrolment (*n* = 9); (2) consultee uncontactable (*n* = 49); (3) resource—5 months full-time researcher recruit 133. **Solutions: (**1) engagement families and care staff; (2) accessibility study information, e.g. A4 summary sheet.
Hanson et al. [75], USA, A3	2010	Cluster RCT of a decision aid to inform and support the choice between tube feeding and assisted oral feeding in dementia. To describe recruitment strategies in nursing homes and ethical approaches to minimise harm and maximise benefits.	Nursing homes (*n* = 24)	Advanced dementia	**Personal consultee**, e.g. son/daughter (68%). **Capacity assessment:** Cognitive Performance Scale and Global Deterioration Scale.	*n* = 256/425 (60%) (paired resident and personal consultee)	**Key findings:** clinical trials involving adults lacking capacity require multiple strategies to engage consultees and recruit in nursing homes. Recruitment approaches ensured adequate time to address concerns of family surrogates, and compensation for their time commitment to the study interviews. **Challenges**: (1) care home culture recruitment rate varied by nursing homes (3 to 36, 30–94%). **Solutions:** (1) study design clear potential benefits for residents; (2) engage consultees, e.g. monetary reimbursement for time; researcher frequent calling to book interview and flexibility—interviews convenient time and location/by telephone, repeated provision accessible study information; (3) engage care home staff, e.g. prior contacts, time and resource, minimal burden for care staff.
Sampson et al. [42]*, UK, B3	2015	Cohort study to define the prevalence of pain using self-rated and observational pain scales in people with dementia in the general hospital, and to examine demographic and clinical factors associated with pain and to explore a hypothesised association between pain and behavioural and psychiatric symptoms of dementia (BPSD).	Two large acute general hospitals in London	Patients with dementia who were admitted to general medical wards of two large acute hospitals	Consent process: patient to give written informed consent or with an informal carer or ‘professional consultee’. The consent procedure followed the England and Wales MCA 2005. No formal capacity assessment. A personal consultee was identified to give agreement either verbally over the telephone, and posted an agreement form to sign and return. If forms were not returned, participants’ data were destroyed. If a personal consultee could not be reached in 48 h of screening, a professional consultee advice (e.g. geriatrician not involved in the study or patient care).	230/292 (79%)	**Challenge:** 62 excluded because they did not fulfil the inclusion criteria or because carers who gave telephone agreement did not return signed forms. **Solutions:** consent process practical and feasible in the acute setting.
Scott et al. [41]*, UK, B3	2011	Protocol for the above cohort study.	Hospital inpatient	Dementia and unplanned hospital admission	**Assent personal consultee**, if no consultee identified in 48 h, **nominated ‘professional’ consultee**—senior member clinical team not involved study. **Capacity assessed MCA criteria** identify consent/assent.	Study protocol (recruitment target 250)	**Key finding:** protocol developed MCA guidance, i.e. assess the person using structured assessment for capacity, seek assent from a carer for person lost capacity, and use professional consultees when carer not contactable.
Warner et al. [72], UK, A3	2007	RCT of dementia treatment, *Ginkgo biloba*, to assess the proportion of people with capacity to consent, describe use of the MCA 2005 to assess capacity in a research setting, and identify variables associated with the presence of capacity.	Community settings	Dementia	**Informed consent** or **personal consultee advice** for adults lacking capacity**. Capacity assessed MCA criteria and MMSE.**	*n* = 42/176 (24%)	**Key findings:** cognitive testing alone is insufficient to assess the presence of capacity. Recommend researchers record outcome capacity assessment and basis for the finding; cognitive measures considered poor proxy for judging capacity. Research may be undertaken with incapacitous participants who nevertheless appear to agree to participation, but is only permissible if there is some potential direct or indirect benefit to the patient and the research cannot be carried out on patients who have capacity. **Challenges:** (1) high cognitive impairment, 76% participants assessed lacked capacity for informed consent; (2) MMSE did not predict the presence of capacity.
Ellis-Smith et al. [78], UK, B2	2018	Prospective, mixed methods feasibility study to explore the mechanisms of action, feasibility, acceptability, and implementation requirements of a measure, the Integrated Palliative care Outcome Scale (IPOS-Dem), used in routine care to support comprehensive assessment of symptoms and concerns of care home residents with dementia and their family members.	Three residential care homes registered to provide care for people aged 65 and over in a London borough, UK	Palliative/residential care home residents with dementia	The research team met with residents to ascertain willingness to participate and assess mental capacity to consent for themselves. Those residents that had capacity gave written informed consent. Advice of consultees was sought if ALC. The care home therefore sent a letter on behalf of the research team to a close friend or family member to invite them to advise on whether the resident should participate in research (personal consultee). Two letters were sent. If no response was received after 1 week of the second letter being sent, a nominated consultee was asked to advise on resident participation. The nominated consultee was independent from the research study and used all available information (including meeting with the resident, reviewing case notes, and speaking to care home staff) in order to give advice on resident participation.	10 family members approached. 22 professionals approached. 47 residents approached. 32 baseline and 30 final time point patient data. 20 professionals involved in focus groups and serial interviews. Six family members involved in a focus group and interviews	**Key findings:** of 47 participants approached, one patient provided informed consent, 45 were assessed as not having capacity to consent. Consultee approached for *n* = 39. Personal consultees provided advised for *n* = 15, and professional consultees for *n* = 24. One resident declined, four NoKs uncontactable. Not recruited on consultee advice (*n* = 3), advised against participation (*n* = 2), professional consultee (external to the care home and study) advised NoK involvement, but NoK did not respond (*n* = 1). Findings showed that possible to introduce a measure into routine care of residents and change care processes to improve resident and family outcomes. **Solutions:** mutliple procedures of approaching the NoK, and if uncontactable, a professional consultee is feasible in care homes. The staff members know the residents and could inform the researchers about preferences for research participation (or not).
Mitchell et al. [77], USA, B3	2006	Cohort study to examine EoLC in advanced dementia and to describe how research challenges were met (CASCADE study).	Nursing homes (*n* = 15)	Residents with advanced dementia and their healthcare proxies—Cognitive Performance Scale score 5/6	**Professional consultee** (healthcare staff) involving consent for themselves and providing proxy consent for residents (data collection chart review, brief clinical examination, nursing interview). **Capacity assessment:** Cognitive Performance Score 5 or 6.	*n* = 189/343 (55%) (patient/HCP dyad)	**Key findings**: involving ALC in research on palliative care, including the dying phase, acceptable to staff surrogate decision-makers in nursing homes. Findings suggest that the emotional distress of families does not decline when patients with dementia are institutionalised. **Challenges:** clinicians’ refusal (*n* = 154), e.g. too burdensome (*n* = 30), lack of interest (*n* = 53). **Solutions: (**1) nursing home culture, e.g. track record conducting research; (2) engage care home staff, e.g. researcher trained geriatric nurse, participation low burden; (3) research team credibility.
Davies et al. [84], UK, B3	2010	Cohort study to examine health trajectories and outcomes of people aged 85+ cohort and associations with biological, medical, and social factors.	Usual place of residence, e.g. at home, care home	Older people aged > 85 years	**Advance consent**—consent document participant nominate a **personal consultee** should they lose capacity. Used continuous consent process, fluctuating capacity awaited re-gain capacity. **Capacity assessment. MCA criteria researcher**.	*n* = 1042/1453 (72%)	**Key findings:** 72% recruitment rate using consultees, separate consent protocols for participants in care homes and flexibility regarding those with fluctuating capacity. Cognitive impairment is common requiring consideration of the relevant ethical and legal issues. **Challenges:** (1) consultee declined recruitment (*n* = 5); (2) consultee uncontactable (*n* = 9). **Solutions:** (1) comprehensive protocols for consent and capacity assessment that anticipated high cognitive impairment guided by legislation; (2) training researchers on consent and capacity processes, sensitively handling complex situations; (3) time and resource to engage family and care home staff; (4) follow-up telephone call/visit 1 week after receipt study information; (5) design study information for older person, e.g. font size, language.
Mason et al. [83], UK, A3	2006	RCT to determine effectiveness and cost-effectiveness of pressure relieving mattresses on pressure ulcers.	10 research centres involving inpatient units	Patients with pressure ulcers aged > 55 years with or without cognitive impairment	**Personal consultee advice for ALC**.	Lack capacity *n* = 87/2445 (4%) Capacity *n* = 1972/2395 (82%)	**Key findings:** a higher proportion of relatives declined enrolment, than patients themselves. Proxy consent allowed only a small increase in trial recruitment. **Recruitment challenges**: (1) no relative available (*n* = 2286); (2) relatives declined permission to enrol (*n* = 72/159, 45%).
Botker et al. [85], Denmark, A3	2018	RCT. Aim to examine if the addition of brain natriuretic peptide measurement to the routine diagnostic work-up by prehospital critical care team physicians improved triage in patients with severe dyspnoea.	Prehospital critical care units at Central Denmark Region hospitals	Patients with severe dyspnoea	**Deferred consent:** included patients prior to informed consent. Subsequent oral and written informed consent was obtained from all participants or next of kin by one of 10 study investigators.	711/747 (95%)	**Key findings:** 28 (4%) participants withdrawn consent based on patient or proxy request. 7 (0.9%) patients were excluded as consent could not be obtained. The routine addition of prehospital NT-proBNP measurement did not improve the triage of patients with dyspnoea of cardiac cause directly to the department of cardiology and did not significantly improve treatment or patient outcomes.
Galeotti et al. [79], Italy, A3	2012	RCT. The ADCare study aimed to evaluate the long-term safety and efficacy profiles of three atypical antipsychotic drugs and one conventional antipsychotic drug in treating psychosis, aggression, and agitation in outpatients with AD. The aim of this paper is to report the ADCare study experience and to analyse in depth the possible reasons for the low accrual.	N/A	Alzheimer’s disease patients	**Informed consent**	83/~ 800 eligible patients from 19 clinical centres	**Key findings:** ~ 800 eligible patients in 1 year were identified in the clinical centres. Although initially willing to participate, most of these patients/family members declined because a legal court nomination was required to participate in the trial. Court nomination was perceived by patients and family members as intrusive and potentially creating conflicts in families. **Challenges:** only 9 clinical centres participating in the study enrolled patients with the involvement of a legal proxy, even with initiatives to disseminate information on legal agency for research participants. The main obstacles were caregivers’ reluctance to designate a legal proxy and court delays.
Intensive care							
Day et al. [109], UK, B3	2015	Pilot RCT single centre. Aim to report challenges to undertake the RCT and feasibility of delivering and evaluating a complex intervention in a critical care unit.	Two mixed critical care units of a large inner London hospital	Mixed medical, surgical, and trauma patient population requiring either level 3 (intensive) or level 2 (high dependency) care	Researchers not involved in data collection were responsible for the consent process. In this study, patients were willing to **consent verbally but deferred to their relative to provide written consent**. If ALC, their relative provided advice, the researchers then ensured that when patients regained capacity and they requested an informed consent.	158/221 (72%)	**Key findings:** rushing the decision-making process was a concern. Some patients approached felt unable to say no, but kept saying come back later. This may indicate concern that their care may be affected if decline. Viewing informed consent as a process and revisiting it throughout the trial period to ensure participants can recall providing consent. **Challenges:** Most of those approached felt too tired or lacked the concentration to read the study information. Patients with no appropriate consultee were excluded. **Solutions**: (1) avoid consent bias, risk of coercion and allow more time for decision-making. (2) Participant information sheet clear and concise. (3) To review capacity in patients with memory loss.
Bench et al. [110], UK, A3	2015	Pilot RCT. Aim (1) providing an initial evaluation of a user-centred critical care discharge information pack (UCCDIP), (2) inform decisions about its further development and evaluation, and (3) estimate the sample size for a full trial.	Two ICUs within a single teaching hospital in central London	Mixed medical, surgical, and trauma patient population requiring either level 3 (intensive) or level 2 (high dependency) care	**Informed consent** from the patient was then obtained prior to data collection on the ward. For ALC, **informed written consent at the point of ICU discharge (deferred), personal consultee declarations**, usually from the patient’s next of kin, were sought. The relatives of all recruited patients were given study information when they visited the ICU or telephoned and invited to participate. Written consent was obtained from relatives during their next hospital visit.	158/221 (36 declined to participate) (72%)	**Key findings/challenges:** The a priori enrolment goal was not reached, and attrition was high leading to insufficient statistical power to determine outcome benefit. 101 (64%) patient participants provided primary outcome data at time point 1. A total of 48 (60%) patients’ relatives provided at least one set of outcome data. Twenty-seven (17%) patients and 32 (40%) relatives were lost to any follow-up.
Higginson et al. [111], UK, B3	2016	Prospective ethnographic study. Aim to explore the nature and patterns of decision-making processes during ICU admission, including sources of conflict and resolution.	Two ICUs in an inner city hospital serving an ethnically and socially diverse population	ICU patients where clinicians had potential end of life concerns, discussions or a high risk of dying during the current admission	Where possible, **informed consent** from **patients**. For patients who lacked capacity, following the Mental Capacity Act 2005, firstly capacity was assumed to be present, unless proven to be absent. Clinicians assessed capacity. If lacked capacity, approach for advice a **personal consultee** (e.g. family member), if not identified **a nominated consultee** (e.g. clinician)	16 patients and 19 relatives	**Solutions:** the researchers looked for instances where capacity was present and discussed with the clinicians. The research team ensured that the nominated consultee was not part of the research team. Relatives also provided informed consent/assent for interviews and observations.
Rouzé et al. [112], France, A3	2017	RCT to test the hypothesis that the use of an algorithm based on fungal biomarkers would increase the percentage of early discontinuation of empirical antifungal treatment among critically ill patients.	Mixed 50-bed ICU department of the University Hospital of Lille, France	ICU patients with *Candida* infection	An **informed written** consent was obtained before randomisation from the **patients or their proxies.**	110/387 (28%)	**Key findings:** 8/510 patients who were assessed for eligibility refused. One patient withdrew consent after receiving the biomarker strategy. The use of a biomarker-based strategy increased the percentage of early discontinuation of empirical antifungal treatment among critically ill patients with suspected invasive *Candida* infection.
Mental health							
Ho et al. [97], Australia, B3	2018	Methodological paper aiming to describe an informed consent process used when recruiting persons with intellectual disability for a study which is currently investigating falls among people with intellectual disability, and to reflect on the methods of informed consent used.	Community—participant’s home, small group homes with up to two to four co-inhabitants with paid support	People with intellectual disability	**Informed consent and proxy advice** from the participants themselves where possible. The process was designed to provide a collective perspective of the capacity of the person with intellectual disability to consent. The researcher uses repeated observations to establish if the person has capacity to provide consent, and consult with the caregiver. Use of a decisional questionnaire involving score of > 3 out of six suggest capacity to provide informed consent. **The next of kin, family member, or caregiver is asked to be present during this process to provide a supportive, comfortable environment and to provide oversight to the discussion.**	[84] 40/68 (59%)	**Key findings:** only 3 out of 40 participants were able to provide informed consent. 22 participants were able to have a discussion about their involvement in the study with the support of their caregiver. 15 participants were unable to engage in the process. The decisional questionnaire gave an accurate representation of participants’ decision-making capacity. **Challenges:** more than 40% caregivers declined taking part in the study on behalf of the person. This consent process was time-consuming. **Solutions:** Training and building relationships with patients and their caregivers.
Ramerman et al. [96], Holland, B3	2018	Experimental study to examine challenging behaviour, physical symptoms, and quality of life associated with antipsychotic drug.	Hospital intellectual disability mental healthcare clinics	Adults with intellectual disabilities	Written informed consent was obtained from participants and/or their **legal representatives.**	159 (no data on number of people approached)	**Key findings:** mean age was 46.2 (SD 17.7). Health quality of life (HQoL) was negatively associated with both symptoms of challenging behaviour and physical symptoms associated with antipsychotic drugs. No data on differentiation proxies and patients in informed consent process.
Delirium							
Marcantonio et al. [101], USA, A3	2010	RCT to determine whether a delirium abatement programme (DAP) can shorten duration of delirium in new admissions to postacute care (PAC).	Hospital	Patients with delirium older than 65 (mean age = 84); MMSE and CAM used to assess capacity	In those who had delirium, family caregivers, acting as **proxies**, provided **informed consent** for trial participation.	457/667 (69%)	**Key findings:** this study was the largest cohort of patients with delirium ever enrolled in a research study. Facilities received a small incentive based on their performance. Clustering effect widened the confidence intervals. Possible to ascertain adherence and outcomes only in trial participants whose proxies provided informed consent to allow medical records to be reviewed. **Challenges:** Of 667 patients who were eligible, 138 relatives refused (21%), 56 relatives uncontactable (8%).
Cole et al. [102], Canada, A3	2002	RCT to determine if systematic detection and multidisciplinary care of delirium in older patients could reduce time to improvement in cognitive status.	Hospital	Older patients 65 years old or more admitted to a general hospital medical service	**Informed consent** was obtained from the **patient or substitute decision-maker.** Confusion assessment method was used to assess capacity.	227 /299 (76%)	**Key findings:** 72 were excluded as they could not provide consent. Study was not powered to detect any statistically significant differences.
Emergency medicine							
Offerman et al. [108], USA, B3	2013	Observational prospective multicentre study to describe the rate of successful consent using an altered (deferred telephone) consent process in emergency department (ED) patients.	Emergency department	Adults who had attended ED with blunt head trauma	**Informed consent** was obtained from the **patient or substitute decision-maker.**	506	**Key findings:** follow-up telephone contact was successfully accomplished in 501 of the 506 subjects (99.0%; 95% CI = 97.7 to 99.7%). Consent for study inclusion and conduct of the telephone survey was obtained in 500 of 501 subjects at time of the follow-up call (99.8%; 95% CI = 98.9 to 100.0%). Surrogate consent was obtained in 199 of the 501 subjects (39.7%; 95% CI = 35.4 to 44.2%)

Most studies were conducted in palliative care (*n* = 8) or dementia/geriatric care (*n* = 10). The studies used a breadth of consent processes tailored to the respective population. Methods included personal and/or professional consultee advice, advance and process consent, enhanced informed consent, and deferred consent. Processes showed variation in recruitment of eligible participants (range 23.9% [72] to 78.8% [42]). Use of a personal consultee alone (e.g. a family member) was uncommon and showed the greatest variability in the recruitment rate. Four studies used personal consultee advice only for adults lacking capacity [49, 50, 83, 108]. The recruitment rates varied by study population and study design. In a cross-sectional survey exploring patients’ palliative care needs in hospital, the overall recruitment rate was 48.1% (654/1359) [49]. While most eligible patients lacking capacity had an identifiable personal consultee able to advise, 8.1% of eligible patients (*n* = 109 /1359) were not approached as no personal consultee was available. In contrast, in an RCT [83] on reducing pressure ulcers, only 3.6% (*n* = 87/2445) of eligible patients who lacked capacity were recruited compared to 82.3% (*n* = 1972/2395) of patients able to giveinformed consent. The main reason for non-recruitment was no available personal consultee (93.5%, 2286/2445), and when available, 45.3% declined to give advice (*n* = 72/159).

Three studies used both a personal and a professional consultee to provide advice with an initial approach to a personal consultee, and if unavailable an approach to a professional consultee [51–53]. Abernethy and colleagues [51] achieved a high recruitment rate (*n* = 461, 75.9%) using this joint approach in a low-risk non-invasive cluster RCT on palliative care in community settings. A MMSE score of ≤ 24 indicated the need for consultee advice. Only seven patients were ineligible due to consultee unavailability. The recruitment process used a resource-intensive defined recruitment plan that sought to facilitate recruitment by minimising patient and clinician burden. Similarly, a feasibility cluster RCT of clinically assisted hydration in cancer patients in the last days of life used multiple processes of informed consent for patients with capacity (*n* = 16, 8%), and advice from a personal (*n* = 161, 80.5%) or professional consultee (*n* = 23, 11.5%) for adults lacking capacity, and process consent throughout the study [53]. The multiple consent processes enabled a 91.3% recruitment rate (*n* = 200/219) of eligible patients from four cancer centres and eight hospices. Only 5.9% (*n* = 13/219) of the eligible participants declined to participate, and none withdrew. Using clinical observations for data collection minimised burden for patients and family members. However, a competitive recruitment strategy caused variation in recruitment rate between clusters and imbalance in the trial arms (*n* = 73 treatment arm and *n* = 127 control arm). Conversely, an RCT of antibody response to influenza vaccination for older people in care homes incorporated multiple processes of consent [52]. But care home staff were hesitant to act as a professional consultee in the absence of a personal consultee. A total of 304 (54.5%) eligible individuals were excluded for this reason, and recruitment of only 8.9% of eligible residents who required a professional consultee (*n* = 40/448). However, the study acceptable was mixed with 75.2% of personal consultee advising enrolment (*n* = 82/109), but only 37.7% of residents with capacity consented to recruitment (*n* = 155/411). Greater clarity on the responsibility of the professional consultee was required to enable care staff to act in this role, particularly in a trial involving invasive procedures.

Seeking advice from a consultee on enrolment was prominent in studies involving patients with dementia or delirium. Depending on the context of the study, recruitment rates ranged from 23.9% [72] to 78.8% [42]. Most studies in dementia took place in nursing homes/residential care homes [40, 42, 72–75], with only two RCTs conducted in hospital both on delirium [101, 102]. Common challenges to participation were relatives advising against patient enrolment [40, 73, 101], unavailability of a personal consultee [74, 76, 101], and care home staff acting as professional consultees declining to give advice for 44.9% of the eligible participants [77]. To overcome potential hesitancy from consultees required active engagement with consultees, keeping in touch and being flexible, highlighting potential benefits, and lowering burden for the consultee [75]. To minimise exclusion of eligible patients due to unavailability of personal consultees, studies used processes of a professional consultees and a clear recruitment strategy detailing the procedures [42, 78]. A cohort study involving people with dementia from six care homes achieved 62.1% patient recruitment rate (*n* = 133/215) using a process of informed consent when possible (*n* = 65/89, 73.8%) and personal consultees (*n* = 68/126, 54%) [74]. The study reported the importance of engaging with patients and families by using a short summary sheet to enable understanding and participation. However, 49 eligible patients had no available personal consultee and were excluded [74].

Two studies [54, 84] in palliative care employed advance consent, followed by process consent (or personal consultee) processes. In a feasibility RCT of two medications for the treatment of death rattle, 54.2% (58/107) of eligible patients with capacity provided advance consent. However, due to the complexities of the patient population and uncertain prognosis, only 25.9% (15/58) of patients were randomised [54]. The consent process was resource intensive to recruit the target sample size and emotionally draining for patients as it required conversations about an event which may not occur. Most patients accepted the advance consent process, with only 16 of 58 declining. A prospective cohort study examining health trajectories and outcomes in patients over 85 years old in usual place of residence achieved a 72.0% (1042/1453) recruitment rate [84]. Individuals who provided advance consent were also asked to nominate a personal consultee. Throughout the study, capacity of the recruited participants was assessed, and the nominated personal consultee was contacted if needed. Only five consultees declined, and nine were unavailable. The recruitment process was facilitated in multiple ways including using different consent and capacity assessment protocols for respective settings, tailoring the study information to the target population, flexibility towards those with fluctuating capacity, identifying personal consultees, training researchers, sensitively handling complex situations, and allocating time and resource to engage and keep in touch with family and care home staff.

Four studies that took place in ICUs used informed consent and consultee advice, and deferred consent processes [109–112]. Pilot RCTs of complex interventions (non-invasive) used deferred consent processes [109, 110]. Patients agreed verbally to study enrolment, and their personal consultees gave written advice. Once patients recovered, they were approached for an informed consent [109]. The study achieved 71.5% recruitment rate. However, the possibility of coercion was raised with some patients indicating they felt unable to decline verbal enrolment, and consent bias with the exclusion of patients with no personal consultee. In another pilot RCT, the next of kin of 83.6% of patients who lacked capacity in the moment of data collection provided deferred advice at the point of discharge. Although this study had a 71.5% recruitment rate, 16.3% declined to participate. A further RCT involving both adults with capacity to provide informed consent and personal consultee advice for incapacitated patients showed only 8/510 eligible individuals declined participation [112]. Similarly, an RCT involving a blood test for patients with severe dyspnoea used deferred consent [85] and showed high acceptability with only 3.8% (28/747) withdrawing consent once regained capacity or on consultee advice. Only 0.9% (7/747) were excluded as unable to obtain deferred consent or consultee advice. An ethnographic observational study in ICU used informed consent and personal or professional consultee advice successfully to recruit 16 patients and 19 relatives with a clear consent protocol aligned to minimal risk of participation [111].

Enhanced consent processes were limited to studies involving adults with intellectual disabilities [96, 97] and in palliative care [55]. A study investigating falls in people with intellectual disability aimed to maximise an individual’s ability to provide informed consent for themselves achieved a recruitment rate of 58.8% (*n* = 40/68). The study enhanced consent processes by involving family members and healthcare professionals in the process, using a questionnaire to assess capacity, and conducting multiple observations [97]. A personal consultee was sought for adults lacking capacity. However, over 40% of relatives advised non-participation in the study on behalf of the person as they considered the consent process too time-consuming and burdensome. A cross-sectional study on use of antipsychotic medication for people with intellectual disabilities recruited 159 eligible participants using informed consent and professional consultee comprising legal representative [96]. However, no denominator was stated. Finally, a pilot study of patients admitted to a hospice exploring levels of cognitive impairment sought to minimise the burden of informed by allowing written or oral informed consent [55].

### Public attitudes on enrolling adults lacking capacity in research

Studies (*n* = 22) on attitudes towards enrolling adults lacking capacity in research consistently reported the acceptability of involving consultees to enable recruitment. However, the level of acceptability varied associated with the nature and purpose of the study (see Additional file 3: Table S8 reporting the studies’ key findings, and suppl. 9 – additional reporting [130–133]). Most studies (59.1%) were conducted with public members (including researchers, healthy populations, IRBs) and used observational designs (e.g. survey) to explore attitudes about research with critically ill patients (e.g. conducted in intensive or emergency care settings), or focused on Alzheimer’s disease, stroke, cancer, intellectual disabilities, overall incapacitated individuals, or proxy decision-making for study enrolment. Overall, stakeholders considered it acceptable to use substitute decision-making to enrol adults lacking capacity in research, especially for low-risk studies. Acceptability focused on prioritising a person’s wishes and the potential benefits for the person, rather than the burden of acting as a proxy decision-maker.

### Transparent expert consultation

The systematic review findings identified three critical areas debated in the TEC including:

1. Time and design of the consent process: ‘How can we enhance the timeliness of the consent processes for adults with fluctuating or deteriorating capacity in research on palliative and EoLC?’
2. Enhancing consultee and supportive decision-making: ‘How can we enhance proxy decision-makers’ role in the process of consent for adults who lack capacity?’
3. Ethics, resources, and expertise: ‘What are the key considerations in planning the consent process in studies involving adults lacking capacity and communicating this to a research ethics committee?’

The TEC stakeholder workshop involved 39 participants of the 83 invitees (47.0%). The participants represented service users/lay voluntary sector representatives (*n* = 13, 33.3%), researchers (*n* = 15, 38.5% including ethicists), clinical academics (*n* = 9, 23.1%), and clinicians (*n* = 2 5.1%). Voluntary sector representatives included lay members who were carers and/or patients living with a progressive condition recruited from charities for people with dementia (Age UK and Alzheimer’s Society) and cancer/palliative care (Brainstrust, Marie Curie, Independent Cancer Patients’ Voice, National Cancer Research Institute, and the then National Council for Palliative Care). The group generated 184 recommendations on the three areas (area 1, *n* = 60 recommendations; area 2, *n* = 72 recommendations; and area 3, *n* = 53 recommendations). Following data analysis, 29 recommendations were presented in the online Delphi Survey round 1 (see Additional file 4: Table S10 - Delphi participant characteristics). The recommendations pertained to timeliness and design of the consent process (area 1, *n* = 7 recommendations), enhancing consultee and supportive decision-making (area 2, *n* = 10 recommendations), and ethics resources and expertise (area 3, *n* = 12 recommendations) (see Additional file 4: Table S11 – Delphi round 1 recommendations and Fig. S12 – round 1 box and whisker plots). The Delphi survey round 1 involved the workshop participants (*n* = 39) and individuals unable to attend the workshop (*n* = 4). 51.8% participated (*n* = 43). Round 2 involved the round 1 respondents only (83.7% response rate, *n* = 36). Findings from round 1 analysing 454 free-text comments informed revisions to 11 recommendations to reduce ambiguity. This mainly concerned the use of the term ‘consultee’ in relation to legislation. In round 2, consensus was apparent for 24 recommendations with strict/broad agreement and five considered equivocal (see Additional file 4: Table S13 - Delphi round 2 recommendations and Fig. S14 – round 2 box and whisker plots). No recommendations were not indicated. The top recommendations by respective areas were as follows:

- *Area 1*. ‘Information about a research study is comprehensible, short and written in accessible language’ (R2, median 9, IQR 9–9) and ‘Although the legal significance of advance consent will vary depending on the relevant legal framework, it is good practice for researchers to seek an advance consent while individuals have capacity to consent for themselves, for example, shortly after a diagnosis of a progressive illness’ (R10, median 8, IQR 7–9)
- *Area 2*. ‘The consent process is tailored to the individual’s needs, capabilities and values with researchers observing for non-verbal and verbal cues that may indicate an individual may wish to withdraw’ (R9, median 9, IQR9–9) and ‘Advance care planning to include discussing and recording in a Statement of Wishes document an individual’s nominated or personal consultee whose opinion on participating in a research study is sought if the patient loses capacity’ (R21, median 8, IQR 7–8)
- *Area 3*. ‘Researchers to demonstrate to Research Ethics Committees a clear process of consent for potential participants with compromised capacity that details how the researchers will proceed to tailor the consent process to maximise individuals’ ability to consent for themselves and when and how they will seek an opinion from a consultee’ (R29, median 9, IQR9–9) and ‘Health and social care practitioners to recognise research as a core clinical activity in a similar way as teaching and training’ (R23, median 9, IQR 9–9)

Three main areas were considered equivocal and were debated in the expert ‘think-tank’, and solutions proposed by consensus (see Additional file 4: Box S15 – main areas and top solutions). Nineteen experts attended the think-tank, representing researchers/clinical academics (*n* = 12), clinicians (*n* = 2), lay voluntary sector representatives (*n* = 3), and PPI member (*n* = 1). The three equivocal areas comprised the following:

- *Area 1*. Involving and supporting consultees in the decision-making process with uncertainty on how best to support consultees to engage in the research process, with four recommendations indicating equivocal broad agreements (recommendations 6, 8, 15, 22, 27)
- *Area 2*. Practitioner training and education with agreement on the requirement to increase training and support to researchers and clinicians, but areas of uncertainty concerned for example how to provide and disseminate training and guidance, and how to fund (R5)
- *Area 3*. Legislative frameworks and the incorporation into research practice

A prominent equivocal area surrounded the Mental Capacity Act 2005 [29] legislation in England and Wales. The key solutions proposed concerned amendments in the supporting guidance for the Act to enable greater flexibility in the role of the ‘professional’ consultee in, for example, how they are identified. The Expert Panel concurred that the MCA process of review of an advance consent being upheld (or not) by a consultee if a participant lost capacity to consent should be applied to all clinical trials and not limited to trials of non-pharmacological interventions. This would include for example pharmaceutical trials where advance consent is upheld without requirement for consultee review if a person loses capacity in European legislation [134].

## Discussion

**Fig. 3:**
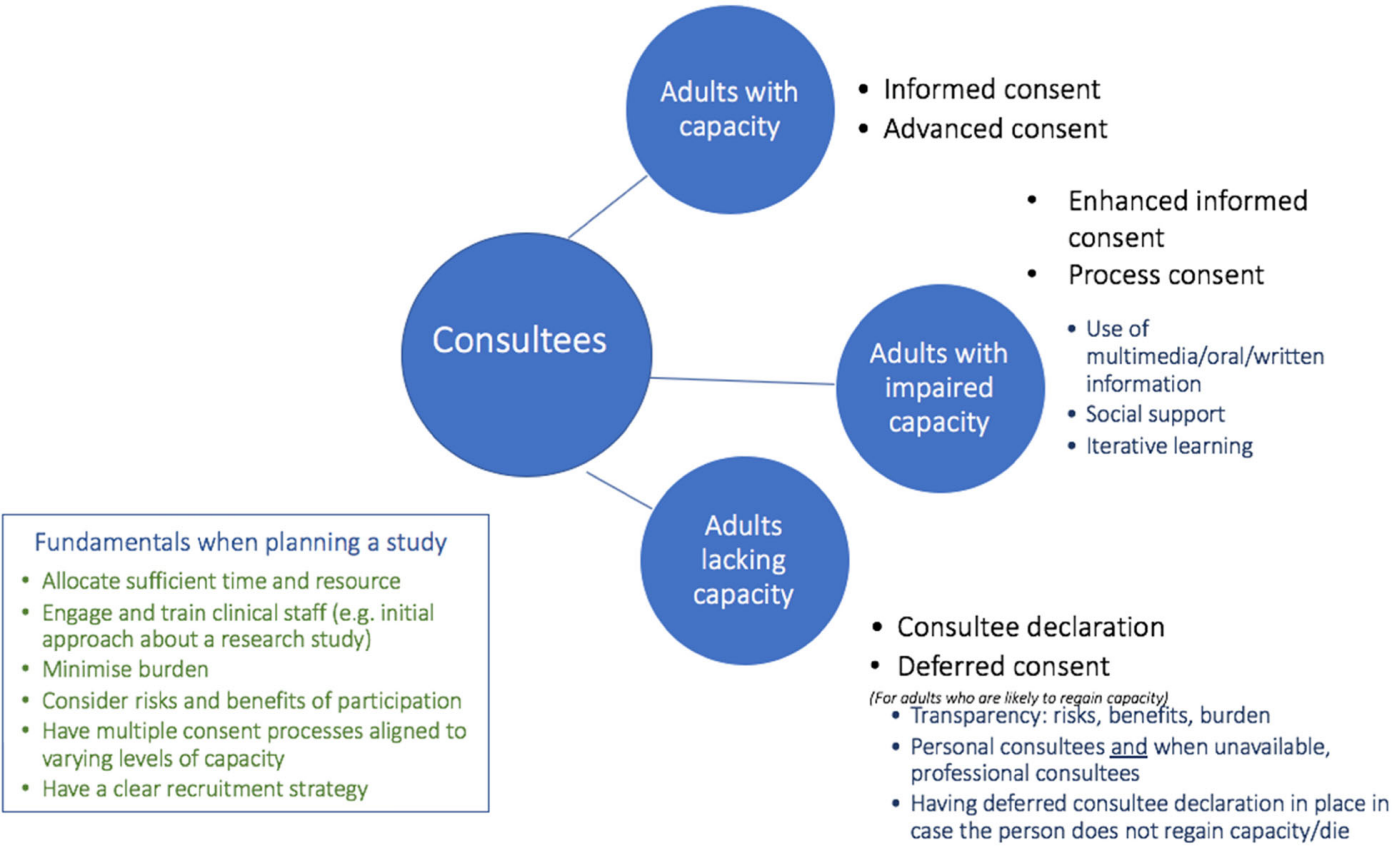
How ‘best’ to include adults nearing the end of life in research

This synthesis of evidence from systematic review and TEC identified challenges and solutions to including individuals across the capacity spectrum in research on EoLC. Our findings produce the MORECare_Capacity statement detailing 20 best practice solutions and implementation requirements to maximise study participation across the capacity spectrum (Table 5 and Fig. 3). The statement provides much needed guidance to maximise opportunities for adults across the capacity spectrum to participate in research. It is relevant for researchers, members of research ethical committees, individuals overseeing research governance, clinicians, the public, service users, and voluntary sector representatives. The statement details solutions for study designs at the participant level to maximise individual autonomy, enhance the contribution of proxy decision-makers, and ensure time and resources to enable participation and recruitment strategies that anticipate and plan for varying and changeable levels of capacity. At the structural level, key activities are engaging research ethical committees in how to include adults lacking capacity in research by auditing ethical approvals to review decision-making and inform research practice, and promoting the importance of research in health and social care to improve clinical care and raise awareness on recruiting adults from across the capacity spectrum to enhance the applicability of research findings for service users. The statement is intended as a framework for ‘best’ research practice. A statement cannot always anticipate change in an individual’s circumstances or wishes. Ongoing review is always required by the consultee and researchers of an individual’s best interests.

**Table 5:** MORECare_Capacity statement solutions on recruiting adults with impaired mental capacity at the end of life in research

	Solutions
Ethics	1. Researchers should design all aspects of the study in the context of potential *risk*, *burden*, *and benefit* of study participation.
	2. Institutional review boards and research ethical committees should have transparent decision-making processes to ensure consistency on ethical approvals for studies from various health specialties involving adults across the mental capacity trajectory.
	3. The individuals (e.g. relative, friend, formal carer, nursing home staff, healthcare staff) who can act as a consultee to advise on whether the individual would have wanted to participate in the research study had they had capacity should depend on the nature of the study, rather than legal restrictions.
	4. Researchers should be able to demonstrate a clear process of consent for potential participants with compromised capacity that details how the researchers will tailor the consent process to maximise an individual’s ability to consent for themselves and when and how they will seek an opinion from a consultee.
Maximising individual autonomy	5. Clinicians should engage in research participation conversations with patients at the early stages of illness, discussing varying levels of risk, burden, and benefit, and document the person’s preferences and wishes in for example an advance directive.
	6. Individuals who are likely to lose capacity should be asked to designate a consultee whose opinion on their participation in a research study will be sought if the individual loses capacity.
	7. For individuals who have capacity to consent in the moment, but overtime may not remember the discussion, process consent should be adopted whereby researchers re-confirm the individual’s wish to participate at each data collection time point.
	8. Researchers should check for non-verbal (e.g. agitation) and verbal cues (e.g. ‘I’m unsure why you are asking me’) that may indicate a wish to withdraw during the study.
Involving consultees	9. Personal consultees should be present in research participation conversations with patients at the early stages of illness.
	10. Where possible, to improve social support, personal consultees/family members should be engaged in the (enhanced) informed consent process for adults with impaired capacity.
	11. The nature and extent of the responsibility of a consultee acting on behalf of a patient should be clarified.
	12. Establish a national body to provide support and information to family members/informal carers acting as personal consultees.
Tailoring recruitment process to need	13. Researchers should incorporate in the study design research participation information and (where applicable) data collection tools in multiple formats (e.g. verbal, written, electronic).
	14. Studies where potential participants are expected to be adults across the mental capacity trajectory should incorporate multiple consent processes (e.g. personal and professional consultees, informed and process consent).
Time	15. When possible, potential participants should be allowed time for further discussions regarding their research participation decision.
	16. The study design should allow time proportionate to the risk, burden, and benefit of participation for consent or consultee decision (before or after research participation if consent is deferred).
	17. Researchers should build in the time required to engage with and train clinical staff who will be involved, and those who might act as professional consultees in the study.
Enhancing the research culture and infrastructure	18. Health and social care practitioners should recognise research as a core clinical activity in a similar way as teaching and training.
	19. Clinicians should be supported and provided with training to ensure they are confident in their skills to discuss research studies with patients (and/or family members) during routine clinical contact.
	20. Health and social care practitioners should support adults across the mental capacity trajectory at all stages of the research study by considering the person’s best interests and individual wishes and preferences to uphold individual autonomy and minimise the risk of harm, and enable family members to act as a personal consultee by ensuring sufficient information and understanding about the role.

Our findings demonstrate that despite ethical challenges and legislative requirements, recruiting adults with impaired capacity in research is possible with careful consideration of the context, study design, and the resources required to enable participation. The main solutions for maximising research with incapacitated adults centred around early involvement of the person while they had capacity to indicate their preferences for research participation, and nomination and engagement with consultee(s) in these discussions. It is important to recognise that acting as either a personal (e.g. family member) or nominated (e.g. clinician) consultee can be burdensome, with burden increasing in studies which carry risks or little benefit the patient directly. A priority for consultees is to have sufficient time and information about their role and to have an awareness of the person’s wishes through earlier discussions and documenting a person’s wishes about research participation in, for example, an advance directive [21] or study specific in an advance consent. Figure 3 summarises the different processes across the capacity spectrum and sets out key factors to anticipate and plan for in a research study. Incorporation of our solutions in research studies on EoLC could increase the potential to recruit a representative sample of those intending to benefit from palliative care services and treatments. Involving patient groups with impaired capacity would increase generalisability of research findings by not limiting the evidence base to those with capacity and less advanced disease, and sustainability of the tested interventions in clinical care. Our solutions are informed by methodological and original research studies, and expert consensus. Importantly, surveys of the public and stakeholders consistently endorse the involvement of adults with impaired capacity in research and specifically in palliative care [135].

Ethically, research studies must endeavour to ask questions important for care and treatment, be designed to enable participation and minimise potential for harm, and demonstrate to research ethical committees a clear rationale for the study design and processes of consent for adults across the capacity spectrum (R29). In turn, ethical research committees must ensure transparency and consistency on ethical approvals for adults lacking capacity (R28). Working in this way seeks to uphold individual rights of, for example, autonomy to participate (or not), beneficence, and justice [136]. Beneficence in research on EoLC is important. Individuals with advanced disease are unlikely to benefit from the research findings seeking to improve care and treatment, but participation can enable a sense of contribution to society, of their ‘voice’ being valued and foster a sense of altruism [137]. Excluding individuals because of perceptions of vulnerability associated with advanced disease and impaired capacity may be considered unjust [3]. It denies equal opportunity for individuals living with progressive conditions to contribute to care and treatment in palliative care across the illness spectrum. To facilitate participation in research, our findings identify two key areas: ‘enabling and empowering consultees’ and ‘a carefully constructed and resourced recruitment strategy’.

### Enabling and empowering consultees

While techniques for enhancing autonomy are imperative to maximise individual decision-making, for adults with impaired capacity in EoLC studies, proxy decision-makers were crucial to enable recruitment. Consultees as proxy decision-makers sought to represent patients’ wishes and best interests [105]. Their role was vital in enabling participation in research for adults across the capacity spectrum. While an array of consent processes can be implemented depending on the condition and level of capacity of the individual, successful participation of individuals relied on empowering and educating all those involved (patients, families, health and care staff, researchers, ethical committee members). Introducing patients to research studies while they were competent, and their family members, empowered patients and their families. Careful consideration is required to identity the right proxy decision-maker. Consultees sought to consider the benefits, risks, and burden to the person, and align their advice with understanding of the person’s wishes. Earlier conversations enabled family members to align advice on participation with understanding on the person’s wishes, particularly in comparatively high-risk studies [47, 67]. Similarly, West et al. in their systematic review on ethical challenges in dementia research reported differences in consultees advising enrolment with a higher proportion willing to provide consent for non-invasive studies compared to invasive studies, even with no potential direct benefit for the patient [21]. Our findings show that the presence of family members during enhanced consent processes enabled study participation by increasing opportunity and support for the person to discuss their priorities [44, 86]. Although personal consultees were crucial, studies reported higher recruitment rates when they used a nominated/ professional consultee to advise if no personal consultee was available (compared to designs with a personal consultee alone). In contexts where being a professional consultee was unfamiliar, support and training for professional consultees were required to increase confidence and understanding of their role, for example, in a care home with little/no embedded research culture.

### A carefully constructed and resourced recruitment strategy

Recruitment strategies in research on EoLC must align with the target population and anticipate and plan for varied and changeable levels of capacity associated with progressive disease. Even with strict eligibility criteria, potential participants within one study will likely present with varying and changeable levels of capacity. Our findings identify the necessity to have multiple consent processes in place aligned to the target population, context, and study aim to recruit individuals across the capacity spectrum [51, 53]. Studies on EoLC demonstrate the feasibility of using multiple processes in, for example, trials in the dying phase [54] and observational cohort studies [40, 77]. The studies demonstrate the resource intensity and careful planning required to implement complex processes of advance consent when the person had capacity, and if/when capacity is lost, the involvement of proxy decision-makers (e.g. family or professional). Boland et al. echo these findings in their systematic review on recruitment strategies in trials involving people with serious illness [26]. Our findings show that where possible, researchers sought to maximise individual’s autonomy, through consent processes such as enhanced, advanced, deferred, and process consent, and when lacked capacity incorporating a personal and/or professional consultee declaration that sought to align with understanding of the person’s wishes and preferences. Enhanced consent was feasible and acceptable to augment understanding and reasoning by using methods of multiple formats and involving family members [61]. Similarly, Hostiuc et al. reported from their meta-regression that using multimedia to present study information significantly improved understanding, reasoning, and appreciation and enabled informed consent for adults with schizophrenia in clinical trials [22]. There is clearly opportunity to incorporate advances from other specialities, but with consideration if a successful strategy in one population is transferable to another. Different conditions impair different components of cognition, with variance in transience and progression (e.g. between schizophrenia and dementia), and prospect of recovery or not (e.g. between emergency medicine and palliative care). Further process evaluation of multimedia techniques is needed to determine the content and duration as the active ingredients of the techniques which leads to enhanced decisional capacity in studies for specific populations. Toolkits are useful resources to inform consent processes that encompass the heterogeneity of for example palliative care populations and the legislative framework for the respective jurisdiction and context [138] (*See page 96 in for example* [139]).

### Limitations

Our incorporation of systematic review and expert consultation and consensus enabled consideration of a breadth of evidence and the application in study designs to enable participation across the capacity spectrum. The search strategy enabled consideration of a breadth of research methods employed in different clinical populations and contexts required to underpin the MORECare_Capacity statement. But the breadth of our strategy may have limited the identification of all relevant original research studies involving adults with serious illness and impaired capacity. While we present evidence from an array of original research studies including qualitative work, future work is required to systematically review recruitment processes for clinical trials in palliative care. The use of search terms to identify eligible studies was limited by poor reporting of recruitment processes and details regarding mental capacity of eligible participants [55]. To address this, we used supplementary processes of reference chaining and seeking recommendations from experts of key studies. In line with international reporting guidelines [28, 140], researchers should provide detailed information about recruitment processes and differentiate between participants who consented for themselves and those requiring a proxy decision-maker. This would improve assessment of the success of the recruitment methods used to inform research practice [55]. While recommended research methods can be generalisable, these need to be tailored to the respective setting, context, and culture. Our findings were limited to predominantly Western contexts. This reflects the concentration of palliative care research in, for example, the USA and Europe [141]. Finally, the study included lay representatives in the workshops and Delphi Survey, but PPI in the data synthesis and voluntary sector representation was limited to membership of the Project Advisory Group. Greater exploration is required in areas directly impacting on patients and families, for example, the use of advance directives to record a person’s preferences for research participation (R21), and establishing a national body to provide support and information to family members acting as consultees (statement solution 12).

## Conclusions

To meet the increasing need for palliative care requires greater provision of evidence-based services and treatments, which is informed by research that includes adults from across the capacity spectrum. Our findings show that conducting research involving adults with impaired capacity is feasible and acceptable, and it is ethically unjust to exclude them. Inclusion requires careful planning of processes of recruitment that are aligned to varying and changeable levels of capacity, and the nature and intent of the research study to minimise risk of harm. Studies must ask research questions important for patients and families in the provision of services, care, and treatment in serious health-related illness. This review and TEC present innovative research methods, solutions on enhancing use, and critical consideration on implementation in research studies in different populations, clinical settings, and research designs. The MORECare_Capacity statement provides solutions underpinned by carefully considered evidence on involving and supporting consultees, consent processes across the capacity spectrum, and decision-making with adaptations for the respective care setting. The solutions have applicability for vulnerable patient populations in palliative care and beyond such as mental health and emergency medicine. The application of the solutions stated requires consideration within respective jurisdiction’s legislative framework. Future research is required on the applicability of the MORECare_C statement for non-Western cultures and low- to middle-income countries.

## Supplementary information

**Additional file 1: Table S1.** PRISMA statement and checklist of items.

**Additional file 2: Additional methods [Tables S2-S4].** Table S2: Systematic review electronic search terms for the respective database. Table S3: Study design categories using the Cochrane Effective Practice and Organisation of Care taxonomy. Table S4: Systematic review data extraction template.

**Additional file 3: Additional results for the systematic review [Tables S5-S8; suppl. 9].** Table S5: Qualsyst quality assessment of the included quantitative studies. Table S6: Qualsyst quality assessment of the included qualitative studies. Table S7: Reported study designs categorised by the Cochrane Effective Practice and Organisation of Care taxonomy. Table S8: Public attitudes and ethical issues in recruiting adults across the capacity spectrum. Suppl. 9: Additional reporting on public attitudes and ethical issues.

**Additional file 4: Additional results for the Transparent Expert Consultation [S10-S15].** Table S10: Delphi Survey participants in round one and round two. Table S11: Delphi Survey round one recommendations and level of consensus. Fig. S12: Delphi Survey box and whisker plots round one recommendations. Table S13: Delphi Survey round two recommendations and level of consensus. Fig. S14: Delphi Survey box and whisker plots round two recommendations. Box S15: Expert ‘think-tank’ - equivocal areas and the priority solutions.

## Data Availability

All data analysed for this review are included in this published article and its additional files.

## Abbreviations

AD: Alzheimer’s disease
A&E: Accident and emergency
AMI: Acute myocardial infarction
CTIMP: Clinical Trial of an Investigational Medicinal Product
DMC: Decision-making capacity
EIC: Enhanced informed consent
EoLC: End of life care
EPOC: The Effective Practice and Organisation of Care
GP: General practitioner
HCP: Healthcare professional
ICU: Intensive care unit
IPOS-Dem: Integrated Palliative care Outcome Scale–Dementia
IRB: Institutional review board
LMICs: Low- to middle-income countries
MacCAT-CR: MacArthur competence assessment tool for clinical research
MCA: Mental Capacity Act 2005
MRC: Medical Research Council
MMSE: Mini-Mental State Examination
NoK: Next of kin
Non-CTIMP: Clinical Trial of an Investigational Non-medicinal Product
PRISMA: Preferred Reporting Items for Systematic Reviews and Meta-Analyses
QoIC: Quality of informed consent
RCT: Randomised controlled trial
SD: Standard deviation
TEC: Transparent expert consultation
