# Supplementary material for: Processes of consent in research for adults with impaired mental capacity nearing the end of life: systematic review and transparent expert consultation (MORECare_Capacity statement)
Source: BMC Med. 2020 Jul 22;18:221. doi: 10.1186/s12916-020-01654-2 (PMC7374835; doi:10.1186/s12916-020-01654-2)
Supplement: Supplementary file 2 — Additional file 2: Additional methods [Tables S2-S4]. Table S2: Systematic review electronic search terms for the respective database. Table S3: Study design categories using the Cochrane Effective Practice and Organisation of Care taxonomy. Table S4: Systematic review data extraction template. [file 12916_2020_1654_MOESM2_ESM.docx]

# Additional file 2: Additional methods [Tables S2-S4]

## Table S2: Systematic review search terms by the respective database

|  | **Medline search - Palliative care** |
| --- | --- |
|  | terms |
| 1 | Exp palliative care/ |
| 2 | Exp Terminal Care/ |
| 3 | Exp Terminally Ill/ |
| 4 | palliat*.mp |
| 5 | Terminal* |
| 6 | Advanced.mp |
| 7 | Disease* mp or Disease/ |
| 8 | Ill*mp |
| 9 | 5 OR 6 |
| 10 | 7 OR 8 |
| 11 | 9 AND 10 (advanced/disease /terminal/ill) |
| 12 | Cancer* .mp |
| 13 | Malignan* .mp |
| 14 | End of life. mp |
| 15 | End stage. mp |
| 16 | Last year of life. mp |
| 17 | Life's end. mp |
| 18 | Exp hospice care/ |
| 19 | Hospice care .mp |
| 20 | Exp hospices/ |
| 21 | Hospice* .mp |
| 22 | 1 or 2 or 3 or 4 or 11 or 12 or 13 or 14 or 15 or 16 or 17 or 18 or 19 or 20 or 21 |
| 23 | Exp decision making |
| 24 | Decsion making .mp |
| 25 | Capacity .mp |
| 2 | Exp mental competency |
| 6 | Mental incompetency .mp |
| 27 | Mental incompetence .mp |
| 28 | Incapacitate* .mp |
| 29 | End of life decision* .mp |
| 30 | 23 or 24 or 25 or 26 or 27 or 28 or 29 |
| 30 | Exp Informed consent |
| 31 | Exp presumed consent |
| 32 | Exp Third-party consent |
| 33 | Consent* .mp |
| 34 | Assent .mp |
| 35 | 30 or 31 or 32 or 33 or 34 |
| **36** | **22 and 30 and 35** |
|  | **Medline search - Mental health** |
|  | **terms** |
| 1 | Exp Mental disorders/ |
| 2 | Exp cerebrovascular disorders/ |
| 3 | Exp learning disabilities/ |
| 4 | Exp Prader-Willi Syndrome |
| 5 | Exp Cognition disorders |
| 6 | Exp Nervous systems disorders |
| 7 | Neurologic* .mp |
| 8 | Exp delirium |
| 9 | Exp confusion/ |
| 10 | 1 or 2 or 3 or 4 or 5 or 6 ro 7 or 8 or 9 |
| 11 | Exp decision making |
| 12 | Decsion making .mp |
| 13 | Capacity .mp |
| 14 | Exp mental competency |
| 15 | Mental incompetency .mp |
| 16 | Mental incompetence .mp |
| 17 | Incapacitate* .mp |
| 18 | 11 or 12 or 13 or 13 or 14 or 15 or 16 or 17 |
| 19 | Exp Informed consent |
| 20 | Exp presumed consent |
| 21 | Exp Third-party consent |
| 22 | Consent* .mp |
| 23 | Assent .mp |
| 24 | 19 or 20 or 21 or 22 or 23 |
| **25** | **10 and 18 and 24** |

|  | **Medline search – Emergency medicine /Critical care** |
| --- | --- |
|  | terms |
| 1 | Accident and Emergency .mp |
| 2 | Exp Emergency medicine |
| 3 | Exp intensive care |
| 4 | Exp intensive care units |
| 5 | Exp critical care |
| 6 | Exp myocardial infarction |
| 7 | Exp cerebrovascular disorders/ |
| 8 | Cerebral vascular accident* .mp |
| 9 | Exp accidents,traffic/ |
| 10 | Road traffic accident* .mp |
| 11 | Exp stroke/ |
| 12 | 1 or 2 or 3 or 4 or 5 or 6 or 7 or 8 or 9 or 10 or 11 |
| 13 | Exp decision making |
| 14 | Decsion making .mp |
| 15 | Capacity .mp |
| 16 | Exp mental competency |
| 17 | Mental incompetency .mp |
| 18 | Mental incompetence .mp |
| 19 | Incapacitate* .mp |
| 21 | 13 or 14 or 15 or 16 or 17 or 18 or 19 |
| 22 | Exp Informed consent |
| 23 | Exp presumed consent |
| 24 | Exp Third-party consent |
| 25 | Consent* .mp |
| 26 | Assent .mp |
| 27 | 22 or 23 or 24 or 25 or 26 |
| **28** | **12 and 21 and 27** |

|  | **PsychInfo - Palliative care** |
| --- | --- |
|  | terms |
| 1 | Exp palliative care/ |
| 2 | Death and dying .mp |
| 3 | Exp Terminally Ill/ |
| 4 | Exp terminally ill patients |
| 5 | Exp terminal cancer |
| 6 | palliat*.mp |
| 7 | Terminal* |
| 8 | Advanced.mp |
| 9 | Disease* mp or Disease/ |
| 10 | Ill*mp |
| 11 | 5 OR 6 |
| 12 | 7 OR 8 |
| 13 | 9 AND 10 (advanced/disease /terminal/ill) |
| 14 | Cancer* .mp |
| 15 | Malignan* .mp |
| 16 | End of life. mp |
| 17 | End stage. mp |
| 18 | Last year of life. mp |
| 19 | Life's end. mp |
| 20 | Hospice care .mp |
| 21 | Hospice* .mp |
| 22 | 1 or 2 or 3 or 4 or 5 or 6 or 13 or 14 or 15 or 16 or 17 or 18 or 19 or 20 or 21 |
| 23 | Exp decision making |
| 24 | Decsion making .mp |
| 25 | Capacity .mp |
| 26 | Exp cognitive impairment |
| 27 | Exp cognitive ability |
| 28 | Mental incompetency .mp |
| 29 | Mental incompetence .mp |
| 30 | Incapacitate* .mp |
| 31 | End of life decision* .mp |
| 32 | 23 or 24 or 25 or 26 or 27 or 28 or 29 or 30 or 31 |
| 33 | Exp Informed consent |
| 34 | Consent* .mp |
| 35 | Assent .mp |
| 36 | 33 or 34 or 35 |
| **36** | **22 and 32 and 36** |

|  | **PsychInfo search - Mental health** |
| --- | --- |
|  | terms |
| 1 | Exp Mental disorders/ |
| 2 | Exp cerebrovascular disorders/ |
| 3 | Exp learning disabilities/ |
| 4 | Exp Learning disorders |
| 5 | Exp Prader-Willi Syndrome |
| 6 | Exp Cognitive impairment |
| 7 | Exp Nervous system disorders |
| 8 | Exp Neurodegenerative diseases/ |
| 9 | Neurologic* .mp |
| 10 | Exp schizophrenia |
| 11 | Exp delirium |
| 12 | Exp mental confusion |
| 13 | 1 or 2 or 3 or 4 or 5 or 6 ro 7 or 8 or 9 or 10 or 11 or 12 |
| 14 | Exp decision making |
| 15 | Decsion making .mp |
| 16 | Capacity .mp |
| 17 | Exp cognitive ability |
| 18 | Mental incompetency .mp |
| 19 | Mental incompetence .mp |
| 20 | Incapacitate* .mp |
| 21 | 14 or 15 or 16 or 17 or 18 or 19 or 20 |
| 22 | Exp Informed consent |
| 23 | Consent* .mp |
| 24 | Assent .mp |
| 25 | 22 or 23 or 24 |
| **26** | **13 and 21 and 25** |

|  | **PsychInfo search - A & E/Intensive care** |
| --- | --- |
|  | terms |
| 1 | Accident and Emergency .mp |
| 2 | Exp Emergency services |
| 3 | Exp intensive care |
| 4 | Exp intensive care unit* .mp |
| 5 | Exp myocardial infarction |
| 6 | Exp cerebrovascular disorders/ |
| 7 | Cerebral vascular accident* .mp |
| 8 | Road traffic accident* .mp |
| 9 | Exp stroke/ |
| 10 | 1 or 2 or 3 or 4 or 5 or 6 or 7 or 8 or 9 |
| 11 | Exp decision making |
| 12 | Decsion making .mp |
| 13 | Capacity .mp |
| 14 | Exp cognitive ability |
| 15 | Mental incompetency .mp |
| 16 | Mental incompetence .mp |
| 17 | Incapacitate* .mp |
| 18 | 11 or 12 or 13 or 14 or 15 or 16 or 17 |
| 19 | Exp Informed consent |
| 20 | Consent* .mp |
| 21 | Assent .mp |
| 22 | 19 or 20 or 21 |
| **23** | **10 and 18 and 22** |

|  | | **EMBASE - Palliative care** | |
| --- | --- | --- | --- |
|  | | terms | |
| 1 | | Palliative care .mp | |
| 2 | | Exp Terminal Care/ | |
| 3 | | Exp Terminally Ill patient | |
| 4 | | palliat*.mp | |
| 6 | | Exp Terminal disease | |
| 5 | | Terminal* | |
| 6 | | Advanced.mp | |
| 7 | | Disease* mp | |
| 8 | | Ill*mp | |
| 9 | | 5 OR 6 | |
| 10 | | 7 OR 8 | |
| 11 | | 9 AND 10 (advanced/disease /terminal/ill) | |
| 12 | | Cancer* .mp | |
| 13 | | Malignan* .mp | |
| 14 | | End of life. mp | |
| 15 | | End stage. mp | |
| 16 | | Last year of life. mp | |
| 17 | | Life's end. mp | |
| 18 | | Exp dying | |
| 19 | | Exp hospice care/ | |
| 20 | | Hospice care .mp | |
| 21 | | Exp hospice/ | |
| 22 | | Hospice* .mp | |
| 23 | | 1 or 2 or 3 or 4 or 5 or 11 or 12 or 13 or 14 or 15 or 16 or 17 or 18 or 19 or 20 or 21 or 22 | |
| 24 | | Exp decision making | |
| 25 | | Decsion making .mp | |
| 26 | | Capacity .mp | |
| 27 | | Exp mental capacity | |
| 28 | | Mental competency .mp | |
| 29 | | Mental incompetency .mp | |
| 30 | | Mental incompetence .mp | |
| 31 | | Incapacitate* .mp | |
| 32 | | End of life decision* .mp | |
| 33 | | 24 or 25 or 26 or 27 or 28 or 29 or 30 or 31 or 32 | |
| 34 | | Exp Informed consent | |
| 35 | | Consent* .mp | |
| 36 | | Assent .mp | |
| 37 | | 34 or 35 or 36 | |
| **38** | | **23 and 33 and 37** | |
|  | **EMBASE - Mental health** | |  |
|  | terms | |  |
| 1 | Exp Mental disease/ | |  |
| 2 | Exp cerebrovascular disease/ | |  |
| 3 | Exp learning disorder/ | |  |
| 4 | Exp Prader-Willi Syndrome | |  |
| 5 | Exp Cognitive defect | |  |
| 6 | Nervous systems disorders .mp | |  |
| 7 | Neurologic* .mp | |  |
| 8 | Exp delirium | |  |
| 9 | Exp confusion/ | |  |
| 10 | 1 or 2 or 3 or 4 or 5 or 6 ro 7 or 8 or 9 | |  |
| 11 | Exp decision making | |  |
| 12 | Decsion making .mp | |  |
| 13 | Capacity .mp | |  |
| 14 | Exp Mental capacity | |  |
| 15 | Exp mental competency | |  |
| 16 | Mental incompetency .mp | |  |
| 17 | Mental incompetence .mp | |  |
| 18 | Incapacitate* .mp | |  |
| 19 | 11 or 12 or 13 or 14 or 15 or 16 or 17 or 18 | |  |
| 20 | Exp Informed consent | |  |
| 21 | Consent* .mp | |  |
| 22 | Assent .mp | |  |
| 23 | 20 or 21 or 22 | |  |
| **24** | **10 and 19 and 23** | |  |

|  | **EMBASE search - A & E/Intensive care** |
| --- | --- |
|  | terms |
| 1 | Accident and Emergency .mp |
| 2 | Exp Emergency medicine |
| 3 | Exp intensive care |
| 4 | Exp intensive care unit |
| 5 | Critical care .mp |
| 6 | Exp heart infarction |
| 7 | Exp cerebrovascular disease/ |
| 8 | Exp Cerebrovascular accident/ |
| 9 | Exp traffic accident/ |
| 10 | Road traffic accident* .mp |
| 11 | Exp stroke/ |
| 12 | 1 or 2 or 3 or 4 or 5 or 6 or 7 or 8 or 9 or 10 or 11 |
| 13 | Exp decision making |
| 14 | Decsion making .mp |
| 15 | Capacity .mp |
| 16 | Exp Mental capacity |
| 17 | Exp mental competency |
| 18 | Mental incompetency .mp |
| 19 | Mental incompetence .mp |
| 20 | Incapacitate* .mp |
| 21 | 13 or 14 or 15 or 16 or 17 or 18 or 19 ro 20 |
| 22 | Exp Informed consent |
| 23 | Consent* .mp |
| 24 | Assent .mp |
| 25 | 20 or 21 or 22 |
| **26** | **12 and 21 and 25** |

| **CINAHL search - Palliative care** | | |
| --- | --- | --- |
| terms | | |
| MM (major concept) palliative care | | |
| Exp Terminal Care/ | | |
| Exp Terminally Ill Patients/ | | |
| palliat*.mp | | |
| Terminal* | | |
| Advanced.mp | | |
| Disease* mp or Disease/ | | |
| Ill*mp | | |
| 5 OR 6 | | |
| 7 OR 8 | | |
| 9 AND 10 (advanced/disease /terminal/ill) | | |
| Cancer* .mp | | |
| Malignan* .mp | | |
| End of life. mp | | |
| End stage. mp | | |
| Last year of life. mp | | |
| Life's end. mp | | |
| MM (major concept) Hospice care | | |
| Hospice care .mp | | |
| MM (major concept) hospices | | |
| Hospice* .mp | | |
| 1 or 2 or 3 or 4 or 11 or 12 or 13 or 14 or 15 or 16 or 17 or 18 or 19 or 20 or 21 | | |
| Exp decision making | | |
| Decsion making .mp | | |
| Capacity .mp | | |
| Exp mental competency | | |
| Mental incompetency .mp | | |
| Mental incompetence .mp | | |
| Incapacitate* .mp | | |
| End of life decision* .mp | | |
| 23 or 24 or 25 or 26 or 27 or 28 or 29 | | |
| MM (major concept) Consent (Research) | | |
| Presumed consent .mp | | |
| MM (major concept) Research Subject Recruitment | | |
| Third-party consent .mp | | |
| Consent* .mp | | |
| Assent .mp | | |
| 30 or 31 or 32 or 33 or 34 or35 | | |
| **22 and 30 and 36** | | |
|  | **CINAHL search - Mental health** |  |
|  | terms |  |
| 1 | Exp Mental disorders/ |  |
| 2 | Exp cerebrovascular disorders/ |  |
| 3 | Exp learning disorders/ |  |
| 4 | MM (major concept) Prader-Willi Syndrome |  |
| 5 | Exp Cognition disorders |  |
| 6 | Exp Nervous system abnormalities |  |
| 7 | Neurologic* .mp |  |
| 8 | Exp delirium, dementia, amnestic, cognitive disorders |  |
| 9 | Exp confusion/ |  |
| 10 | 1 or 2 or 3 or 4 or 5 or 6 ro 7 or 8 or 9 |  |
| 11 | Exp decision making |  |
| 12 | Decsion making .mp |  |
| 13 | Capacity .mp |  |
| 14 | Exp mental competency |  |
| 15 | Mental incompetency .mp |  |
| 16 | Mental incompetence .mp |  |
| 17 | Incapacitate* .mp |  |
| 18 | 11 or 12 or 13 or 14 or 15 or 16 or 17 |  |
| 19 | MM (major concept) Consent (Research) |  |
| 20 | Presumed consent .mp |  |
| 21 | MM (major concept) Research Subject Recruitment |  |
| 22 | Third-party consent .mp |  |
| 23 | Consent* .mp |  |
| 24 | Assent .mp |  |
| 25 | 19 or 20 or 21 or 22 or 23 ro 24 |  |
| **26** | **10 and 18 and 25** |  |

|  | **Medline search - A & E/Intensive care** |
| --- | --- |
|  | terms |
| 1 | Accident and Emergency .mp |
| 2 | MM (major concept) Emergency medicine |
| 3 | Intensive care .mp |
| 4 | Intensive care units .mp |
| 5 | Exp critical care |
| 6 | Exp myocardial infarction |
| 7 | Exp cerebrovascular disorders/ |
| 8 | Cerebral vascular accident* .mp |
| 9 | MM (major concept) accidents,traffic/ |
| 10 | Road traffic accident* .mp |
| 11 | MM (major concept) stroke |
| 12 | 1 or 2 or 3 or 4 or 5 or 6 or 7 or 8 or 9 or 10 or 11 |
| 13 | Exp decision making |
| 14 | Decsion making .mp |
| 15 | Capacity .mp |
| 16 | Exp mental competency |
| 17 | Mental incompetency .mp |
| 18 | Mental incompetence .mp |
| 19 | Incapacitate* .mp |
| 21 | 13 or 14 or 15 or 16 or 17 or 18 or 19 |
| 22 | MM (major concept) Consent (Research) |
| 23 | Presumed consent .mp |
| 24 | MM (major concept) Research Subject Recruitment |
| 25 | Third-party consent .mp |
| 26 | Consent* .mp |
| 27 | Assent .mp |
| 28 | 22 or 23 or 24 or 25 or 26 or 27 |
| **29** | **12 and 21 and 28** |

## Table S3: Study design categories using the Cochrane Effective Practice and Organisation of Care taxonomy

| **Category number** | **Study design description** |  |
| --- | --- | --- |
| A1 | Systematic reviews which include at least one Randomised Controlled Trial (i.e. Cochrane review) | |
| A2 | Other systematic and high-quality reviews which synthesis studies |  |
| A3 | Individual Randomised controlled Trials |  |
| B2 | Individual experimental/intervention non-randomised studies |  |
| B3 | Individual non-experimental studies, controlled statistically if appropriate, includes studies using case control. Longitudinal, cohort, matched pairs, or cross-sectional random sample methodologies and sound qualitative studies; analytical studies included |  |
| C1 | Descriptive and other research or evaluation not in B |  |
| **Evidence from expert opinion** | | |
| C2 | Case studies and examples of good practice |  |
| D | Summary review articles and discussions of relevant literature and conference proceedings not otherwise classified |  |
| E | Professional opinion base on practice, or reports of committees |  |
| F | User opinions from patients, carers or carers organisations |  |
| Source: Cochrane Effective Practice and Organisation of Care (2002). Data collection checklist. Ottowa, Ontario, Cochrane Effective Practice and Organisation of Care. | | |

Table S4: Systematic review data extraction template

| **General Information** | Include/Exclude |
| --- | --- |
|  | Reason for exclusion (if applicable) |
|  | Reviewer initials |
|  | Bibliographic details |
|  | Publication date |
|  | Year(s) study was undertaken |
|  | Country of origin |
|  | Quality appraisal score |
|  | EPOC grade |
|  | Coding tree theme |
| **Methods** | Data source e.g. Grey lit, SR, citations, experts |
|  | Type of review/study design |
|  | Clinical area |
|  | Study aim |
|  | Ethical approval |
|  | Population |
|  | Sample description |
|  | Setting |
|  | Intervention |
|  | Length of study |
|  | Data collection time points |
|  | Outcome measures used |
|  | Sample size calculation |
|  | Number participants approached |
|  | Number participants included |
|  | Number participants excluded |
|  | Number of included participants randomised (if applicable) |
|  | Attrition (number and reason) |
|  | Mean age of participants (if noted) |
|  | Process of consent |
|  | Inclusion criteria |
|  | Exclusion criteria |
|  | Assessment of capacity |
|  | Lack mental capacity (Y/N?NA) |
|  | Disease type (if applicable) |
|  | Disease stage (if applicable) |
|  | Data analysis |
| **Results** | Key findings regarding consent and/or capacity |
|  | Key findings of paper |
|  | Research challenges |
| **Discussion** | Study strengths |
|  | Study limitations |
|  | Recommendations to further research methods |
|  | Recommendations to further practice |
| **Citations** | Links to bibliographic references |
|  | Number of articles that cite work (at time of search) |
|  | Links from citations |
