# Supplementary material for: Processes of consent in research for adults with impaired mental capacity nearing the end of life: systematic review and transparent expert consultation (MORECare_Capacity statement)
Source: BMC Med. 2020 Jul 22;18:221. doi: 10.1186/s12916-020-01654-2 (PMC7374835; doi:10.1186/s12916-020-01654-2)
Supplement: Supplementary file 3 — Additional file 3: Additional results for the systematic review [Tables S5-S8; suppl. 9]. Table S5: Qualsyst quality assessment of the included quantitative studies. Table S6: Qualsyst quality assessment of the included qualitative studies. Table S7: Reported study designs categorised by the Cochrane Effective Practice and Organisation of Care taxonomy. Table S8: Public attitudes and ethical issues in recruiting adults across the capacity spectrum. Suppl. 9: Additional reporting on public attitudes and ethical issues. [file 12916_2020_1654_MOESM3_ESM.docx]

# Additional files 3: Additional results for the systematic review [Tables S5-S8, and suppl. 9]

Table S5: QualSyst quality assessment of the included quantitative studies by the respective main results’ area [35]

| Author, year | Question/objective sufficiently described? | Study design evident and appropriate? | Method of subject selection described and appropriate? | Subject characteristics sufficiently described? | Interventional random allocation possible, and described? | Interventional and blinding of investigators possible and described? | Interventional and blinding of subjects possible and described? | Outcome measures defined and robust? | Sample size appropriate? | Analytical methods described/justified and appropriate? | Estimate of variance reported? | Controlled for confounding? | Results reported in sufficient detail? | Conclusions supported by results? | Rating | Total |
| --- | --- | --- | --- | --- | --- | --- | --- | --- | --- | --- | --- | --- | --- | --- | --- | --- |
| **Studies innovating research methods to recruit adults across the capacity trajectory** | | | | | | | | | | | | | | | | |
| Adamis, 2005[46] | 2 | 2 | 2 | 2 | 2 | 1 | 1 | 2 | 1 | 2 | 1 | 1 | 2 | 2 | High | 0.77 |
| Adamis, 2010 [69] | 2 | 1 | 1 | 2 | N/A | N/A | N/A | 1 | 2 | 2 | 0 | 0 | 2 | 2 | Med | 0.68 |
| Agarwal, 1996 [76] | 1 | 2 | 2 | 1 | 2 | 2 | 2 | 2 | 1 | 1 | 0 | 0 | 1 | 1 | Med | 0.64 |
| Black, 2007 [52] | 2 | 1 | 1 | 2 | N/A | N/A | N/A | 1 | 2 | 2 | 2 | 1 | 1 | 1 | High | 0.73 |
| Bolcic-Jankovic, 2014 [74] | 2 | 2 | 2 | 2 | N/A | N/A | N/A | 1 | 2 | 2 | 0 | 0 | 2 | 2 | High | 0.77 |
| Buckles, 2003 [51] | 1 | 1 | 1 | 2 | N/A | N/A | N/A | 1 | 1 | 2 | 0 | 2 | 2 | 2 | High | 0.77 |
| Campbell, 2017 [57] | 2 | 1 | 1 | 1 | N/A | N/A | N/A | 2 | 1 | 2 | 2 | N/A | 1 | 2 | High | 0.75 |
| Chouliara, 2004 [62] | 1 | 1 | 2 | 2 | N/A | N/A | N/A | 1 | 2 | 1 | 1 | 0 | 2 | 2 | Med | 0.68 |
| Dobratz, 2003 [49] | 2 | 2 | 1 | 2 | N/A | N/A | N/A | 1 | 2 | 1 | 0 | 0 | 2 | 2 | Med | 0.68 |
| Ford, 2008 [61] | 2 | 2 | 2 | 2 | 2 | 2 | 0 | 2 | 2 | 2 | 0 | 2 | 2 | 2 | High | 0.86 |
| Fowell, 2006 [77] | 1 | 2 | 1 | 2 | 2 | 0 | 0 | 1 | 1 | 1 | 0 | 0 | 2 | 2 | Med | 0.54 |
| Gainotti, 2010* [44] | 1 | 0 | 1 | 2 | N/A | N/A | N/A | 1 | 1 | 1 | 2 | 1 | 2 | 1 | Med | 0.59 |
| Gysels, 2013 [64] | 2 | 2 | 2 | 0 | N/A | N/A | N/A | 2 | 1 | 1 | 2 | 0 | 2 | 2 | High | 0.73 |
| Harnell, 2012 [54] | 2 | 1 | 1 | 2 | N/A | N/A | N/A | 2 | 2 | 2 | 2 | 2 | 2 | 2 | High | 0.9 |
| Honarmand, 2018 [70] | 2 | 2 | 2 | 2 | N/A | N/A | N/A | 1 | 1 | 2 | 2 | 1 | 2 | 2 | High | 0.86 |
| Jeste, 2008 [55] | 2 | 2 | 2 | 2 | 1 | 2 | 0 | 2 | 2 | 2 | 2 | 2 | 2 | 2 | High | 0.89 |
| Karlawish, 2008 [72] | 2 | 1 | 2 | 2 | N/A | N/A | N/A | 2 | 2 | 1 | 0 | 0 | 2 | 2 | High | 0.73 |
| Kim, 2011a [71] | 1 | 1 | 1 | 2 | N/A | N/A | N/A | 1 | 2 | 2 | 2 | 2 | 2 | 2 | High | 0.82 |
| Levine, 2017 [78] | 1 | 1 | 2 | 1 | N/A | N/A | N/A | 1 | 1 | 1 | 0 | N/A | 1 | 1 | Med | 0.50 |
| Mittal, 2007 [53] | 2 | 2 | 2 | 2 | 0 | 0 | 0 | 2 | 1 | 2 | 2 | 2 | 2 | 2 | High | 0.75 |
| Morán-Sánchez, 2016 [47] | 2 | 2 | 2 | 2 | N/A | N/A | N/A | 2 | 2 | 2 | 2 | 0 | 2 | 2 | High | 0.91 |
| Moser, 2005 [56] | 2 | 1 | 2 | 1 | N/A | N/A | N/A | 2 | 2 | 2 | 2 | 0 | 2 | 1 | High | 0.77 |
| Olazaran, 2012 [65] | 2 | 1 | 1 | 2 | N/A | N/A | N/A | 1 | 2 | 2 | 0 | 0 | 2 | 2 | Med | 0.68 |
| Palmer, 2018 [58] | 2 | 2 | 2 | 2 | 1 | 2 | 0 | 2 | 1 | 2 | 0 | 0 | 2 | 2 | High | 0.71 |
| Rikkert, 1997 [60] | 1 | 1 | 2 | 1 | N/A | N/A | N/A | 1 | 2 | 2 | 1 | 0 | 2 | 1 | Med | 0.64 |
| Rubright, 2010 [59] | 1 | 2 | 2 | 1 | 2 | 2 | 0 | 1 | 1 | 2 | 2 | 2 | 1 | 1 | Med | 0.71 |
| Siminoff, 2004 [119] | 2 | 1 | 2 | 1 | N/A | N/A | N/A | 1 | 2 | 1 | 0 | 0 | 1 | 1 | Med | 0.55 |
| Sudore, 2006 [63] | 2 | 2 | 1 | 2 | NA | NA | NA | 2 | 1 | 2 | 2 | 1 | 2 | 2 | High | 0.86 |
| Thomalla, 2017 [48] | 2 | 1 | 2 | 1 | N/A | N/A | N/A | 1 | 2 | 2 | 0 | 1 | 2 | 2 | High | 0.73 |
| Warren, 1986 [73] | 1 | 1 | 1 | 1 | N/A | N/A | N/A | 1 | 2 | 2 | 0 | 0 | 2 | 1 | Med | 0.55 |
| **Studies applying consent processes across the capacity trajectory in serious illness** | | | | | | | | | | | | | | | | |
| Abernathy, 2006 [84] | 2 | 2 | 1 | 2 | 2 | N/A | N/A | 2 | 2 | 2 | 2 | 2 | 1 | 1 | High | 0.88 |
| Baskin, 1998 [93] | 2 | 1 | 2 | 1 | N/A | N/A | N/A | 1 | N/A | 1 | 0 | N/A | 1 | 2 | Med | 0.61 |
| Bench, 2015 [99] | 2 | 2 | 2 | 2 | 2 | 2 | N/A | 2 | 2 | 2 | 2 | 2 | 1 | 2 | High | 0.96 |
| Black, 2006 [88] | 2 | 1 | 2 | 2 | N/A | N/A | N/A | 1 | N/A | 2 | 2 | 1 | 2 | 1 | High | 0.8 |
| Botker, 2018[102] | 2 | 2 | 2 | 2 | 2 | 1 | N/A | 1 | 2 | 2 | 2 | 2 | 2 | 2 | High | 0.92 |
| Cole, 2002 [92] | 2 | 2 | 1 | 2 | 2 | 2 | 1 | 1 | 1 | 2 | 2 | 2 | 2 | 2 | High | 0.86 |
| Davies, 2010 [97] | 1 | 2 | 2 | 2 | 2 | 0 | N/A | 2 | 2 | 2 | 2 | 2 | 2 | 2 | High | 0.88 |
| Davies, 2018 [86] | 1 | 1 | 2 | 1 | N/A | N/A | N/A | N/A | 2 | 0 | N/A | N/A | 2 | 2 | Med | 0.69 |
| Day, 2015 [98] | 1 | 1 | 2 | 1 | N/A | N/A | N/A | 1 | 2 | 1 | N/A | N/A | 2 | 1 | Med | 0.67 |
| Galeotti, 2012*[45] | 2 | 2 | 1 | 0 | N/A | N/A | N/A | 2 | 1 | 1 | N/A | N/A | 2 | 2 | High | 0.72 |
| Gardiner, 2013 [80] | 1 | 2 | 1 | 1 | N/A | N/A | N/A | 2 | 1 | 2 | 0 | 0 | 2 | 1 | Med | 0.59 |
| Hanson, 2010 [141] | 2 | 2 | 2 | 1 | 1 | N/A | N/A | 1 | 2 | 1 | 0 | 0 | 1 | 1 | Med | 0.58 |
| Henwood, 2014 [115] | 2 | 2 | 2 | 1 | N/A | N/A | N/A | 1 | 1 | 1 | 2 | N/A | 1 | 2 | Med | 0.68 |
| Ho, 2018 [104] | 1 | 1 | 2 | 2 | N/A | N/A | N/A | 1 | 1 | 1 | 0 | N/A | 1 | 2 | Med | 0.6 |
| Irwin, 2008 [105] | 2 | 1 | 1 | 2 | N/A | N/A | N/A | 2 | 1 | 1 | 2 | N/A | 1 | 1 | High | 0.7 |
| Marcantonio, 2010 [91] | 2 | 2 | 2 | 2 | 1 | 1 | N/A | 2 | 2 | 2 | 2 | 2 | 2 | 2 | High | 0.92 |
| Mason, 2006 [81] | 0 | 1 | 1 | 1 | 1 | N/A | N/A | 1 | 1 | 1 | 0 | 0 | 1 | 0 | Low | 0.33 |
| Mitchell, 2006 [94] | 1 | 1 | 1 | 2 | N/A | N/A | N/A | 2 | 1 | 1 | 2 | 1 | 1 | 1 | Med | 0.64 |
| Myers, 2018 [82] | 2 | 1 | 1 | 2 | N/A | N/A | N/A | 1 | 2 | 1 | 0 | N/A | 2 | 1 | Med | 0.65 |
| Offerman, 2013 [83] | 2 | 2 | 1 | 2 | N/A | N/A | N/A | 2 | 1 | 2 | 2 | 1 | 1 | 2 | High | 0.82 |
| Ramerman, 2018 [103] | 2 | 1 | 2 | 2 | N/A | N/A | N/A | 1 | 2 | 2 | 2 | N/A | 2 | 2 | High | 0.9 |
| Rees, 2003 [96] | 2 | 1 | 1 | 0 | 0 | N/A | N/A | 2 | 1 | 1 | N/A | N/A | 1 | 1 | Med | 0.5 |
| Rouzé, 2017 [101] | 1 | 1 | 1 | 2 | 2 | N/A | N/A | 2 | 1 | 2 | 2 | 2 | 2 | 2 | High | 0.83 |
| Sampson, 2015 [42, 43] | 2 | 2 | 2 | 2 | N/A | N/A | N/A | 1 | 2 | 2 | 2 | 1 | 2 | 2 | High | 0.9 |
| Sampson, 2018 [40, 41] | 1 | 2 | 2 | 2 | N/A | N/A | N/A | 1 | 1 | 2 | 2 | N/A | 1 | 2 | High | 0.73 |
| Warner, 2007 [79] | 2 | 2 | 1 | 2 | 1 | 1 | 1 | 1 | 1 | 2 | 2 | 1 | 2 | 2 | High | 0.82 |
| Whelan, 2013 [85] | 2 | 1 | 2 | 1 | N/A | N/A | N/A | 2 | 2 | 2 | 2 | N/A | 0 | 1 | Med | 0.68 |
| **Studies on public attitudes and ethical issues in recruiting adults across the capacity spectrum** | | | | | | | | | | | | | | | | |
| Barrett, 2012 [128] | 2 | 1 | 2 | 2 | N/A | N/A | N/A | 2 | 2 | 2 | 2 | N/A | 2 | 1 | High | 0.9 |
| Bravo, 2008 [133] | 1 | 1 | 2 | 2 | N/A | N/A | N/A | 2 | 2 | 2 | 1 | 1 | 2 | 1 | High | 0.77 |
| Bravo, 2013 [132] | 2 | 2 | 1 | 1 | N/A | N/A | N/A | 1 | 2 | 2 | 1 | 1 | 1 | 2 | High | 0.73 |
| Burns, 2011 [129] | 2 | 2 | 1 | 1 | N/A | N/A | N/A | 2 | 2 | 2 | 1 | N/A | 2 | 1 | High | 0.75 |
| Cahill, 2000 [138] | 1 | 1 | 1 | 1 | N/A | N/A | N/A | 1 | 2 | 1 | 0 | 0 | 1 | 1 | Low | 0.45 |
| Farber, 2004 [134] | 2 | 2 | 1 | 2 | N/A | N/A | N/A | 2 | 2 | 2 | 2 | N/A | 2 | 1 | High | 0.9 |
| Gong, 2010 [75] | 1 | 2 | 1 | 1 | N/A | N/A | N/A | 1 | 2 | 1 | 0 | 0 | 1 | 2 | Med | 0.55 |
| Kamarainen, 2012 [127] | 1 | 1 | 2 | 2 | N/A | N/A | N/A | 2 | 1 | 2 | 0 | 0 | 2 | 2 | Med | 0.68 |
| Karlawish, 2002 [137] | 2 | 2 | 1 | 1 | N/A | N/A | N/A | 1 | 2 | 1 | 0 | N/A | 1 | 1 | Med | 0.6 |
| Karlawish, 2009 [131] | 2 | 2 | 1 | 2 | N/A | N/A | N/A | 2 | 2 | 2 | 2 | N/A | 2 | 2 | High | 0.95 |
| Kim, 2005 [120] | 2 | 2 | 2 | 2 | N/A | N/A | N/A | 1 | 2 | 2 | 2 | N/A | 2 | 1 | High | 0.9 |
| Kim, 2009 [135] | 2 | 2 | 1 | 2 | 1 | N/A | N/A | 2 | 2 | 2 | 2 | 2 | 2 | 1 | High | 0.88 |
| Kim, 2011b [130] | 1 | 2 | 1 | 2 | 1 | N/A | N/A | 2 | 2 | 1 | 1 | 2 | 2 | 1 | High | 0.75 |
| Peterson, 2003 [140] | 2 | 1 | 2 | 2 | N/A | N/A | N/A | 2 | 2 | 1 | 0 | 0 | 1 | 2 | Med | 0.68 |
| Van Beinum, 2017 [126] | 1 | 2 | 1 | 0 | N/A | N/A | N/A | 1 | 1 | 1 | 0 | 0 | 1 | 2 | Low | 0.45 |

* Articles reporting the same study

Table S6: QualSyst quality assessment of the included qualitative studies by the respective main results’ area [35]

| Author, year | Question/objective sufficiently described? | Study design evident and appropriate? | Context for study clear? | Connection to a theoretical framework/ wider body of knowledge? | Sampling strategy described and relevant? | Data collection methods described and systematic? | Data analysis described and systematic? | Verification procedures used? | Conclusions supported by the results | Reflexivity of account | Rating | Total |
| --- | --- | --- | --- | --- | --- | --- | --- | --- | --- | --- | --- | --- |
| **Studies innovating research methods to recruit adults across the capacity trajectory** | | | | | | | | | | | | |
| Boxall, 2016 [121] | 2 | 2 | 1 | 1 | 1 | 2 | 2 | 0 | 2 | 0 | Medium | 0.65 |
| Carey, 2017 [67] | 2 | 2 | 2 | 2 | 2 | 2 | 2 | 0 | 1 | 0 | High | 0.75 |
| Cowdell, 2008 [118] | 2 | 2 | 2 | 2 | 2 | 2 | 1 | 0 | 1 | 2 | High | 0.8 |
| Dunning, 2012 [68] | 2 | 2 | 1 | 1 | 0 | 1 | 1 | 0 | 1 | 1 | Medium | 0.5 |
| Hughes, 2015 [66] | 2 | 1 | 2 | 1 | 2 | 2 | 0 | 0 | 2 | 1 | Medium | 0.65 |
| Mangset, 2008 [50] | 2 | 2 | 2 | 1 | 2 | 1 | 2 | 0 | 2 | 0 | High | 0.7 |
| Smith, 2013 [124] | 2 | 1 | 2 | 0 | 1 | 1 | 1 | 0 | 2 | 0 | Medium | 0.5 |
| **Studies applying consent processes across the capacity trajectory in serious illness** | | | | | | | | | | | | |
| Ellis-Smith, 2018 [95] | 2 | 2 | 2 | 2 | 2 | 2 | 2 | 2 | 2 | 1 | High | 0.95 |
| Goodman, 2011 [89] | 1 | 1 | 2 | 0 | 2 | 1 | 0 | 0 | 2 | 0 | Low | 0.45 |
| Higginson, 2016 [100] | 2 | 2 | 2 | 1 | 2 | 2 | 1 | 2 | 2 | 0 | High | 0.8 |
| **Public attitudes and ethical issues in recruiting adults across the capacity spectrum** | | | | | | | | | | | | |
| Ali, 2006 [122] | 2 | 2 | 1 | 2 | 2 | 1 | 0 | 2 | 2 | 0 | High | 0.7 |
| Buckley, 2016 [125] | 2 | 2 | 2 | 2 | 2 | 1 | 1 | 0 | 2 | 1 | High | 0.7 |
| De Vries, 2013 [136] | 2 | 2 | 2 | 0 | 2 | 2 | 2 | 0 | 2 | 0 | High | 0.7 |
| Hamilton, 2017 [123] | 2 | 1 | 2 | 1 | 1 | 2 | 1 | 2 | 1 | 0 | Medium | 0.65 |
| Kleiderman, 2012 [117] | 1 | 2 | 2 | 1 | 2 | 2 | 1 | 2 | 2 | 0 | High | 0.75 |
| Van der Vorm, 2009 [139] | 2 | 2 | 1 | 1 | 1 | 1 | 1 | 1 | 2 | 0 | Medium | 0.6 |
| Wood, 2013 [116] | 2 | 2 | 2 | 1 | 1 | 2 | 1 | 0 | 1 | 0 | Medium | 0.6 |

Table S7: Reported study design for eligible studies categorised by the Cochrane Effective Practice and Organisation of Care taxonomy [36]

|  |  | Number of articles (n=88 studies) |
| --- | --- | --- |
| A3 | Individual RCTs | 20 [45, 46, 48, 54, 55, 58, 77, 79, 81, 85-87, 90-93, 96, 99, 101, 102] |
| B2 | Individual experimental/intervention non-randomised studies | 3 [56, 61, 95] |
| B3 | Individual non-experimental studies, controlled statistically if appropriate, includes studies using case control. Longitudinal, cohort, matched pairs, or cross-sectional random sample methodologies and sound qualitative studies; analytical studies included | 36 [41, 43, 49, 51, 53, 57, 63, 67-70, 75, 80, 82, 83, 88, 89, 94, 97, 98, 100, 103, 104, 115, 116, 118, 121, 123, 125, 126, 129, 130, 132, 138, 140, 142] |
| C1 | Descriptive and other research or evaluation not in B | 29 [47, 50, 52, 60, 62, 64, 66, 72-74, 76, 78, 105, 117, 119, 120, 122, 124, 127, 128, 131, 134-137, 139, 143-145] |
| Source: Cochrane Effective Practice and Organisation of Care (2002). Data collection checklist. Ottowa, Ontario, Cochrane Effective Practice and Organisation of Care. | | |

Table S8: Public attitudes and ethical issues in recruiting adults across the capacity spectrum (grouped by clinical area) (n=22 studies)

| **Authors, country, EPOC grade** | **Year** | | **Study design and aim** | | | **Setting** | | **Sample description** | | | **Consent process across the capacity spectrum** | | **No. patients/**  **Eligible (%)** | | **Key findings, challenges and solutions** |
| --- | --- | --- | --- | --- | --- | --- | --- | --- | --- | --- | --- | --- | --- | --- | --- |
| **Palliative care** | | | | | | | | | | | | | | | |
| **Wood, F et al. [116]**  **UK**  **B3** | 2013 | | Qualitative interviews to explore stakeholders' views on ethical and practical challenges associated with recruiting care home residents in research studies. | | | Care home | | | Care home residents and relatives; and GPs | | **Advance consent** | | 14 residents, 14 relatives and 10 GPs | | **Key findings:** Respondents were generally accepting of low risk observational studies and slightly less accepting of low risk RCTs of medicinal products. Although respondents identified practical barriers to informed consent, consenting arrangements were considered workable. Residents and relatives generally agreed that an advance consent model was acceptable, but opinions differed about the outcome if a resident lost capacity during the research study. |
| **Dementia and geriatric care** | | | | | | | | | | | | | | | |
| **Kim, S et al. [130]**  **USA**  **B3** | 2011 | | Telephone survey to assess the informed, deliberative views of the older general public on surrogate consent for research on Alzheimer Disease (AD) | | | N/A | | | General public aged 50 years and over | | **Surrogate consent** | | 700 | | **Key findings:** A policy of surrogate consent for AD research was supported by 55-91% depending on the scenario. The education group had a transient increase in support for one research scenario after receiving the information materials. In the deliberation group, support for surrogate consent was higher after deliberation for all scenarios (67-97%) **Solution:** Having deliberation sessions could increase people’s acceptance of surrogate consent. |
| **Kim, S et al. [120]**  **USA**  **C1** | 2005 | | Postal survey on the views of those at heightened risk of Alzheimer’s Disease (AD) about how they would balance the need for research in AD and protecting vulnerable subjects. | | | Postal survey | | | Participants enrolled in the Alzheimer's Disease Anti-inflammatory Prevention Trial | | **Surrogate consent** | | 229/259 (88.4%) | | **Key findings:** Over 90% found minimal risk studies and RCTs of new medications acceptable for surrogate consent. A smaller majority found the more invasive studies acceptable. Participants were more cautious when deciding for a loved one. General attitude toward biomedical research and scenario-specific risk perception were strong independent predictors. |
| **Karlawish, J et al. [131]**  **USA**  **C1** | 2009 | | Face-to-face interview on support for older adults enrolling non-competent persons with AD in research that does not benefit participants. | | | N/A | | | General public (older adults) | | **Advance consent and proxy leeway** | | 538 | | **Key findings:** 83% were willing to grant advance consent to a blood draw study, 48% to a blood draw plus lumbar puncture study. Most (96%) were willing to identify a proxy-decision maker, and most were willing to grant their proxy leeway over their advance consent: 81% for the blood draw study and 70% for the blood draw plus lumbar puncture study. Combining the preferences for advance consent and leeway, the proportion, who would permit being enrolled in the blood draw and lumbar puncture studies, respectively, were 92% and 75%. Multivariate modelling showed willingness for enrolment in research was associated with +ve attitude for biomedical research. |
| **Kim, S et al. [135]**  **USA**  **C1** | 2009 | | A survey to assess the views of a nationally representative, policy-relevant sample of the general public (older Americans) about surrogate consent for four research scenarios of varying degrees of risk and potential benefit. | | | Survey | | | General public (older, 51+ Americans) | | **Surrogate consent** | | 1515 | | **Key findings:** Most of the respondents supported allowing families to make surrogate consent decision for dementia research (67.5% to 82.5%) and would themselves, want to participate in surrogate-based research. Most would also grant some or complete leeway to their surrogates (54.8% to 66.8%), but this was true mainly of those willing to participate. There was a trend toward lower willingness to participate in surrogate-based research among those from ethnic or racial minorities. |
| **De Vries, R et al. [136]**  **USA**  **C1** | 2013 | | Democratic deliberation to describe how members of the older general public deliberate with one another in finding solutions to the dilemma of involving decisionally incapable persons in dementia research. | | | N/A | | | Persons aged 50+ | | **Surrogate consent** | | 160 | | **Key findings:** The older general public strongly support a policy of surrogate consent for dementia research and that this support increased after in-depth education and peer deliberation. |
| **Karlawish, J et al. [137]**  **USA**  **C1** | 2002 | | A survey to understand the process of informed consent at individual sites participating in a specific clinical trial in order to identify variations. | | | Survey | | | Alzheimer's disease clinical research centres | | **Informed consent** | | 30 | | **Key findings:** The survey reveals substantial variation in the practices of informed consent even within centres. These results suggest potential opportunities to develop and disseminate best practices. |
| **Cahill, M et al. [138]**  **USA** | 2000 | | Postal survey to determine if centres had policies or guidelines on research involving cognitively impaired persons and to ascertain the additional protections addressed in their policies/guidelines | | | Postal survey | | | Alzheimer’s Disease research centres | | **N/A** | | 29 | | **Key findings**: Seven main policy areas identified: assignment of surrogate decision makers; guidance on "competency" and procedures for assessing intellectual capacity; subject assent/dissent; protections for subjects based on IRB evaluation of research risks and benefits; use of ethics consultants or a "subject advocate"; selection of research subjects; periodic re-evaluation of Cognitive Capacity. 50% of respondents had no policies or guidelines about research involving cognitively impaired subjects, although several commented that their IRBs consider additional protections on a protocol-by-protocol basis. **Challenges**: Inconsistencies on ethical requirements for ALC with lack of clarity on policy/guidelines used by IRBs. The presence of a written policy does not assure that research subjects in the institution necessarily receive better protection. |
| **Van der Vorm, A et al. [139]**  **The Netherlands**  **B3** | 2009 | | A modified Delphi study to identify and compare the five ethical issues considered most important by expert panellists in non-genetic and genetic Alzheimer’s Disease research and (2) to compare findings with ethical issues in genetic research in general as described in the literature | | | N/A | | | Experts in the field of Alzheimer’s Disease, genetics or ethics research | | **Informed consent/ surrogate consent** | | 12 experts in the first round; 17 experts in the second round | | **Key findings:** In both genetic and non-genetic research, informed consent is considered the most important issue, but the ratings differ substantially. Whereas informed consent is considered by far the most important issue in non-genetic research, there is less consensus in genetic research about morally relevant topics. The main differences observed related to the position of family members and the status of and access to genetic information. |
| **Peterson, G. et al. [140]**  **Sweden**  **B3** | 2003 | | A survey to understand the attitudes of members of the regional independent ethics committees (IEC) in Sweden to a range of important ethical issues related to research involving Alzheimer’s Disease subjects. | | | Survey | | | Members of the Research Ethics Committees | | **Informed consent/ surrogate consent** | | 117 | | **Key findings:** 91% thought the investigator should determine if the AD patient had sufficient decisional capacity before asking them to participate in research. A significantly larger proportion of experts than lay persons were positive about a legal representative serving as a proxy (48%vs. 12%; p < .01). 72% of the respondents viewed that the investigator, before approving informed consent from a proxy, should determine whether the proxy had knowledge of the patient’s personal values earlier in life, e.g. attitude to participation in research projects. More laypersons than experts (100% vs. 75%, p < .05) and more women than men (100% vs. 71%, p < .01) held this opinion. |
| **Stroke** | | | | | | | | | | | | | | | |
| **Ali, K et al. [122]**  **UK**  **C1** | | 2006 | | Focus group on stroke patients’ and carers’ views on consent for a study on oxygen therapy exploring consent issues, acceptability of the outcome measures | Focus group meetings | | Stroke patients and carers | | | **Advice from relatives/ waiver of consent/ deferred** | | 73 | | **Key findings:** Participants were informed that the treatment (oxygen therapy) would have to be given within 24 hours of the stroke and that many patients are unable to give informed consent to a research study because of the effects of the stroke. The group was asked to comment on if these patients should be included in the study or not and who should give proxy-consent if the patient was unable to do so. General agreement that many stroke patients will be unable to give an informed consent, and proxy-decision making by relatives or a friend is acceptable. In the third meeting, most participants felt that inability to consent should not exclude patients from participation in the research study. 75% thought it appropriate for the family or carers to advise on behalf of an incompetent patient about study inclusion. 92% would allow a doctor to recruit an incompetent patient to the study and seek deferred consent or proxy-decision. | |
| **Mental health** | | | | | | | | | | | | | | | |
| **Hamilton, J et al. [123]**  **UK**  **B3** | | 2017 | | Cross-sectional survey to understand clinical research practice for establishing capacity in informed consent with adults with intellectual  disabilities and/or autism | NHS hospital and out-patient settings | | Clinical researchers working with adults with intellectual disabilities/autism | | | **Informed consent** | | 21/69 (30.4%) | | **Key findings:** Recruitment of adults lacking capacity was mainly through their consultees. The decision to advise inclusion in a study depended on engagement with the researcher and tasks to complete. 13 out of 18 studies the researchers reported excluded adults lacking capacity; there was no formal capacity assessment in the studies prior to exclusion of the individuals. In ten studies researchers judged the capacity of the individuals, whereas in eight studies capacity judgement made by a clinician (generally the referring clinician) or a family member. Agreed assessment of capacity for consent will be specific to the research study, which limits use of a formal standardized approach. **Solutions:** Use of easy read information sheets and aids for the consent process. Building relationships and explaining the study should be prioritized, and presence of a family member/carer. **Challenges:** Poor understanding on the notion of research and what was involved. | |
| **Intensive care** | | | | | | | | | | | | | | | |
| **Burns, K et al. [129]**  **Canada**  **B3** | | 2011 | | Self-complete survey to assess the general public's attitudes toward various consent models and data management strategies for critically ill adults, eligible to participate in a low-risk RCT. | Survey in public location | | General public | | | **Substitute decision making (informed or** **deferred consent** | | 217 | | **Key findings:** 64.7% of participants felt comfortable with a substitute decision maker providing consent on their behalf. 59.3% of participants felt comfortable with a substitute decision maker being asked whether the person would object to participating. 56.8% of participants felt comfortable with deferred consent in the presence of a substitute decision maker. Regardless of the consent scenario, 64% to 74% of respondents wanted to be considered for participation in a RCT if they were critically ill and unable to give informed consent. | |
| **Buckley, J et al. [125]**  **UK**  **B3** | | 2016 | | Qualitative interviews to investigate patients’ feelings on taking part in emergency medical research. | Hospital Admiss-ion Unit/ Surgical Assess-ment Centre | | Inpatients admitted with a medical emergency | | | **Deferred consent** | | 17 | | **Key Findings:** Most (16/17) interviewees were open to the idea of participating in research in emergency medical settings. Some interviewees stated that they felt uncomfortable with the idea of research without prior consent because it violated a patient’s autonomy. | |
| **Kamarainen, A et al. [127]**  **Finland**  **C1** | | 2012 | | Survey (1) post-RCT to understand the attitudes and experiences of surviving cardiac arrest victims recruited with surrogate consent into a pre-hospital RCT. (2) Survey of legal representatives and consent providers of both survivors and non-survivors. (3) Survey of emergency physicians on perceptions of emergency research and conduct of consent. | Home (survey) | | Cardiac arrest victims recruited to an earlier RCT at time of admission | | | **Surrogate consent** | | 11 patients, 17 consent providers, 13 physicians | | **Key findings:** All respondents carried positive attitudes towards the conducted pre-hospital trial. In the light of their experience, 71% of spouses were convinced that even in an emergency, consent needs to be obtained from the patients or next of kin to recruit a patient in a trial, whereas only 45% of patients and 46% of physicians agreed on this statement. A significant difference was observed about the perceived capability of the spouse to decide upon patient's enrolment - 88% of the spouses felt they could make that decision and all patients support this. 61% of physicians were doubtful of the decision-making capacity of the consent provider. All but one patient and 53% of the consent providers would have preferred contact from the researchers and additional information regarding the trial afterwards. | |
| **Barrett, K et al. [128]**  **Canada**  **C1** | | 2012 | | Structured interviews to examine the attitudes and preferences of surrogate decision makers about their involvement in the consent to research process. | Hospital/ICU | | Surrogate decision makers of patients receiving mechanical ventilation | | | **Surrogate decision making** | | 136 | | **Key findings:** Most surrogate decision makers wished to be involved in decision making (90%; 95% CI 84-95%), only 2% rated it as unacceptable (95% CI 1-6%). 50% were comfortable with involvement ( 95% CI 41-59%) but the proportion decreased as risk of harm increased (34%; 95% CI 26-43%) or enrolment window was shorter (41%; 95% CI 33-50%). 62% of surrogates reported symptoms of anxiety and 38% of depression. | |
| **Van Beinum, A et al. [126]**  **Canada**  **B3** | | 2017 | | Prospective observational study to describe the development of the ethical approach for the study, the process of review by four institutional research ethics boards across Canada and staff satisfaction with the study | Intensive care units at five study sites | | Critically ill and imminently dying patients, their families, and bedside clinical care team at each study site | | | **Surrogate consent** | | 76 clinicians returned satisfaction questionnaire, reporting 37 patients. | | **Consent process:** The study consent form was drafted with ethics and legal consultants to ensure clear, concise and accessible wording on study participation. The consent document was reviewed by patient advisors to ensure readability and comprehension. **Key findings:** 3/5 ethics boards requested the consent form emphasize that standard patient care, including the family’s ability to interact with dying patients, would not be influenced by study participation. 2/5 boards suggested that descriptions of the purpose of the study should be removed to avoid confusion or distress. 4/5 boards felt that the language used to describe death and the dying process was inappropriate suggesting terminology was ‘too harsh’, ‘technical or dismissive’. 87% enrolment and consent rate indicated feasible recruitment into the prospective study on EoLC in ICU **Solutions:** Physicians’ close relationship with the family enabled consent in research on EoLC. Ongoing collaboration of an inter-professional team was integral to the study’s success to develop study design, data collection and consent forms. | |
| **General population** | | | | | | | | | | | | | | | |
| **Bravo, G et al. [145]**  **Canada/France**  **C1** | | 2008 | | Anonymous postal questionnaires on knowledge, opinions, and practices about substitute consent for research across researchers | Acade-mia | | Two groups of researchers in France and Quebec | | | **Substitute consent** | | 122 | | **Key findings:** Legislative knowledge governing substitute consent was poor, with difference between French and Quebec researchers (p<0.001). Most respondents felt that the substitute decision-maker does not require legal appointment if the study poses little risk to the participant. Practice data showed disparity between the researchers’ processes of consenting participants and legal requirements. | |

| Gong, M et al. [75]  USA  B3 | 2010 | Cross-sectional survey to examine Institutional Review Board practices on surrogate consent and other safeguards to protect incapacitated adults in research. | Public/ IRBs | General population Institutional Review Boards (ethics) | Proxy/surrogate consent /Authorized proxy consent/assent, spouse or parent as surrogates, excluding adult children and other family  Adults with diminished capacity | 104 (out of 157 eligible IRBs) | Key findings and challenges: 104 IRBs reported that in the previous year they sometimes (49%), frequently (33%), or very frequently (2%) reviewed studies involving patients in ICU. Six IRBs (6%) do not accept surrogate consent for research from any persons and 22% of IRBs accept only an authorized proxy, spouse or parent as surrogates, excluding adult children and other family. IRBs vary in their limits on research risks in studies involving incapacitated adults: 15% disallow any research regardless of risk in studies without direct benefit, whereas 39% allow only minimal risks. When there was potential benefit, fewer IRBs limit the risk at minimal (11%; p < .001). Even in populations at high risk for impaired decision-making, many IRBs rarely or never required procedures to determine capacity (13%–21%). IRBs also varied in their use of independent monitors, research proxies and advance research directives. Solutions: Federal clarification of the issues of surrogate consent for research is needed before IRBs consistently accept surrogate consent for research. The common rule should be clarified to indicate that research involving incapacitated adults can occur after obtaining informed consent/proxy consent. It should define the hierarchy of acceptable surrogates. |
| --- | --- | --- | --- | --- | --- | --- | --- |

| **Farber, N et al. [134]**  **USA**  **C1** | 2004 | Cross-sectional survey of physicians on enrolling patients unable to give informed consent or considered vulnerable into clinical trials. | Postal survey | Randomly selected physicians in the USA | **No prior consent** | 400/1000 | **Key findings:** 84% of respondents indicated that at least one case scenario was acceptable. A majority of those who conduct clinical trials (62%), who had training in ethics of clinical research (78%), and who sit on IRBs (83%) approved of at least one case scenario. Physicians approved of the entry of some patients who cannot give informed consent or who are of a vulnerable population into clinical trials. |
| --- | --- | --- | --- | --- | --- | --- | --- |
| **Bravo, G et al. [132]**  **Canada**  **B3** | 2013 | Cross-sectional, postal survey to explore factors that may influence stakeholders’ decision about if to enrol a decisionally incapacitated close relative in a hypothetical research study. | Self-administered postal survey at a public setting | General public (including practitioners) | **Surrogate decision-making** | 2060/6300 | **Key findings:** Respondents' ranked factors associated with surrogate decision-making. All groups, potential for direct patient benefit ranked as the most influential (55.8%), followed by possibility of serious side effects (30.0%), with other five factors lower. More older adults considered direct benefits (60.8%). Lay persons included the reputation of the researchers and the benefits to themselves in top three (p<.001). Inconveniences to substitute decision maker was the least influential factor. Researchers trained in medicine were least likely to consider side effects (p=.002). Potential benefits of participation are more influential than potential risks in decision-making. **Challenges:** Surrogates do not always know what the patient would prefer, hence these factors play a role, if wishes are unknown. |
| **Kleiderman, E et al. [117]**  **Canada**  **C1** | 2012 | Qualitative study on clinician-researcher attitudes to recruit patients with advanced cancer into non-therapeutic research. | Hospital and research centres | 7 health professionals working with terminally ill advanced cancer patients | **Continuous/ process informed consent** | 7 | **Key findings:** (1) *Ethical considerations*: sub-themes; autonomy, respect for persons, beneficence, non-maleficence, discrimination, confidentiality and public good. (2) *Patient-centred issues*: sub-themes **Solutions;** communication, informed consent as a continuous process, skilled research nurse, individual contact, timing, family dynamics, **Challenges**; -ve research culture, patient vulnerability, and psychosocial issues. |

Supplement 9: Additional reporting on public attitudes and ethical issues

Studies on attitudes towards including adults lacking capacity in research consistently reported acceptability of involving consultees to enable participation, but with variance associated with the nature and purpose of the study. Most studies (59.1%) were conducted with public members (including researchers, healthy population, IRBs) exploring attitudes concerned research with critically ill patients (e.g. conducted in intensive or emergency care settings), and studies focusing on Alzheimer Disease, stroke, cancer, overall incapacitated individuals, surrogate decision-making for research, and intellectual disabilities. Study designs included observational (e.g. survey, qualitative interviews) and mixed methods with various parties including the general population, patients, family members, healthcare professionals, and institutional boards.

A survey in Canada of the general public (n=2060/6300) were asked to think of hypothetical scenarios where they would act as a consultee and rank factors influencing their likely decision [132]. Overall, benefits of participation were considered more influential than potential risks in decision making with potential benefit to the patient (or loved one) the most influential factor in their decision (55.8%), followed by possibility of side effects (30.0%) The burden to the consultee was the least influential factor [132]. Research studies on intensive and emergency care explored attitudes towards consultee assent and deferred consent (or deferred assent), and ethical considerations for the consent process [125-129, 142]. A survey exploring views from the general public (n=217) about low-risk RCTs involving critically ill adults showed acceptability (64.7%) for a substitute decision maker to provide decisions on participation on their behalf, and using processes of assent (59.3%) and deferred consent (56.8%) [129]. Importantly, most respondents (64% to 74.0%) indicated preference to be considered for an RCT if they were critically ill and unable to provide informed consent. Three studies used qualitative and quantitative methods to explore consent processes in emergency medical research with patients (n=17) who had received emergency care [125], recovered from acute myocardial infarction (AMI) (n=30) [142], or participated in a RCT and were surveyed on consent processes involving patients (n=11), surrogate decision makers (n= 17) for both patients who recovered and those who died, and physicians (n=13) [127]. The studies concurred that most participants were open to the idea of research taking place in emergency settings, receiving some of the information about the study after the consent process, and their family members advising about participation on their behalf. However, opinions varied on acceptability of a deferred consent, and participating in a procedural trial without a priori consent. In the RCT survey most of the family members (n=12/17, 71%) considered a priori consent should be obtained either from the patient or themselves. In contrast only 45% of patients and 46% of physicians agreed with this statement [127]. Views regarding deferred consent were influenced by the trial type. Family members indicated acceptability of involvement in research decision-making, but this decreased with higher risk RCTs and when were required to decide in a shorter time period [128]. Researchers assert that to enable involvement of family members as surrogate decision makers requires use of straightforward, non-technical language, and the need for context-sensitive approaches that account for both clinical acuity and the characteristics of the trial [142].

Acceptability of consultee decision making is echoed in research on dementia and geriatric care. In a survey of individuals (n=229) at heightened risk of Alzheimer’s Disease, 90.0% reported acceptability of surrogate decision making to participate in both minimal risk studies and clinical trials of new medication trials, but acceptability decreased for more invasive trials [120]. In a democratic deliberation study with older members of public, Similar findings were obtained from older adults regarding the use of advance and process consent (n=160), strong support for a policy of consultee advice for dementia research was given [136] increased after in-depth education and peer deliberation. where 92.0% (n=495), and 75.0% (n=404) of the respondents found this consent type acceptable for venepuncture and lumbar puncture studies, respectively [131]. In both studies, respondents’ attitudes towards biomedical research and risk perception were predictors. In a study exploring general public’s attitudes towards dementia research, 67.5% to 82.5% of the respondents were supportive of consultee decision making [135]. A trend of lower willingness to participate in surrogate-based research was observed in people from ethnic or racial minorities.

Five studies exploring healthcare professionals’ and researchers’ views on the involvement of consultees iterate variance in acceptability depending on the population and the nature of the study. Researchers conducting research with older people agreed that consultees should be involved and felt that the consultees do not have to be legally appointed, especially when the study posed little risk to the participant [145]. Healthcare professionals (n=21) involved in studies with individuals with intellectual disabilities highlighted the importance of easy read information sheets both for the individual and their family members, memory aids, and use of process consent, while formal capacity assessment was considered unnecessary [123]. In another study, 84.0% of the healthcare professionals who work with vulnerable populations (n=400), considered it acceptable to enter patients who cannot provide informed consent in clinical trials [134]. When consultee assent for research in vulnerable populations were explored with researchers (n=122), they did not see requirement for a legally appointed consultee, especially for low risk studies [145]. A qualitative study with clinician-researchers (n=7) working with terminally ill cancer patients highlighted importance of process consent, ethical considerations such as autonomy and beneficence, especially for non-therapeutic trials, and family, patient and clinician dynamics [117]. A challenge in this context was highlighting the distinction between care and research conversations, to ensure that patients and families do not feel pressured to participate in the study.

From a multi-centre study conducted with critically ill and imminently dying patients, where family members provided consultee assent, IRBs’ requirements for the consent processes were explored [126]. Three out of five IRBs recommended highlighting that the care of the patient would not be influenced by the study participation, while four out five IRBs thought that the language used to describe death and dying, and the purpose of the research study were inappropriate for the family members. Based on these recommendations, the study was able to achieve 87% consent rate with family members. Reiterating the previous findings, use of technical language should be minimised.

Acceptability of, and for, process consent was explored amongst care home residents and their relatives [116]. This consent model was considered workable and appropriate; while the amount of information participants wanted during the consent process varied. Respondents were also generally accepting of low risk observational studies, and less accepting of medicinal RCTs. Focus groups conducted with stroke patients and relatives showed high agreement for assent by a relative or a friend, and deferred consent as acceptable consent processes for a low risk treatment study [122].
