# Supplementary material for: Processes of consent in research for adults with impaired mental capacity nearing the end of life: systematic review and transparent expert consultation (MORECare_Capacity statement)
Source: BMC Med. 2020 Jul 22;18:221. doi: 10.1186/s12916-020-01654-2 (PMC7374835; doi:10.1186/s12916-020-01654-2)
Supplement: Supplementary file 4 — Additional file 4: Additional results for the Transparent Expert Consultation [S10-S15]. Table S10: Delphi Survey participants in round one and round two. Table S11: Delphi Survey round one recommendations and level of consensus. Fig. S12: Delphi Survey box and whisker plots round one recommendations. Table S13: Delphi Survey round two recommendations and level of consensus. Fig. S14: Delphi Survey box and whisker plots round two recommendations. Box S15: Expert ‘think-tank’ - equivocal areas and the priority solutions. [file 12916_2020_1654_MOESM4_ESM.docx]

# Additional file 4: Additional results for the Transparent Expert Consultation [Tables S10, S11, S13; Fig. S12, S14; and Box S15]

## Table S10: Delphi Survey participants for round one and round two

| Participant category (researcher defined) | Number of invited participants | Round one respondents (%) | Round two respondents (%) |
| --- | --- | --- | --- |
| Clinical-academic | 17 | 10 (58.8) | 8 (80.0) |
| Researcher | 27 | 16 (59.2) | 13 (81.3) |
| Clinician | 9 | 1 (11.1) | 1 (100.0) |
| Service user/voluntary sector representative* | 29 | 15 (51.7) | 13 (86.7) |
| Policy maker | 1 | 1 (100.0) | 1 (100.0) |
| Total | 83 | 43 (51.8) | 36 (83.7) |
| *Voluntary sector representatives were lay members from charities including: Age UK, Alzheimer Society, Braintrust, Independent Cancer Patient Voice, National Council of Palliative Care People in Partnership Group, Marie Curie, and the National Cancer Research Institute. The lay members were themselves carers (currently or in the past), and/or living with a progressive condition. | | | |

## Table S11: Delphi Survey round one recommendations reported by the topic area and by level of consensus and agreement

| **ID** | **Recommendation by area (n=recommendations)** | **Mean (SD)** | **Median (IQR)** | **Recommendation consensus and agreement*** |
| --- | --- | --- | --- | --- |
|  | **Area 1: Timeliness and design of the consent process (n=7 recommendations)** | | | |
| **R2** | Information about a research study is comprehensible, short and written in accessible language | 8.66 (0.99) | 9  (9-9) | Indicated  Strict agreement |
| **R7** | The consent process is short and not burdensome | 8.12 (1.37) | 9  (7.5-9) | Indicated  Strict agreement |
| **R1** | Time is allowed for more than one discussion between patients and researchers, and families on what involvement in a research study means | 7.23 (2.13) | 8  (6-9) | Indicated  Broad agreement |
| **R10** | Researchers to seek an advance consent while individual shave capacity to consent for themselves, for example shortly after a diagnosis of a progressive illness | 7.19 (1.97) | 8  (6.25-9) | Indicated  Broad agreement |
| **R14** | For individuals who have capacity to consent in the moment, but overtime may not remember the discussion, an ongoing process of consent is used whereby researchers re-confirm the individual's wish to participate at each data collection time point | 7.35 (2.09) | 8  (6.5-9) | Indicated  Broad agreement |
| **R4** | Closer working between clinicians and researchers is required to identify the best time to seek consent from individuals to participate in a research project | 6.95 (1.50) | 7  (6-8) | Indicated  Broad agreement |
| **R6** | Researchers to make available to patients and/or carers a patient advocate or public representative to support them to decide if they wish to participate in a research study and in the consent process | 5.62 (2.16) | 6  (4-7) | Equivocal  Broad agreement |
|  | **Area 2: Enhancing consultee and supportive decision making (n=10 recommendations)** | | | |
| **R9** | The consent process if tailored to the individual's needs, capabilities and values with researchers observing for non-verbal and verbal cues that may indicate an individual may wish to withdraw | 8.23 (1.21) | 9  (8-9) | Indicated  Strict agreement |
| **R12** | Individuals and proxies to have clear and concise information from researchers and/or clinicians on opportunities to participate in research studies enabling them to choose if they wish to be involved | 8.05 (1.21) | 8  (7.25-9) | Indicated  Strict agreement |
| **R11** | In the advance consent process, a participant should be asked to designate a proxy (e.g. a family member) who - if the participant loses capacity - would be consulted by the researchers as to whether in their opinion the individual would have wished to continue to take part | 7.5 (1.76) | 8  (7-9) | Indicated  Strict agreement |
| **R21** | Advance care planning to include discussing and recording in a Statement of Wishes document an individual's nominated proxy decision maker whose opinion on participating in a research study is sought if the patient loses capacity | 7.42 (1.74) | 8  (7-9) | Indicated  Strict agreement |
| **R20** | Patients' preferences and wishes on involvement in research are discussed and recorded in advance care planning documents or living wills | 7.40 (1.73) | 8  (6.25-9) | Indicated  Broad agreement |
| **R8** | The process of informed consent to include engaging family members close to the individual | 6.95 (1.96) | 8  (5-9) | Indicated  Broad agreement |
| **R13** | Clarification on the nature and extent of the responsibility of a proxy acting on behalf of a patient including who is suitable to act as a participant's proxy, and how and when they are identified | 7.52 (1.89) | 8  (7-9) | Indicated  Broad agreement |
| **R22** | The term 'proxy' is changed in documents and discussions to 'closest person' or 'nominated person' | 6.52 (2.23) | 7  (5-8) | Indicated  Broad agreement |
| **R15** | Carers or professional consultees are encouraged to indicate to researchers their availability to act as a consultee on behalf of an individual | 6.12 (2.14) | 6.5  (5-7.75) | Equivocal  Broad agreement |
| **R17** | Health and social care practitioners to act as advocates for both participants and their proxies to support decision making on taking part in a research study | 5.10 (2.25) | 5  (4-7) | Equivocal  Broad agreement |
|  | **Area 3: Ethics, resources and expertise (n=12 recommendations)** | | | |
| **R23** | Health and social care practitioners to recognise research as a core clinical activity in a similar way as teaching and training | 7.63 (2.08) | 9  (7-9) | Indicated  Strict agreement |
| **R29** | Researchers to demonstrate to Research Ethics Committees a clear process of consent for potential participants with compromised capacity that details how the researchers will process to tailor the consent process to maximise individuals' ability to consent for themselves and when and how they will seek an opinion from a consultee | 7.50 (2.02) | 8  (7-9) | Indicated  Strict agreement |
| **R24** | Public awareness of research in palliative care is raised to enable individuals to indicate their interest in participation particularly during the earlier stages of a progressive or chronic illness | 7.55 (2.08) | 8  (7-9) | Indicated  Strict agreement |
| **R19** | Researchers to have timely access to experiences members of the research team to provide supervision on the consent process, particularly when an individual's level of capacity is uncertain or fluctuating | 7.58 (1.52) | 8  (7-9) | Indicated  Strict agreement |
| **R28** | Auditing of NHS Research Ethics Committees' decision making processes to ensure transparency and consistency on ethical approvals for studies involving adults lacking capacity | 7.44 (1.75) | 8  (6-9) | Indicated  Broad agreement |
| **R18** | Researchers and clinicians to maintain a database of individuals who may be interested in future research participation | 7.07 (2.11) | 8  (6-9) | Indicated  Broad agreement |
| **R5** | Clear guidance is needed on how best to approach potential participants for research in end of life care and how to present and discuss what participation involves | 7.21 (1.50) | 7  (6-9) | Indicated  Broad agreement |
| **R16** | Researchers to attend advanced communication training to increase their sensitivity and confidence when seeking to consent a patient to participate in a research study | 7.00 (1.80) | 7  (6.5-8) | Indicated  Broad agreement |
| **R3** | Increased support and training for clinicians is required to improve their skills in discussing research with patients during routine clinical contact | 7.02 (1.65) | 7  (6-8) | Indicated  Broad agreement |
| **R26** | The Mental Capacity Act 2005 to be amended to simplify the provisions on lasting powers of attorney | 6.20 (2.46) | 7  (5-8) | Indicated  Broad agreement |
| **R25** | The Mental Capacity Act 2005 to be amended allowing paid professional carers to act as a consultee for individuals who lack capacity to give an opinion on whether the individual would have wanted to participate in the research study had they had capacity to indicate this | 4.93 (2.55) | 5  (3-7) | Equivocal  Broad agreement |
| **R27** | Establishment of a nationally recognised body that provides support and information to family members and carers acting as proxies | 5.15 (2.58) | 5  (3-7) | Equivocal  Broad agreement |
| Abbreviations: SD - Standard deviation; IQR - Interquartile range. *Level of consensus and agreement categories by median regions and IQR detailed in table D1 | | | | |

## Figure S12: Delphi survey box and whisker plots round one recommendations (median and spread)


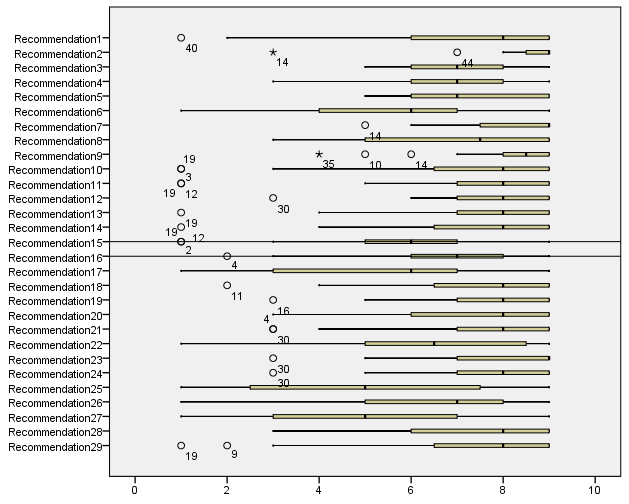


## Table S13: Delphi Survey round two recommendations reported by topic area and ordered by level of consensus and agreement

| ID | Recommendation by area and ordered by level of consensus high to low | Mean (SD) | Median (IQR) | Recommendation consensus and agreement* |
| --- | --- | --- | --- | --- |
|  | **Area 1:** **Timeliness and design of the consent process (n=7 recommendations)** | | | |
| R2 | Information about a research study is comprehensible, short and written in accessible language | 9.0 (0.17) | 9  (9-9) | Indicated  Strict agreement |
| R7 | The time taken for the consent process is proportional to the study and accommodates individual requirements (e.g. fatigue, cognition) | 8.0 (1.15) | 8  (8 – 9) | Indicated  Strict agreement |
| R4 | Closer working between clinicians, researchers and family members/carers is required to identify the best time to seek consent from individuals to participate in a research project | 7.6 (0.96) | 8  (7-8) | Indicated  Strict agreement |
| R1 | Time is available for more than one discussion between patients and researchers, and families on what involvement in a research study means according to individual requirements | 7.5 (1.3) | 8  (7 -8.25) | Indicated  Strict agreement |
| R10 | Although the legal significance of advance consent will vary depending on the relevant legal framework, it is good practice for researchers to seek an advance consent while individuals have capacity to consent for themselves, for example, shortly after a diagnosis of a progressive illness | 7.6 (1.76) | 8  (7 – 9) | Indicated  Strict agreement |
| R14 | For individuals who have capacity to consent in the moment, but overtime may not remember the discussion, an ongoing process of consent is used whereby researchers re-confirm the individual's wish to participate at each data collection time point | 7.6 (1.99) | 9  (6 – 9) | Indicated  Broad agreement |
| R6 | Researchers to make available to patients and/or carers a patient advocate or public representative to support them to decide if they wish to participate in a research study and in the consent process | 4.8 (2.13) | 5  (3 -6.25) | Equivocal  Broad agreement |
|  | **Area 2: Enhancing consultee and supportive decision making (n=10 recommendations)** | | | |
| R9 | The consent process is tailored to the individual's needs, capabilities and values with researchers observing for non-verbal and verbal cues that may indicate an individual may wish to withdraw | 8.8 (0.5) | 9  (9-9) | Indicated  Strict agreement |
| R12 | Individuals, consultees and/or proxies to have clear and concise information from researchers and/or clinicians on opportunities to participate in research studies enabling them to choose if they wish to be involved | 8.3 (0.72) | 8  (8-9) | Indicated  Strict agreement |
| R11 | In the advance consent process, a participant should be asked to designate a consultee (e.g. a family member) who - if the participant loses capacity - would be consulted by the researchers as to whether in their opinion the individual would have wished to continue to take part | 8.3 (1.05) | 9  (8-9) | Indicated  Strict agreement |
| R21 | Advance care planning to include discussing and recording in a Statement of Wishes document an individual's nominated or personal consultee whose opinion on participating in a research study is sought if the patient loses capacity | 7.6 (1.36) | 8  (7-8) | Indicated  Strict agreement |
| R20 | Patients' preferences and wishes on involvement in research are discussed and recorded in advance care planning documents or living wills | 7.9 (1.53) | 8.5  (7-9) | Indicated  Strict agreement |
| R13 | Clarification on the nature and extent of the responsibility of a consultee acting on behalf of a patient including who is suitable to act as a participant's consultee in clinical research, and how and when they are identified | 7.8 (1.26) | 8  (7-9) | Indicated  Strict agreement |
| R8 | The process of informed consent to include engaging family members close to the individual | 7.1 (2.04) | 8 (5.5 – 8.5) | Indicated  Broad agreement |
| R22 | The term 'proxy' is changed in documents and discussions to 'nominated person' | 7.0 (1.99) | 7  (6-8) | Indicated  Broad agreement |
| R15 | Carers or professional consultees are encouraged to indicate to researchers their availability to act as a consultee on behalf of an individual | 4.6 (1.95) | 5  (4-6) | Equivocal  Broad agreement |
| R17 | Health and social care practitioners to act as advocates for both participants, their consultee and/or proxies to support decision making on taking part in a research study | 4.7 (1.89) | 5  (4-6) | Equivocal  Broad agreement |
|  | **Area 3: Ethics, resources and expertise (n=12 recommendations)** | | |  |
| R29 | Researchers to demonstrate to Research Ethics Committees a clear process of consent for potential participants with compromised capacity that details how the researchers will proceed to tailor the consent process to maximise individuals' ability to consent for themselves and when and how they will seek an opinion from a consultee | 8.6 (1.40) | 9  (9-9) | Indicated  Strict agreement |
| R28 | Auditing of NHS Research Ethics Committees' decision-making processes to ensure transparency and consistency on ethical approvals for studies involving adults lacking capacity | 8.5 (1.35) | 9  (8.75-9) | Indicated  Strict agreement |
| R23 | Health and social care practitioners to recognise research as a core clinical activity in a similar way as teaching and training | 8.3 (1.73) | 9  (9-9) | Indicated  Strict agreement |
| R18 | Researchers and clinicians to maintain a database of individuals who may be interested in future research participation | 8.1 (1.3) | 9  (7-9) | Indicated  Strict agreement |
| R24 | Public awareness of research in palliative care is raised to enable individuals to indicate their interest in participation particularly during the earlier stages of a progressive or chronic illness | 8.0 (1.27) | 9  (7.75-9) | Indicated  Strict agreement |
| R19 | Researchers to have timely access to experienced members of the research team to provide supervision on the consent process, particularly when an individual's level of capacity is uncertain or fluctuating | 7.7 (1.41) | 8  (7.75-9) | Indicated  Strict agreement |
| R16 | Researchers to attend advanced communication training to increase their sensitivity and confidence when seeking to consent a patient to participate in a research study | 6.5 (2.06) | 7  (5-8) | Indicated  Broad agreement |
| R3 | Increased support and training for clinicians is required to improve their skills in discussing research with patients during routine clinical contact | 7.5 (1.40) | 7.5 (6.75-9) | Indicated  Broad agreement |
| R5 | Clear guidance is needed on how best to approach potential participants for research in end of life care and how to present and discuss what participation involves | 7.4 (1.36) | 7  (7-9) | Indicated  Broad agreement |
| R26 | The Mental Capacity Act 2005 to be amended to simplify the provisions on Lasting Powers of Attorney | 6.0 (2.41) | 7  (5-8) | Indicated  Broad agreement |
| R25 | The Mental Capacity Act 2005 to be amended allowing paid professional carers to act as a consultee for individuals who lack capacity to give an opinion on whether the individual would have wanted to participate in the research study had they had capacity to indicate this | 4.7 (2.5) | 5  (3-6) | Equivocal  Broad agreement |
| R27 | Establishment of a nationally recognised body that provides support and information to family members and carers acting as consultees and/or proxies | 4.5 (2.25) | 5  (3-6) | Equivocal  Broad agreement |
| Abbreviations: SD - Standard deviation; IQR - Interquartile range. *Level of consensus and agreement categories by median regions and IQR detailed in table D1 | | | | |
|  | | | | |

## Figure S14: Delphi survey box and whisker plots round two recommendations (median and spread)


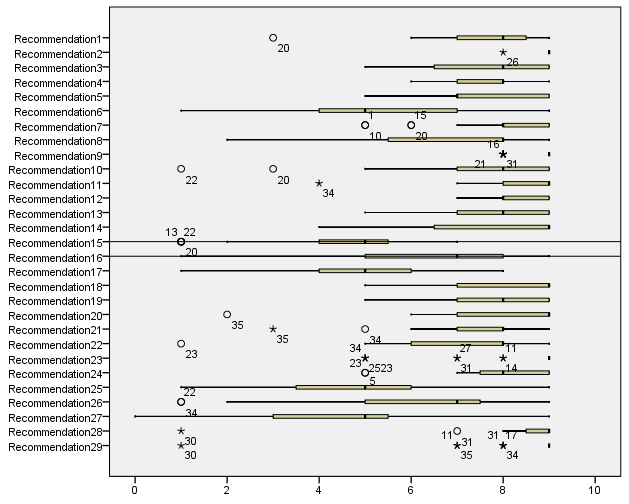


## Box S15: Expert ‘think-tank’ – three equivocal areas and the priority solutions

**Area 1) Involving and supporting consultees in the decision-making process.** Uncertainty surrounded how best to support consultees to engage in the research process, with four recommendations in the Delphi survey round two indicating equivocal broad agreements (R 6, 8, 15, 22, 27). These included health and social care practitioners acting as an advocate for a patient and personal consultee to support decision-making on study participation (R6). Rather than an advocate, a public representative or research link was proposed to empower individual decision-making and minimise paternalism which ‘advocate’ may imply. Engaging family members in the informed consent process (R8), encouraging potential consultees to indicate availability of researchers (R15), and establishing a nationally recognised body to support family members acting as consultees (R27). Concerns were expressed about the practicalities of resources and funding.

***Area 1 solutions:***

- Revise guidance for the Mental Capacity Act 2005 [1] to make the role of the consultee more flexible – the way nominated consultee are identified and used, and the advice they give to researchers. Changing guidance is more achievable than changing legislation.
- Design research in ways that supports family members’ and carers’ involvement in research (e.g. as personal consultees) and acknowledges the importance of family views/conflict in the decision-making process.

**Area 2) Practitioner training and education; the challenges and solutions.** Agreement was apparent on the requirement to increase training and support for researchers and clinicians, but areas of uncertainty in the Delphi survey concerned how to approach eligible participants and communicate study details (R3), and supervise research processes (R19). Uncertainties focused on how best to provide and disseminate training and guidance and fund. Participants agreed on the following: “*Clear guidance is needed on how best to approach potential participants for research in end of life care and how to present and discuss what participation involves”* (R5), but guidance needed to be specific, and short, and utilise online learning resources to increase access.

***Area 2 solutions:***

- Good Clinical Practice (GCP) standard of research training for all researchers aligned to the study design and detailed as a research governance requirement.
- Involve service user representatives in all areas of the research process to increase researchers’ understanding of experiences of patients & families.
- Research should be ‘normalised’ as a core component of health and social care and not as something ‘hazardous’.

**Area 3) Legislative frameworks; incorporation into research practice and limitations.** The greatest area of contention in the Delphi survey surrounded the Mental Capacity Act 2005 [1] legislation in England and Wales, and recommendations that the supporting guidance for the Act to provide more explicit detail on the application of the Act for research studies (R25, R26). For example, “*The Mental Capacity Act 2005 is amended to allow paid professional carers to act as a consultee for adults lacking capacity”* (R25). Concerns centred on conflicts of interest, time limitations, and a formal carer not knowing the individual when the person had capacity to express their wishes about research participation.

***Area 3 solutions:***

- Guidelines should act as a framework. They cannot always anticipate change in an individual’s circumstances or their wishes. Ongoing review is required by the consultee and researchers of an individual’s best interests. Advance consent should not be legally binding as in European CTIMP legislation [106], but incorporate ongoing review if a participant loses capacity.
- Opportunities for independent people to volunteer to act as a consultee after undergoing training and agreement to maintain confidentiality. However, this may limit the extent the advice given is informed by an individual’s wishes.
